# Defining and navigating macrocycle chemical space†

**DOI:** 10.1039/d0sc05788f

**Published:** 2021-03-04

**Authors:** Lauren A. Viarengo-Baker, Lauren E. Brown, Anna A. Rzepiela, Adrian Whitty

**Affiliations:** Department of Chemistry, Boston University 590 Commonwealth Ave Boston Massachusetts 02215 USA whitty@bu.edu; Center for Molecular Discovery, Boston University 24 Cummington Mall Boston Massachusetts 02215 USA; Pyxis Discovery Delftechpark 26 Delft 2628XH The Netherlands

## Abstract

Macrocyclic compounds (MCs) are of growing interest for inhibition of challenging drug targets. We consider afresh what structural and physicochemical features could be relevant to the bioactivity of this compound class. Using these features, we performed Principal Component Analysis to map oral and non-oral macrocycle drugs and clinical candidates, and also commercially available synthetic MCs, in structure–property space. We find that oral MC drugs occupy defined regions that are distinct from those of the non-oral MC drugs. None of the oral MC regions are effectively sampled by the synthetic MCs. We identify 13 properties that can be used to design synthetic MCs that sample regions overlapping with oral MC drugs. The results advance our understanding of what molecular features are associated with bioactive and orally bioavailable MCs, and illustrate an approach by which synthetic chemists can better evaluate MC designs. We also identify underexplored regions of macrocycle chemical space.

## Abstract 2

Macrocyclic compounds (MCs) are of high interest for inhibition of challenging drug targets, but existing oral MC drugs occupy regions of chemical space that are not well sampled by many available synthetic MC chemotypes.

## Graphical abstract

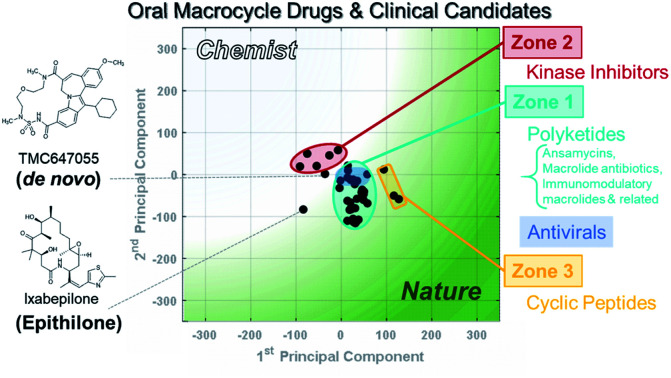

## Introduction

There is increasing interest in drug chemotypes that violate accepted ideas of druglikeness, driven by the goal to target poorly druggable proteins for which conventional small molecule compounds have historically been ineffective.^1,2^ Due to the nature of the available binding sites,^3^ these challenging targets, typified by certain protein–protein interactions (PPI), often require high MW ‘beyond Rule of 5’ (bRo5) ligands to achieve high affinity binding. Historically, however, high MW compounds have been associated with poor pharmaceutical properties, including poor prospects for oral bioavailabilty.^4,5^

Macrocyclic compounds (MCs) – typically defined as organic compounds containing a ring of ≥12 atoms – are a chemotype of particular current interest.^1,6–15^ Certain MCs appear to achieve superior ADME (Absorption, Distribution, Metabolism, and Excretion) properties compared to acyclic compounds of comparable MW.^7,11,12,16–20^ Moreover, MCs can make a large contact interface with their protein receptors, spanning widely spaced binding energy hot spots,^2^ and consequently can bind topologically flat sites such as are common at PPI interfaces.^1^ Based on these observations, we,^2,3,21^ and others,^1,6,8,11,12,14,16,22–24^ have hypothesized that MCs represent a privileged chemotype for binding and inhibiting PPI targets. The pharmaceutical utility of MCs is established by the fact that 82 have been approved as drugs, including 30 known to achieve systemic distribution when administered orally, with many others in clinical development.^1,2,8,11–13,15,25,26^ Of these MC drugs and clinical candidates, the vast majority are bRo5 compounds, with properties that are distinct from those of conventional small molecule drugs.^1,2,27^ There has been considerable recent progress in our understanding of factors that contribute to the oral bioavailability of cyclic peptides^16–18,28–34^ but far less has been done to understand the properties of nonpeptidic MCs.

Medicinal chemists have benefited from the existence of guidelines for the design of conventional small molecule drugs, and there have been attempts to develop analogous guidelines for MCs.^2,7,35^ As one approach to this problem, several studies have aimed to define the structural and physicochemical properties of MC drugs.^1,2,7,14^ For example, Over *et al.* compared 200 synthetic MCs from the Broad Institute's diversity-oriented screening library to all oral drugs and to the subset of oral drugs that violate the Ro5, to identify determinants of cell permeability and oral absorption.^7^ Their work elucidated substructures, substituents, and molecular properties that impact permeability. However, prior studies aimed at defining MC features characteristic of oral MC drugs have generally considered the compounds in terms of existing molecular descriptors that were originally developed to characterize conventional small molecules, and which fail to capture some features of MC chemotypes that could be relevant to their pharmacological behavior. As a result, the specific properties that enable good pharmaceutical properties in MCs remain poorly understood, presenting a substantial obstacle to the effective use of synthetic MCs for drug discovery.

In the current work, we use the machine learning technique of Principal Component Analysis (PCA) to map the locations of selected synthetic MC collections and oral and non-oral MC drugs and clinical candidates in structural and physicochemical property space. Doing so allows us to assess the extent of MC property space each compound set encompasses, and where each set is located with respect to the MC drugs. A distinctive feature of our approach, compared to previous work,^7,36^ is that, to construct this property space, we devise multiple new molecular descriptors to capture previously ignored features unique to MC structures that could be important for their pharmacological behavior. Our results demonstrate that these new descriptors capture substantial new and non-redundant information about MC structures and properties, enabling a more nuanced discrimination within and between MC chemotypes. The analysis shows that the oral MC drugs and clinical candidates define three adjacent regions of structure–property space, and that the synthetic MC chemotypes included in this study have minimal overlap with these regions. We test different strategies for designing and assessing modified MC designs that are more “MC druglike,” and identify a set of 13 key properties, and the associated value ranges that coincide with occupancy of “druglike MC space”.

## Results

### Development of MC-specific molecular descriptors

A large number of molecular descriptors (MolDs) have been devised to describe the structural and physicochemical features of organic compounds.^37–39^ However, there are properties specific to MCs that we believe could be important for their pharmacological behavior, and for which no suitable descriptor has previously been formulated. We therefore developed a set of additional decsiptors to capture these MC-specific features.

**Fig. 1:**
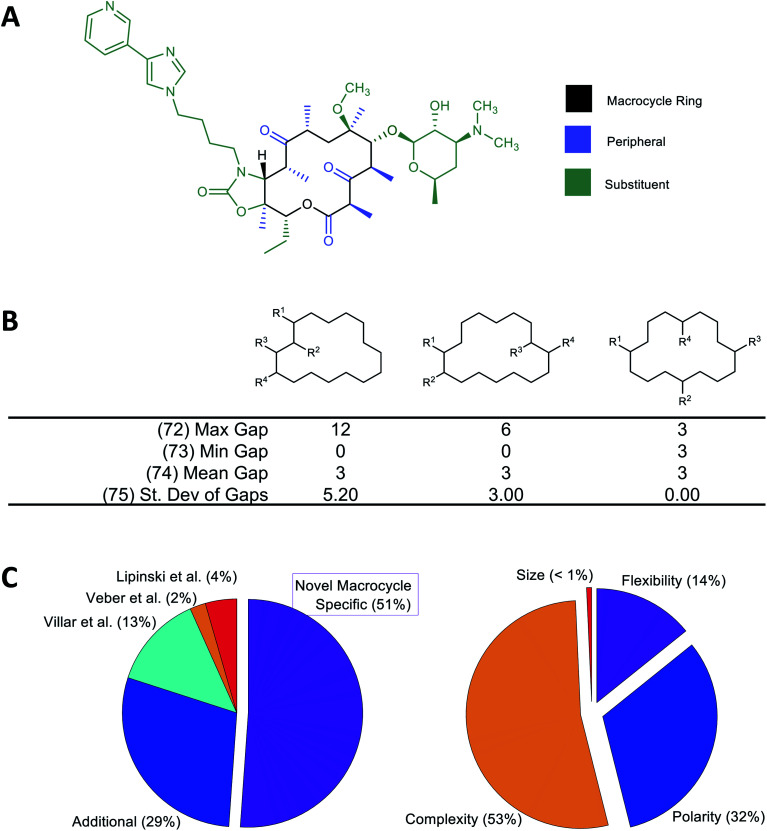
Molecular descriptors used in the study. (A) Following our earlier work,^2^ we consider the atoms of the macrocyclic molecule to belong to one of three positional classes: atoms comprising the macrocycle ring itself, atoms that are part of small groups containing just single heavy atoms appended directly to the ring (“peripheral atoms”), and atoms belonging to groups of ≥2 heavy atoms attached to the macrocycle ring (“substituent atoms”). (B) Example calculation of MolDs 72–75, capturing different aspects of how the substituents are arranged around the MC ring. R^1^–R^4^ are substituents. A “Gap” is defined as the number of ring atoms between the attachment points of a pair of substituents. Numbers in parentheses refer to the MolD numbers (see Supplementary document A†). (C) Origins of the 90 MolDs in the set used in this study (left), and what aspects of MC properties they describe (right).

We have previously shown that binding of MCs to their protein targets can be usefully considered in terms of which contacts are with atoms on the MC substituents, with atoms in the MC ring itself, or with single heavy-atom moieties attached to the ring (*e.g.* methyl, hydroxyl, carbonyl groups) that we term “peripheral groups” (Fig. 1A).^2^ We therefore included in our MolD set descriptors capturing the number of heavy atoms contained in the MC ring, in the substituents, and in the peripheral groups, and the elemental composition of these three regions of the molecule. Additionally, we hypothesized that the sizes and size distribution of substituents, and whether the substituents are clustered together or distributed around the MC ring, could potentially affect pharmacological properties. Similarly, whether the substituents are connected to the ring in a rigid or rotatable manner, as well as features that affect the flexibility of the MC ring itself such as double bonds and fused rings, could be important. We therefore also formulated descriptors capturing these features. As an example, Fig. 1B illustrates a group of MolDs that capture whether the substituents are evenly distributed around the ring or clustered together, based on counting the ring atoms that comprise the “gap” between the connection points of adjacent substituents. For some descriptors, we included features both as counts and, separately, normalized by ring size, in case this quantity relative to the size of the ring or the whole molecule proved more important than the absolute value of the property.

Overall, we developed 46 new MC-specific descriptors. These were combined with 12 MolDs embodying guidelines for MC design we suggested in previous work,^2^ plus 32 well-known existing descriptors which, while not uniquely applicable to MCs, describe molecular properties generally relevant to drug-likeness. These included the four MolDs that appear in the Ro5 (ref. 4) and the two that define Veber's rules^40^ (Fig. 1C). The applicability of these classic Ro5 and Veber descriptors to macrocycles is, in some cases, questionable. For example, the computed octanol–water partition coefficient, clog *P*, calculated on the basis of chemical structure, does not necessarily reflect the true conformation-dependent lipophilicity of a complex MC. We nonetheless retained this descriptor because it captures aspects of the atom composition of the compounds that could be important. Similarly, by Veber's definition, sigma bonds in a macrocycle ring are not considered rotatable. We nevertheless retained Number of Rotatable Bonds (NRB) as a descriptor to capture the flexibility of the substituents attached to the MC ring. The 90 MolDs that comprise our final set are described in Supplemental Document A.

The MolD set includes only descriptors that can be calculated from the two-dimensional chemical structure of the compound. It is well-established that the behavior of MCs can be influenced by their three-dimensional conformation.^19,41–45^ However, despite considerable recent progress,^41,42,46–49^ the conformational analysis of complex MCs remains an unsolved problem, hindering the accurate calculation of conformation-dependent molecular properties. In the current work we chose to focus on MolDs that can be applied to analysis of massive virtual MC libraries, for which any kind of conformational analysis would be impractical, while recognizing that the results we obtain by this approach will tell only part of the story. The current approach is thus intended as a coarse filter that could serve as a prelude to the application of more computationally expensive tools to a smaller number of compounds.

### The novel MC-specific descriptors add non-redundant information about MC properties

**Fig. 2:**
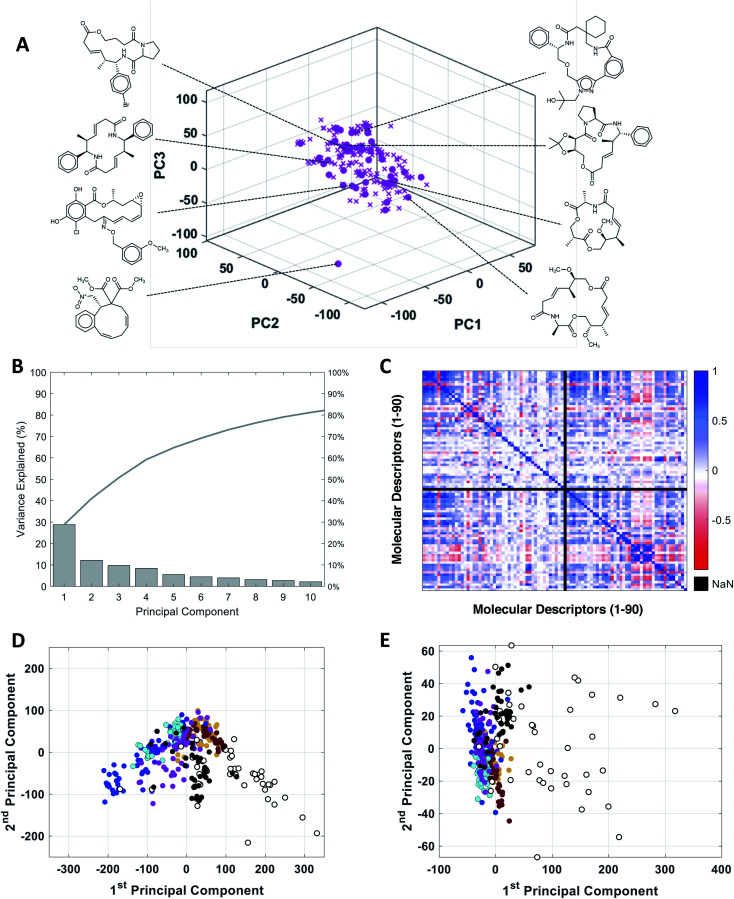
PCA analysis of the combined MC compound sets. (A) The BU-CMD set (Set A, 106 compounds, 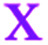) plotted in Principal Component space, showing that the 42 representative compounds (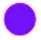) selected using *k*-medoids clustering (*k* = 42) comprise a diverse subset that effectively covers the property space of the full Set A. (B) Percentage of variance explained by each PC in PCA of the combined MC sets. (C) Heat map showing the covariance between the 90 MolDs, across all MC sets, in the form of the Pearson Correlation Coefficient (PCC); PCC = 1 indicates two properties are exactly proportional to each other, PCC = 0 indicates they are uncorrelated, and PCC = −1 indicates they are perfectly anticorrelated. Property 49, peripheral S/peripheral HA, returned “Not a Number” (NaN), because there are no sulfur atoms in peripheral positions in any of the compounds included in the analysis. (D) Plot of compound scores in PC1 *versus* PC2, showing how the compounds are distributed in MC chemical space with respect to the first two PCs when the PCA is performed using all 90 MolDs, compared to (E) the same analysis using just the 6 MolDs included among the classic descriptors contained in Lipinski's rule of five (Ro5)^4^ and Veber's rules.^39^ Compounds in panels D and E are colored as follows: Set A (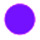), Set B (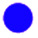), Set C (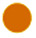), Set D (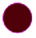), Set E (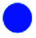), Set F (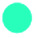), oral MC drugs and clinical candidates (●), and non-oral MC drugs and clinical candidates (○).

To assess the utility of our MC-specific MolDs in describing MC chemical space, we used the machine learning technique of Principal Component Analysis (PCA).^50,51^ PCA is a simple, deterministic statistical analysis method that gives results that are fully interpretable. For the analysis we assembled a collection of MCs comprising several distinct compound sets. One set comprised 42 oral MC drugs and clinical candidates (“Oral MC Drugs”) we were able to cull from various sources; another contained 52 non-oral MC drugs and clinical candidates (“Non-oral MC Drugs”). The oral MC drugs included macrolide antibiotics, ansamycins, immunomodulatory and related macrolides, peptidometic HCV protease inhibitors, kinase inhibitors, and three large and structurally complex cyclic peptides. The non-oral MC drugs also included macrolides, ansamycins, and other complex natural products, as well as ∼30 cyclic peptides. We also included six sets of synthetic MCs, comprising a set from the Boston University Center for Molecular Discovery (BU-CMD) compound library (Set A), plus several large (>1000 compounds) MC collections available from different commercial vendors (Sets B–F). Some of these compound sets contained a small proportion (∼2% overall) of smaller-ring compounds, with ring-sizes of ten. All the synthetic MCs included in the analysis are physical compounds that at the time of analysis could be purchased or acquired. To avoid biasing the analysis, we selected an equal number of representative compounds from each set, using *k*-medoids clustering^52,53^ with respect to all 90 MolDs (Fig. 2A). We also scaled each of the 90 MolDs to avoid the PCA being dominated by the descriptors with the largest numerical values.

The representative MC sets were analyzed by PCA. Fig. 2B shows that inclusion of ten PCs is required to explain >80% of the variance in the original data set. This outcome contrasts with the results of PCA done using only the six Lipinski^4^ and Veber^40^ properties, for which almost 100% of the variance among the same compounds is captured in just the first two PCs, consistent with the high covariance that exists between these properties for our MC sets (Fig. S1†). Thus, inclusion of the additional 84 MolDs captures a substantial amount of additional and non-redundant information about the MCs. The increased discriminatory power provided by our full descriptor set is illustrated by plotting the property values for each compound on axes of PC1 *versus* PC2. Compound scores from PCA done using only the 6 MolDs of Lipinski and Veber, plotted in 2D space, shows all of the compounds except the non-oral MC drug set to be clustered together, whereas the scores from the PCA using all 90 MolDs are much more widely distributed (Fig. 2D and E), providing significantly greater discrimination of the synthetic MC sets from each other and from the oral MC drugs (Fig. S2, Supplemental Video A†).

### Druglike macrocycle property space and its coverage by synthetic MC chemotypes

The observation that, when using the 90 descriptors, multiple PCs are required to capture the bulk of the variance between the compounds, shows that the MC sets are differentiated by multiple distinct and mutually orthogonal characteristics. PCs 1 and 2 together explained 42% of the variance contained in the original 90-dimensional property matrix, and PCs 1–3 together explained 51% of the variance. Thus, plotting the compound scores in the first two or three PC dimensions provide meaningful visualizations of the chemical space defined by the compounds (Fig. S2B†).

**Fig. 3:**
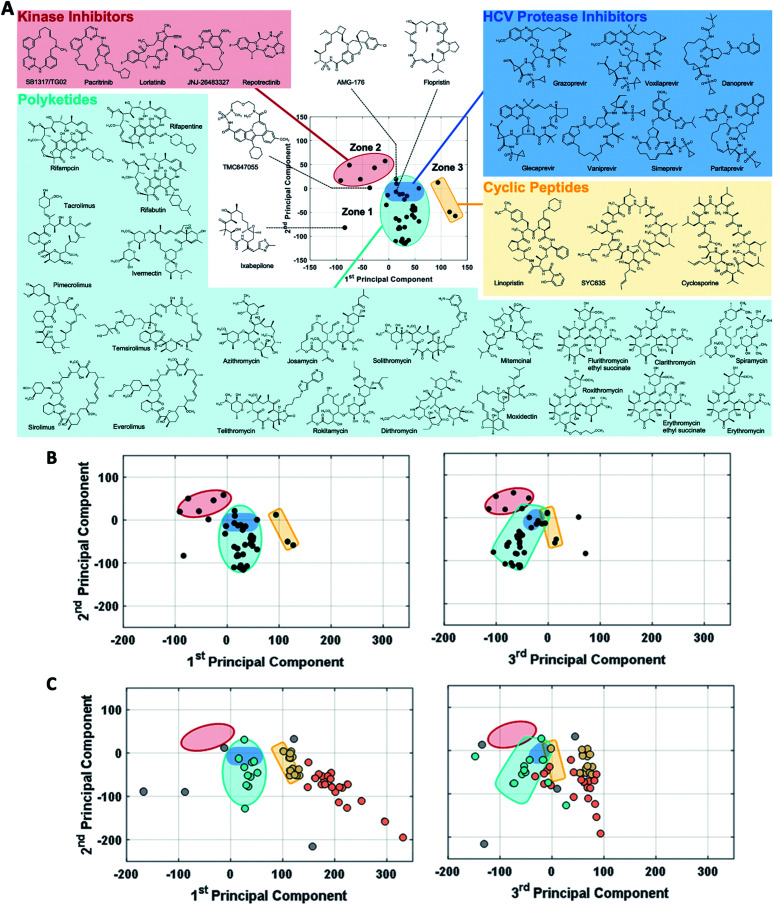
MC drug-like chemical space as defined by PCA. (A) The oral MC drugs and clinical candidates mostly fall into three neighboring zones of structure–property space. Zone 1 (turquoise) is occupied by polyketides, which include macrolide antibiotics, ansamycins, immunomodulatory and related macrolides, as well as by the peptidometic HCV protease inhibitors (shaded in dark blue). Zone 2 (red) is occupied by ATP-site binding kinase inhibitors. Zone 3 (yellow) contains large and structurally complex cyclic peptides. (B) Viewing the oral MC drug set from the perspectives of PC1 *vs.* PC2 (left) and PC2 *vs.* PC3 (right) shows that these three zones are well-defined in 3-dimenisional PC space, which is sufficient to capture >50% of the variance in properties between the compounds. Two compounds that fall outside the delineated zones are TMC647055, which appears close to Zone 2 with respect to PCs 1 and 2 but which is clearly far away from this locus when PC3 is considered, and ixabepilone, which is far from any Zone in both PCs 1 and 3. (C) The coordinates of the non-oral MC drugs and clinical candidates, plotted with respect to PCs 1 and 2, with the Zones identified in (B) superimposed. In the left panel, the data points are colored according to which of the oral MC drug zones they appear to overlap with in PCs 1 and 2: turquoise for Zone 1 and yellow for Zone 3. Compounds that fall into Zone 3A (see main text) are colored orange. Four additional non-oral compounds, plerixafor, latrunculin B, tubocurarine, and sugammadex (colored grey), fall outside Zones 1–3 with respect to both PC1 and PC3, and are considered singletons. The right panel shows the locations of the non-oral MC drugs with respect to PCs 1 and 3, retaining the color-coding from the left panel. Note: all 52 non-oral MC drugs and clinical candidates are plotted in (C).

In analyzing such plots for the oral MC drug set, we found that these compounds clustered by chemotype, which in some cases correlated with their pharmacological function. Fig. 3A shows the 42 oral MC drugs and clinical candidates plotted in 2-dimensional PC space. Of these, 31 cluster in a relatively small and well-defined region of MC property space close to the origin, which we designate as Zone 1. Of the 11 remaining oral drugs, eight reside in two adjacent regions, one defined by a handful of 13- to 18-membered MC kinase inhibitors with multiple fused aromatic rings, typified by the Jak2 inhibitor pacritinib (Zone 2), and the other containing the three large and densely substituted cyclic peptides exemplified by cyclosporine A (Zone 3). Viewing the compounds with respect to their scores in PC3 confirms that these zones are compact in three-dimensional PC space (Fig. 3B, S3A, B and Supplemental Video B†).

Turning to the non-oral MC drugs, some also reside in or near Zone 1 (Fig. 3C). This observation indicates that occupancy of this region of chemical space is compatible with oral bioavailability but does not guarantee it. None of the non-oral MC drugs occupy Zone 2. Instead, the majority occupy a region that runs parallel to Zone 3 (in Fig. 3C most clearly seen in the plot of PC1 *vs.* PC3), extending to encompass an elongated portion of chemical space, which we term Zone 3A (Fig. 3C, S3C, D and Supplemental Video C†). Only one non-oral compound, quinupristin, actually resides in Zone 3 when considered in three PC dimensions, with a second, thiostrepton, lying nearby. Among both the oral and the non-oral compounds, there are a small number that do not fall into any of the above-mentioned zones (Fig. 3B and C).

**Fig. 4:**
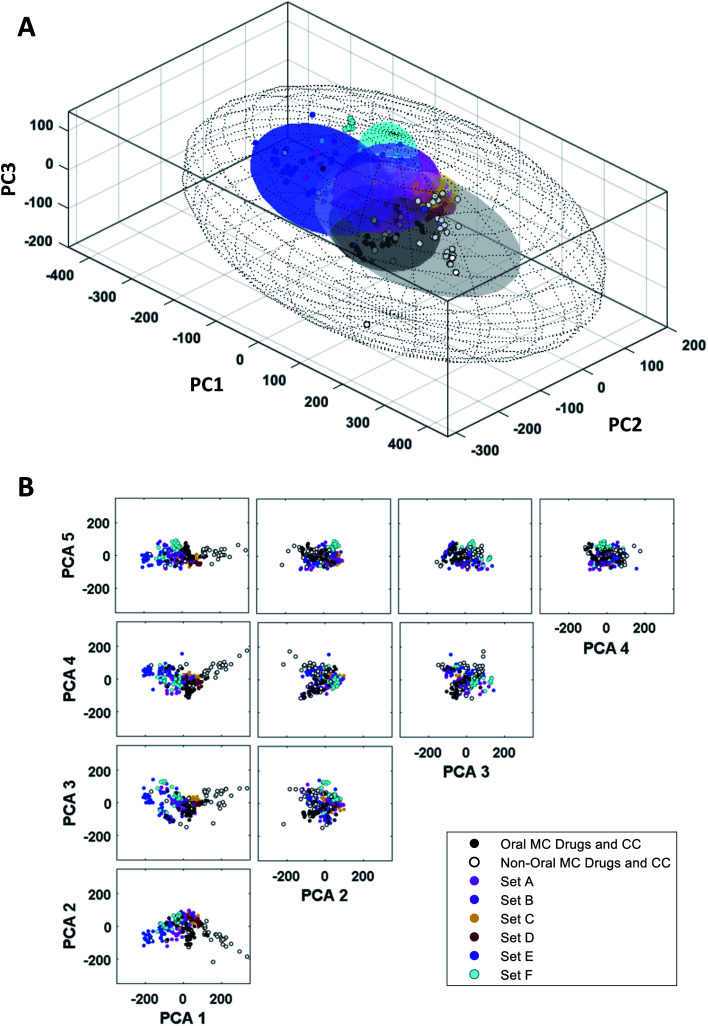
“MC Chemical Space” as defined by PCA of all compound sets using all 90 MolDs. (A) The coordinates (scores) of the representative compounds from each MC set plotted with respect to PCs 1–3. The regions of structure–property space occupied by each MC set are represented as ellipsoids that encompasses the middle 95% of compounds with respect to each PC axis. The outer, wireframe ellipsoid encompasses 100% of the compounds from all sets. Supplemental Video A† shows the rotation of panel A. (B) The distribution of the MC sets in chemical space with respect to the first five PCs, which collectively capture >65% of the total variance between the compounds.

The synthetic MC sets A–F, when plotted in three-dimensional PC space (Fig. S2B†), or higher dimensions (Fig. 4B), occupy distinct but partially overlapping regions. Several of these sets show good overlap with oral MC drug Zone 2, but there is little overlap of any set with Zone 1, and essentially none with Zone 3 (Fig. 4A and S4†). Thus, the synthetic MCs achieve only relatively poor sampling of the regions of property space where the bulk of the known MC drugs and clinical candidates reside.

### Quantifying the diversity of the synthetic MC sets and their similarity to the oral MC drug set

We assessed the structural and physicochemical diversity of the MC sets by quantifying the volume of PCA chemical space occupied by each, using the scores from PCs 1–10 to capture >80% of the variance in the compound properties. To do this, for each set we defined a 10-dimensional (hyper)ellipsoid that encompassed the middle 95% of compounds with respect to each PC axis (Fig. 4A and Supplemental Video A†). For the purposes of this analysis we considered the oral MC drugs to comprise a contiguous cluster, ignoring the fine structure among these compounds discussed above, and treated the non-oral MC drugs similarly. We also calculated the hyperellipsoid that encompassed 100% of the representative compounds from all MC sets (Table 1). Because conceptualizing the significance of volume differences in 10-dimensional space is challenging, and the absolute numerical values depend on the number of dimensions, we also give the average radius with respect to a single PC axis, equivalent to the 10^th^ root of each hyperellipsoid volume. Of the synthetic MC collections, Set E occupies the largest volume. Set A was also quite diverse, while Sets B, C, F, and particularly Set D sample significantly smaller regions of MC chemical space. We note that, within their respective volumes, the density of compounds is quite uneven for most MC sets. Table 1 additionally shows that even the most diverse MC set occupies ≪1% of the total MC chemical space. This finding suggests that large regions of MC property space are not well-covered by synthetic MC collections of the types included here, though whether devising compounds to sample this vacant space is synthetically feasible or even in all cases desirable is unclear.

**Table 1:** Diversity of compound sets and proximity to oral MC drug Set

	Hyper-ellipsoid volume^a^ (×10^−15^)	Hyper-ellipsoid average radius^b^	Extent of MC chemical space covered	Euclidean distance in 10-D PC space^c^ (normalized)
All compounds	106 000 000 000	400	(100%)	n/a
Oral MC drugs/CC	1300	65	0.000001%	(0)
Non-oral MC drugs/CC	160 000	105	0.000151%	0.322
Set A	3600	72	0.000003%	0.202
Set B	200	54	0.000000%	0.275
Set C	22	43	0.000000%	0.272
Set D	8	39	0.000000%	0.270
Set E	50 000	93	0.000047%	0.387
Set F	70	48	0.000000%	0.352

a Volume of a 10-dimensional hyper-ellipsoid encompassing the middle 95% of compounds in each of PCs 1–10, calculated according to eqn (5).

b 10th root of the hyper-ellipsoid volume, corresponding to the average radius (semi-axis) along one PC axis. Units correspond to the distance units of the PC axes.

c Distance of the center of each compound set from the center of the oral MC drug set, expressed as a fraction of the radius in PC1 of the “MC universe” defined by all of the compounds.

As a measure of the degree to which each synthetic MC set resembles the oral MC drugs, we calculated the Euclidean distance in 10-dimensional PC space separating the center of mass of each set from that of the oral MC drug set. To aid interpretation, we normalized these distances to the size of the MC chemical space “universe”, by expressing each as a fraction of the longest dimension of the ellipsoid that contains all compounds in the study (434 units, Table 1). The smaller the distance, the more similar a given MC set is, on average, to the oral MC drugs, with respect to the 90 MolDs used in the analysis. The results (Table 1) show that none of the compound sets is centered on a point that is closer than 0.20 units or further than 0.39 units from the center of mass of the oral MC drug set, with Set A being closest and Set E being furthest away. These distances from MC oral drug space are somewhat larger than the average radii of the ellipsoids that define the volume occupied by each MC set (Table 1), consistent with the visual observation from Fig. 2D that the degree of overlap of the synthetic MC sets with the oral MC drugs set generally is small.

### Identifying the molecular characteristics that define the major axes of MC chemical space

To use the PCA results to understand which structural and physicochemical features of the compounds are most important for distinguishing one set from another, or to inform the design of new MCs that more closely resemble known oral MC drugs, it is necessary to translate the highest ranked PCs back into chemically interpretable properties. This can be done by examining which of the original MolDs have the highest coefficients in each PC, and then identifying the underlying theme that connects these properties. We considered a MolD to be an important contributor to a PC if its coefficient had a magnitude greater than an arbitrary threshold of 0.16. To identify the themes that connect the different MolDs that score highly in a particular PC, we examined the covariance between the major contributing MolDs for each PC, using the absolute values of the correlation coefficient to capture both positive correlation and negative (anti-) correlation. This covariance identified clusters of MolDs that presumably reflect a common aspect of the property captured by that PC. Typically, a given PC returned 2–3 such MolD clusters which, by definition, must all contribute in some way to a common overarching structural or physicochemical feature represented by the PC. Identifying the unifying characteristics within and between these MolD clusters allowed us to deduce the molecular characteristics that drive each PC.

**Fig. 5:**
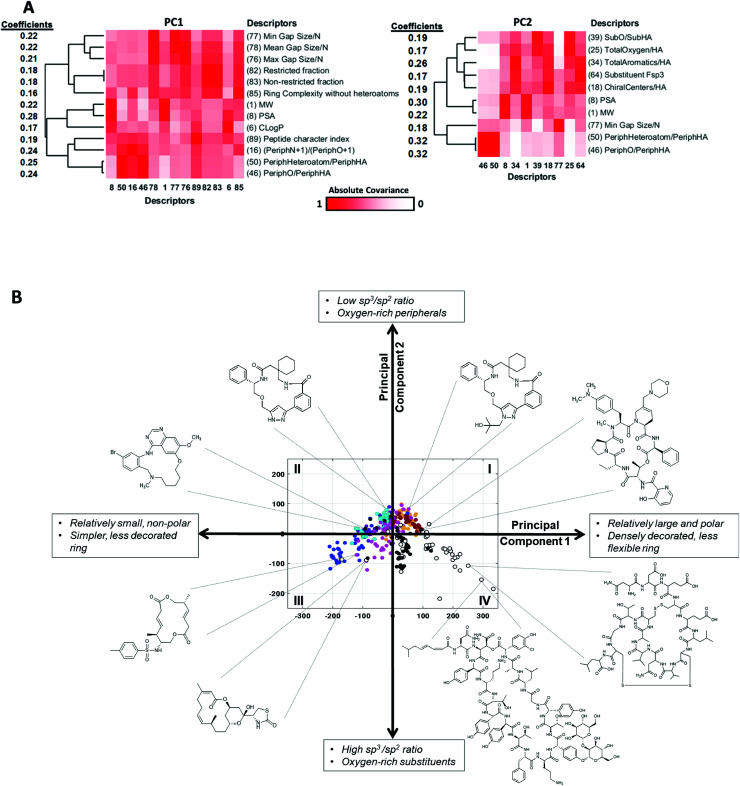
Property themes underlying PCs 1 and 2. (A) The descriptors with the largest (absolute value) coefficients in PC1 (left) and PC2 (right), clustered with respect to the absolute values of the covariance (Table S4†), so that MolDs that contribute related information cluster together. The heat maps are colored according to the absolute value of the correlation coefficient between each pair of MolDs. The coefficients for each MolD are shown to the left of each heat map, to show the magnitude and sign of the contribution each MolD makes to the PC. (B) The compounds representing each MC set plotted with respect to the first two PCs, with selected compounds highlighted to illustrate the themes that define the first two PCs. The quadrants are numbered as described in the main text.

The MolDs with the highest coefficients in PC1 are shown in Fig. 5A. They cluster into two main groups, one containing mostly descriptors relating to the number and polarity of the peripheral groups attached to the ring (MolDs 16, 46, 50, 89), as well as overall molecular size and polarity (1, 6, 8), and the second primarily describing the rigidity of the MC ring (82, 83) and how densely the ring is decorated with substituents (76, 77, 78) and peripheral groups (85). There are subtleties to this interpretation, however. Descriptors quantifying how many ring atoms separate the substituents (“Gap Size”) that are normalized by dividing by number of atoms in the ring, *N*, *i.e.* MaxGapSize/*N* (76) MinGapSize/*N* (77), and MeanGapSize/*N* (78), were more highly ranked in PC1 than the MolDs containing the corresponding un-normalized values, indicating that the differentiating factor is not how many substituents or peripheral groups are present but how densely the ring is decorated on a per ring atom basis. Furthermore, ring complexity (85), which treats substituent atoms and peripheral groups as equivalent, also appears among the top-ranked descriptors. The directionality of the influence of each descriptor, inferred from the sign of its coefficient (Fig. 5A), indicates that high scores in PC1 are associated with compounds that are large, have a high polar surface area, a ring that is densely decorated with substituents and peripheral groups, and contain features that rigidify the ring such as amide bonds and fused rings, both with and without bridges.

We applied the same process to interpret the theme underlying variations in scores with respect to PC2. We note that some of the descriptors that contribute strongly to PC2 were also important in PC1. However, by the nature of PCA, the variance captured by each PC comprises an orthogonal component of the data, and therefore the aspect of a given MolD that causes it to contribute to one PC must be distinct from that causing it to contribute to a different PC. For example, the number of sp^2^ centers in the MC ring will affect the shape of the molecule, but also its flexibility, its polarizability, and other properties, and each different aspect of the property may be reflected in a different PC. Based on the most influential descriptors, PC2 reflects the ratio of sp^2^- to sp^3^-hybridized carbons, and the proportions of peripheral and substituent atoms that are oxygens (Fig. 5A). Specifically, a compound with a high score in PC2 will have a high proportion of sp^2^ carbons, a high proportion of peripheral atoms that are oxygens, but substituents with a relatively low oxygen content.

These thematic interpretations of PC1 and PC2 are corroborated by inspection of how individual compound structures distribute on a plot of PC1 *versus* PC2. Fig. 5B shows that the chemical space defined by these two PCs can be divided into quadrants. The macrocycles in Quadrant I are relatively large and polar, densely decorated with substituents and peripheral groups, with a high sp^2^ content, a high fraction of peripheral atoms that are oxygens, and have relatively rigid rings. Quadrant II includes compounds that are smaller and less polar, with less decorated rings, also with high sp^2^ content and a high proportion of peripheral oxygens. The compounds in Quadrant III are similarly simple in structure, but with a higher sp^3^ content, a proportionately higher content of oxygens in the substituents, and a balance of oxygens and methyl groups in peripheral positions. Macrocycles in Quadrant IV are substantially larger and more polar, with densely decorated and relatively rigid rings, with substituents having a high sp^3^ content and a high proportion of oxygens, and with a substantial fraction of nonpolar peripheral atoms. The oral MC drugs are predominantly located in Quadrant IV and the proximal regions of Quadrant 1, except for the Zone 2 kinase inhibitors that lie close to the origin but in Quadrant II.

### Navigating MC chemical space to design more druglike compounds

The combination of molecular features that allows certain high MW MCs to have good druglike properties is the topic of considerable current research, and a few general ideas are beginning to emerge.^2,8,11,13,18,19,44,48,54,55^ However, we are still far from being able to rationally design druglike MCs from first principles. An alternative, empirical approach to designing MC libraries for drug discovery is to try to design compounds with structural and physicochemical features that resemble those of known MC drugs. The development of new descriptors that capture additional facets of MC structure and properties, described here, enables such an approach to be undertaken in a more comprehensive and nuanced way than has hitherto been possible.

**Fig. 6:**
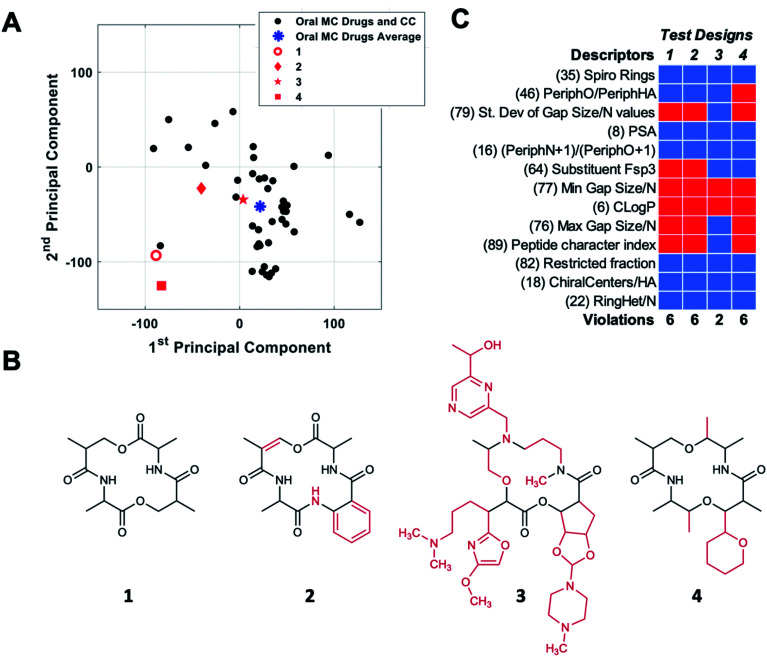
Navigating MC chemical space using the themes from PCs 1 and 2. (A) Location of starting compound **1** (red circle) on a plot of PC1 *versus* PC2. The oral MC drugs and clinical candidates are shown as black circles, and their center of mass is indicated by a blue asterisk. The locations of the new compound designs, derived by redesigning **1** according to the structural themes captured by PCs 1 and 2, are shown as solid red symbols. (B) New compound designs **2** and **3,** arrived at by redesigning **1** to increase the compound's score in both PCs 1 and 2, as described in the text. Compound **4** was designed without using the PC themes. The features of each structure that are different from **1** are shown in red. (C) Assessment of designs **1–4** with respect to the 13 properties identified as most important by PCA (*vide infra*).

The themes derived from PCA can be used to aid MC design by providing guidance for how a chemotype that maps to a given location in MC chemical space might be redesigned to move toward a target location. To test how these themes might be implemented in MC design, we started with compound CMLD000944 (**1**), a prototypical representative of a relatively simple MC chemotype from our internal BU-CMD collection (Set A). Compound **1** lies in Quadrant III as defined in Fig. 5B, far from the center of mass of the oral MC drug space (Fig. 6A). We explored how the themes from PC1 and PC2 might be used to design more elaborate MCs, loosely based on the 14-membered ring exemplified in **1**, that occupy positions closer to the center of mass of the oral MC drug set. To do so requires devising compounds that have a substantially increased score in PC1 and a modestly increased score in PC2, compared to the values calculated for **1**. According to the themes identified in Fig. 5B, to increase the score in PC1 requires making the structure larger and more polar and increasing the density of decoration and rigidity of the ring. We therefore modified the structure of compound **1** by adding a substituent to increase the density of decoration of the ring, and by introducing a π-bond and an amide bond in the ring. Increasing the score in PC2 requires increasing the proportion sp^2^ hybridized carbon atoms and increasing the proportion of peripheral atoms that are oxygens while minimizing the number of substituent oxygens. To achieve the small increase in PC2 we were seeking we made the substituent aromatic, with no oxygens, and slightly increased the proportion of peripheral oxygens by eliminating one peripheral methyl group (replaced by the substituent). The scores for the resulting compound design, **2**, were determined by calculating the values of the 90 MolDs for the new structure, and then using the coefficients from the original PCA analysis to transform these values into PC space, as described in Methods. Fig. 6A shows that these structural changes did indeed increase the scores in both PCs, but fell short of the target location with respect to PC1 while overshooting with respect to PC2, resulting in a distance of 197 (unnormalized) units from the target location. To identify a design nearer the desired destination in PC space we devised a new structure, starting again from **1**, with substantially increased MW and tPSA, and with three large substituents to increase the density of decoration of the ring, all intended to further increase the score in PC1. At the same time, to attenuate the increase in PC2 we eliminated two peripheral oxygens, and made sure there was a balance of sp^3^*versus* sp^2^ carbons, plus a few chiral centers and some oxygen atoms, in the substituents. These structural changes were successful in moving the resulting design, **3**, to a position close to the target destination on the plot of PC1 *vs.* PC2 (Fig. 6A).

Importantly, not all redesigns that might superficially appear to make a chemotype more similar to the oral MC drugs will move the structure closer to the intended region of chemical space. For example, guided simply by the idea that natural product macrocycles tend to have relatively complex structures with a high proportion of peripheral methyl groups^2^ and chiral centers,^56^ we changed the structure of **1** to a design that contains a substituent, has two fewer peripheral oxygens, two more peripheral methyls, and eight chiral centers instead of four. Fig. 6A shows that these changes, embodied in design **4**, did not move the compound appreciably closer to the center of oral MC druglike space with respect to PCs 1 and 2.

To quantify how effective these designed changes were in moving from the starting structure **1** towards the target location, we calculated the distance of each design from the center of oral MC drug space in 10 PC dimensions. The results show that starting compound **1** is 227 units from the center of the oral MC drugs set in 10-dimensional PC space. Compound designs **2** and **3** were substantially closer to the target location, having distances of 102 and 78, respectively. Examination of the movement of the design in PCs 3–5 shows that, in each case, compounds **2** and **3** were closer to the center of the oral MC drugs than the starting compound (Table S5†). While exemplified above for a single starting structure, consideration of the PCA themes could equally be applied to an entire library, to identify overall changes in properties that would result in more MC-druglike chemotypes. A similar approach could presumably be taken to navigate to other target locations in MC chemical space.

### Structural and physicochemical properties of oral MC drugs

Our PCA analysis indicates that MC chemical space is complex, with high dimensionality required to capture the variance between structures. The PCA themes provide some intuitive guidance as to how to redesign a given synthetic MC library to achieve chemotypes that are more MC drug-like. However, it would clearly be useful to have design guidelines that are more easily actionable. To this end, we tested whether it is possible to identify a region of the original MC property space that maps onto the oral MC drug-like region of PC space. We identified the 20 MolDs that were most influential for PCs 1–10, clustered them to eliminate any that were highly redundant, and for the 13 MolDs that remained we calculated the property ranges that encompass 80% of the oral MC drug set (Table 2).

**Table 2:** The 13 properties identified by PCA as being most important for distinguishing the compound sites, and the value ranges observed for the oral MC drug set

Descriptor	Importance (PCs 1–10)	Distribution^a^	Range encompassing 80% of oral MC drug set
(35) Spiro rings	577	n/a	0
(46) PeriphO/PeriphHA	567	A	0.30–0.67
(79) St. dev of gap size/*N* values	309	A	0.06–0.26
(8) tPSA	274	A	50–230
(16) (PeriphN + 1)/(PeriphO + 1)	252	B	0.13–0.50
(64) Substituent Fsp3	248	B	0.22–1.00
(77) Min gap size/N	228	B	0–0.13
(6) CLogP	164	A	2.40–6.00
(76) Max gap size/N	149	C	0.24–0.64
(89) Peptide character index	120	B	0–0.44
(82) Restricted fraction	111	C	0–0.42
(18) ChiralCenters/HA	81	A	0.02–0.33
(22) RingHet/*N*	71	B	0.06–0.31

a A, normal; B, unimodal but asymmetric; C, bimodal or multimodal.

**Fig. 7:**
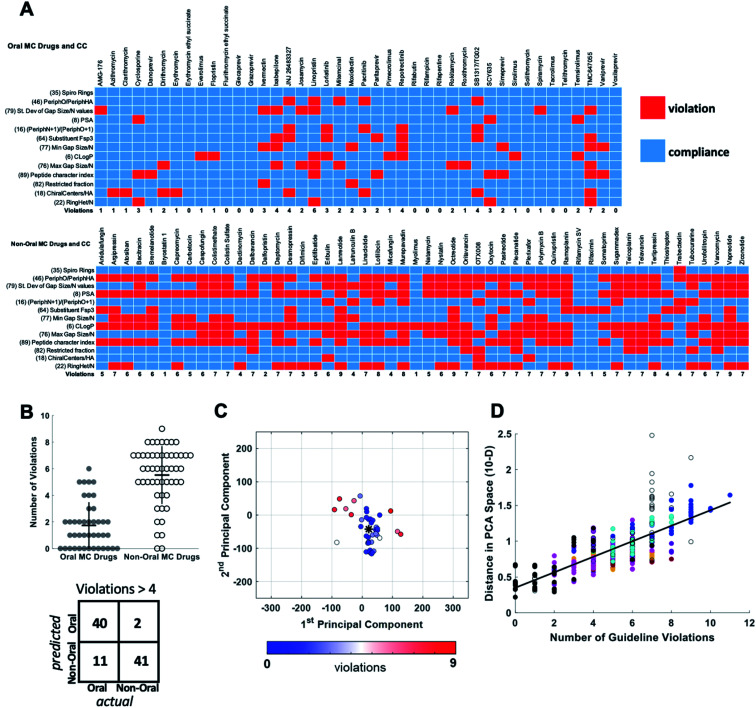
Violation of key property value ranges correlates with distance from oral MC drug space. (A) Properties of the 42 oral MC drugs and clinical candidates (top) and the 52 non-oral MC drugs and clinical candidates (bottom), color-coded to indicate whether the value for each property falls within (blue) or outside (red) the ranges defined in Table 2. (B) Beeswarm plot^63^ showing the distributions, means (horizontal lines), and interquartile ranges (vertical lines), of the number of violations from (A) for the oral (filled circles) and non-oral (open circles) MC drugs and clinical candidates. The truth table, for a threshold of ≤4 *versus* >4 violations, is shown below. (C) The oral MC drugs and clinical candidates plotted with respect to PCs 1 and 2, color-coded with respect to the number of violations from (A), showing that compounds with more violations lie further from the center of oral MC drug space (*). (D) The distance of each compound, in 10-dimensional PC space, from the center of the oral MC drug set, plotted against the number of property violations, for all MC sets. Compound sets are colored as in Fig. 2, 4 and 5. The distance is expressed in the units of PC space, normalized by dividing by the range of scores seen across all compounds in PC1.

For each of the oral and non-oral MC drugs and clinical candidate compounds we determined how many of the 13 descriptors have values that fall within *versus* outside these “Oral MC Drug-like” property ranges (Fig. 7A). The 42 oral MC drugs and clinical candidates have an average of 1.7 out of 13 MolDs with values that fall outside the target ranges. Only 2/42 (∼5%) of the oral MC drugs had >4 out-of-range property values. In contrast, for the non-oral MC drugs and clinical candidates the proportion having >4 violations was 41/52 (79%) (Fig. 7B). Thus, the number of top 13 MolDs that fall outside the identified ranges provides a degree of discrimination between the oral and non-oral MC compounds. Results for each MC set are shown in Supplementary Fig. S8.† To further test the number of violations as a surrogate for proximity to oral MC drug space, we calculated the distance in 10-dimensional PC space of each compound from the center of the oral MC drugs set, and examined how well this distance correlated with the number of violations. The results show that compounds with ≤4 violations do indeed reside close to the core of oral MC drug space, and the more violations a compound has, the further from this region the compound lies in PC space (Fig. 7C and D). This result is robust to whether the analysis includes all 10 PCs or only 7. These findings suggest that evaluating compound designs with respect to the number of violations of the 13 property ranges identified in Table 2 represents an effective surrogate for estimating how close the compound lies to oral MC drug space.

### Analysis of historical MC optimization efforts

**Fig. 8:**
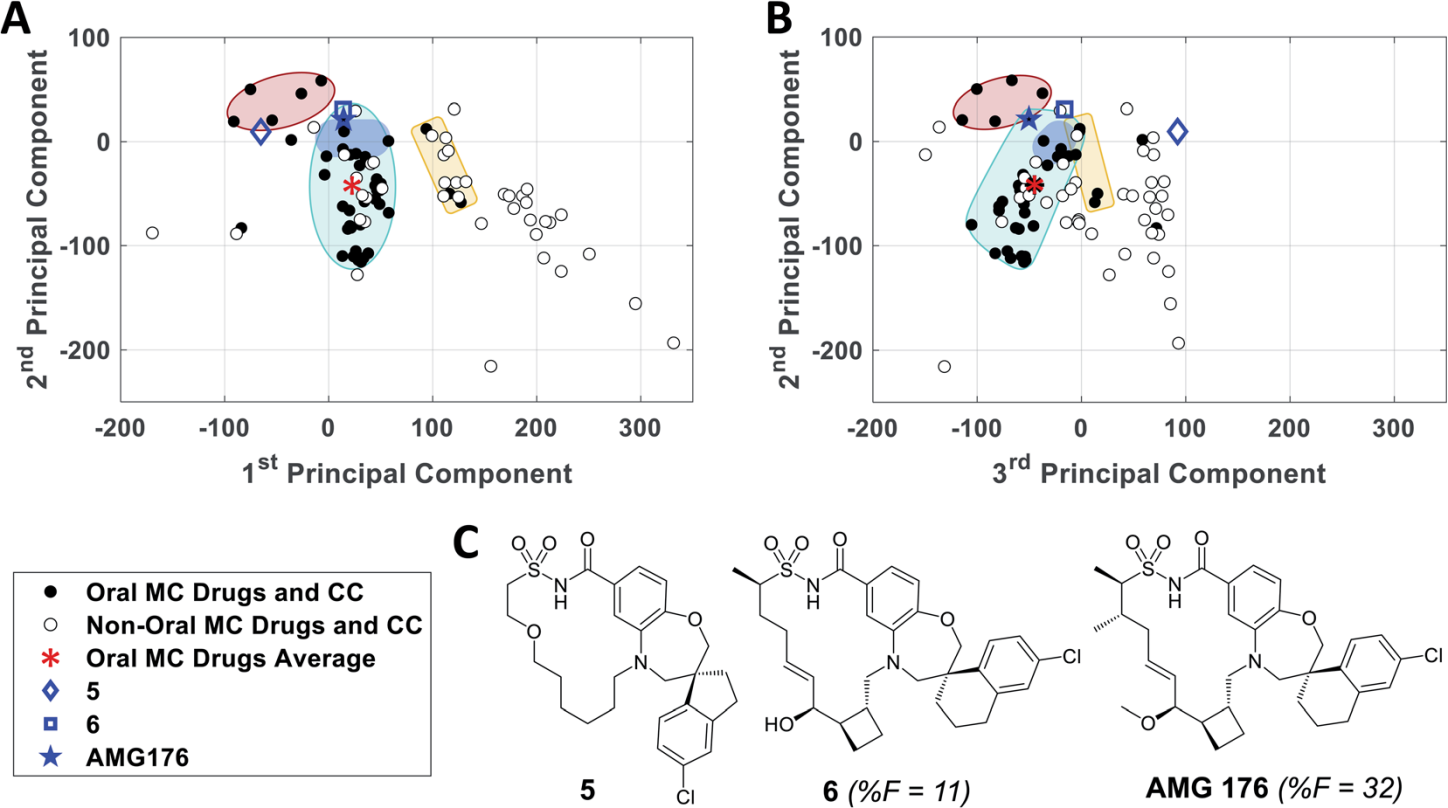
Analysis of the historical optimization of AMG176 with respect to the locations of key compounds in MC chemical space. Selected compounds from the work of Caenepeel *et al.*,^57^ which led to clinical candidate AMG 176, plotted with respect to (A) PC1 *versus* PC2 and (B) PC2 *versus* PC3, overlaid with Zones 1–3 from Fig. 3. C. Structures of AMG176 and the discussed precursors.

To further validate the analysis described above, we examined published examples of MC optimization to see whether improvements in oral bioavailability (OBA) were associated with movement from outside to inside the oral MC druglike zones. Fig. 8 shows that, in the discovery of the oral MC clinical candidate AMG176, the early compound **5** lies well outside Zone 1 with respect to both PC1 and PC3. Addition of rigidifying features to give **6** resulted in a compound lying in Zone 1 which showed 11% OBA in mouse. Methylation of a hydroxyl group and addition of a second peripheral methyl group resulted in the final compound, AMG176, which also resides in Zone 1, with respect to PCs 1, 2 and 3, and has OBA = 32% in mouse and 70% in cynomolgous monkeys.^57^ These structural modifications were associated with a reduction in the number of violations of the 13 key properties from Fig. 7, from 6 violations for **5** to 2 violations for AMG176. Another example is the cyclic peptide-peptoid hybrid CXCR7 modulator reported by Boehm *et al.*, for which a modest movement towards Zone 3 was associated with achievement of 18% oral bioavailability in rat^58,59^ (Supplementary Fig. S10A†). In contrast, for two other historical examples of MC optimization, the NS3 protease inhibitor BILN 2061^60,61^ and the Sanglifehrin A-derived cyclophilin inhibitor of Steadman, Mackman *et al.*,^62,63^ the starting compounds already resided in or near Zone 1, and the structural changes that conferred oral bioavailability did not substantially change the compounds' positions in MC chemical space (Supplementary Fig. S10B and C†) or materially alter the number of property violations. These observations reinforce the notion that proximity to the oral MC drug zones is associated with improved prospects for oral bioavailability, but that residence within these zones does not by itself guarantee this outcome.

## Discussion

The MC-specific molecular descriptors introduced in this study capture features of MC structure and properties beyond those described by existing descriptors, and therefore enable a more nuanced discrimination between different MC chemotypes. Notably, nine of the 13 descriptors that were found to be most important in PCA over 10 dimensions were from among the new, MC-specific subset. The new properties that proved most important in distinguishing the MC compound sets include the identity and polarity of atoms in peripheral positions, the proportion of heteroatoms in the ring, how densely the ring is decorated with substituents and how evenly these substituents are distributed around the ring, the sp^3^*versus* sp^2^ carbon content of the substituents, and the flexibility of the MC ring. In contrast, some others of the new MC-specific descriptors did not contribute as much to distinguishing the chemotypes. It remains to be determined which of the new descriptors reflect properties directly relevant to the pharmacological utility of MCs. Nonetheless, the observation that their use helped distinguish oral MC drugs and clinical candidates from synthetic MC collections of unknown bioavailability suggests that at least some of these properties could be important to good druglike behavior of MCs. Our results, therefore, identify new properties to be considered when attempting to design synthetic MC libraries for use in drug discovery.

Many of the oral MC drugs and clinical candidates were found to fall into a region of MC property space that we termed Zone 1. Most of these compounds reflect variations on just a few chemical themes. Specifically, the 23 oral MC drugs that are polyketides comprise 13 erythromycin derivatives, five rapamycin analogues with either 29- or 21-membered rings, four ansamycins, and an avermectin. Zone 1 additionally accommodates 7 peptidomimetic HCV protease inhibitors, a streptogramin, and the unique synthetic macrocycle AMG176. Nonetheless, the observation that 7 distinct chemotypes occupy this relatively compact region in chemical space supports the hypothesis that there are well-defined combinations of molecular properties that are most compatible with oral bioavailability of macrocyclic compounds, and that the descriptors used in the current study are useful in identifying these properties. The results additionally establish that there are convergent solutions to devising macrocyclic compounds that occupy this space. Occupancy of Zone 1 is not essential for MC oral bioavailability, as shown by the oral compounds that define Zones 2 and 3. But our results suggest that a combination of molecular features that places a compound in this location conveys some probability of good pharmaceutical properties. It is unclear whether Zones 1–3 represent three convergent ways of balancing structural and physicochemical properties to achieve oral bioavailability. Some of the structural distinctions we observe could be irrelevant to bioavailability, in which case the three zones could be equivalent ways to achieve oral absorption. While Zones 1–3 represent demonstrably fruitful regions of MC property space for oral drug discovery, they are not necessarily the only such regions. Indeed, the location in MC chemical space of the oral MC drugs ixabepilone, an epithilone,^64^ and TMC637055, a synthetic inhibitor of HCV NS5B polymerase,^65^ proves that there are regions outside Zones 1–3 that are compatible with oral bioavailability.

Comparison of the oral drugs with the non-oral MC drugs and clinical candidates was also informative. The majority of non-oral drugs lie in a region of chemical space, which we termed Zone 3A, outside of the three oral drug zones. However, a number of non-oral drugs and clinical candidates reside in Zone 1, and one occupies Zone 3. This finding shows that combinations of properties that position compounds in these regions of chemical space can be compatible with oral bioavailability but are not sufficient to ensure it. Which molecules in Zones 1 and 3 are orally available and which are not evidently depends upon molecular features that are not captured in our descriptor set, possibly including the ability to undergo chameleonic conformational change or other very specific conformational behaviors, as is known to be the case for the Zone 3 drug cyclosporine.^44,45,49,52^

A notable trend in Fig. 3A is that the MC drugs that are largely synthetic in origin occupy a different area in MC property compared to those that are natural products or their close derivatives. The synthetic compounds, which comprise the synthetic kinase inhibitors, the HCV polymerase inhibitor TMC647055,^65^ and Amgen's mcl-1 inhibitor AMG176,^57^ appear toward the upper left in Fig. 3A. In contrast, the compounds that are natural products or derivatives occupy the lower and right-hand regions. The peptidomimetic HCV protease inhibitors, which result from the combined efforts of nature and chemist, occupy a boundary sector between these areas. Consideration of the trends we observed in PC space suggests that the area occupied by the synthetic compounds corresponds to the “flatland” of structure space that Lovering *et al.* described as being characteristic of many chemotypes derived from medicinal chemistry.^56^ Specifically, compared to the natural product-derived drugs, the synthetic compounds tend to have higher scores in PC1 and lower scores in PC2. Thus, the synthetic drugs tend to be smaller and less polar, with simpler, more flexible rings, a higher proportion of aromatic and other sp^2^-hybridized carbons, fewer chiral centers, and a higher proportion of peripheral groups that contain oxygen.

Among the synthetic MC collections, A–F, some achieve quite good overlap with Zone 2, but none significantly samples either Zone 1 or Zone 3. This result may arise from the use, during the design of some of these compound libraries, of the Ro5 or other conventional metrics for druglikeness that are inapplicable to most known MC drug chemotypes.^2,4,40^ Another likely cause for their poor coverage of “MC druglike chemical space” is the previous lack of descriptors to capture many MC-specific structural features and properties, due to which libraries were necessarily designed without consideration of some properties that we have shown are characteristic of MC drugs. Other extant synthetic MC sets, not included in our current analysis, may show greater overlap with the oral MC drug zones. Developing synthetic MCs that more fully explore “MC druglike chemical space”, and especially Zones 1 and 3, will not necessarily involve chemotypes that closely resemble the natural product-derived drugs that currently define them. However, it will require chemistries that give access to MC chemotypes that, compared with the synthetic sets included here, have more complex and densely decorated rings, with a greater variety of substituents of different sizes and higher polar atom content, a greater density and variety of peripheral groups, more sp^3^ carbons, and more chiral centers. Our methodology provides an approach to determining the diversity of existing or new synthetic MC collections, and how well they sample the regions of property space where known MC drugs are found. Although the variety of synthetic MC chemotypes included in our study was not exhaustive, our analysis suggests that there may be substantial regions of property space left uncharted by previous synthetic efforts. For example, examination of Fig. 4B reveals the existence of a channel separating the oral MC drugs that define Zones 1 and 2 that is unoccupied by any chemotype we examined (seen best in the plot of PC1 *vs.* PC3), and the areas surrounding parts of Zone 1 and the region separating Zone 1 from Zone 3 are similarly unpopulated. Whether any of these unexplored regions will include compounds with interesting or useful properties is unclear.

We demonstrated two approaches to generating and evaluating new MC designs. One uses target ranges for the 13 properties that were identified by PCA as being the most important for distinguishing the MC sets from each other. We showed that applying specified value ranges for these 13 properties provided good discrimination between oral and non-oral MC drugs and clinical candidates, and that the number of property violations from among these 13 target ranges provides a surrogate for how far a structure lies from the center of oral druglike property space, in Zone 1. A chemist can easily assess any compound design with respect to these property ranges using only a pencil and paper. These guidelines would require modification to direct the design of MCs to other regions of MC chemical space, such as Zones 2 or 3, but such modified guidelines can easily be developed, if desired, from the results presented herein. The second design approach uses the themes we deduced for the dominant PCs 1 and 2 as broad guidance for how a starting chemotype might be modified to move it closer to any desired region of MC property space, for example for the purpose of refining library design. This approach is more flexible and intuitive than the first, but may require several rounds of design followed by mapping of the resulting structures onto chemical space.

The 13 most important properties from the PCA (Table 2 and Fig. S7†) include measures of molecular polarity, flexibility, and structural complexity. In terms of polarity, the well-known descriptors of cLogP and tPSA appear among this group. For the oral MC drugs, tPSA typically falls in the range 50–230 Å^2^, consistent with previous observations^1,2^ that oral MC drugs and other bRo5 compounds can have a tPSA that is substantially greater than the 140 Å^2^ considered the upper limit for conventional oral drugs.^40^ In contrast, clog *P* values for the MCs typically fall in the range ∼2–6, not dissimilar to the range for other oral drugs.^2^ Other important properties that pertain to polarity of the compound are MC-specific descriptors that address the polarity of the MC ring and its pendent peripheral groups. One such property, RingHet/*N* (MolD 22), is the fraction of MC ring atoms that are hereroatoms. For oral MC drugs, in the median case only one out of every 7–8 ring atoms (13%) is a heteroatom, and this proportion rarely exceeds 30%. In contrast, peripheral groups tend to be quite polar; the proportion that are oxygens (PeriphO/PeriphHA, 46) tends to lie between one-third and two-thirds, with the remaining peripheral groups mostly being methyls, since peripheral nitrogens (and halogens) are relatively rare ((PeriphN + 1)/(PeriphO + 1), 16). We speculate that this high preference for peripheral oxygens over nitrogens may be because carbonyls enable high peripheral polarity without introducing hydrogen bond donors. The implication that the MC ring tends to be relatively hydrophobic while the peripheral groups are more polar is consistent with trends we previously reported for natural product MCs that appear as ligands in the Protein Data Bank.^2,13^

Seven of the 13 most important properties address the structural complexity of the compounds. Three of these concern how substituents are distributed around the MC ring. These include Max Gap Size/*N* (76), Min Gap Size/*N* (77) and St. Dev. of Gap Size/*N* (79), the latter capturing the extent to which substituents tend to be clustered together *versus* evenly distributed around the MC ring. The oral MC drugs tend to have relatively densely decorated rings, corresponding to low values for both the largest and smallest gap sizes. In particular, it is uncommon for an oral MC drug to have a large portion of the MC ring that contains no substituents. A perfectly even distribution of substituents is generally not seen. As a separate metric of structural complexity, we note that there is no compound among the oral MC drugs and clinical candidates that has a substituent that connects to the main MC ring *via* a spiro fusion (spiro rings, 35). A third aspect of structural complexity is captured by chiral centers/HA (18). For the oral MC drugs, in the median case one out of every six heavy atoms (17%) is a chiral carbon, and proportions up to 35% are seen. Whereas the prior descriptor refers to the complexity of the molecule as a whole, a separate important descriptor concerns the complexity of the substituents in particular, in terms of the fraction of substituent carbons that are sp^3^ hybridized (Substituent Fsp3, 64). For many of the oral drugs, >90% of their substituent carbons are sp^3^ hybridized, and substituent Fsp^3^ values less than 50% are relatively uncommon. The compounds with the lowest proportion of chiral centers and the lowest values for substituent Fsp^3^, both indicative of lower structural complexity,^56^ are the synthetic kinase inhibitors that define Zone 2.

The remainder of the 13 most important properties concern molecular flexibility. Restricted fraction (82) describes the proportion of bonds in the MC ring that are further rigidified, beyond the constraints on motion imposed by involvement in a ring, because they comprise a π bond, an amide bond, or are involved in a ring fusion. For the oral MC drugs, the distribution of values for this descriptor is bi-modal; one-third of the compounds – mostly the erythromycin-like antibiotics – contain 0–15% of rigidified bonds in the MC ring, while two-thirds of the drugs contain 24–44% rigidified bonds in the ring.

The finding that assessing just 13 key properties provides a measure of proximity to oral MC druglike property space does not imply that only these properties are important in determining MC druglikeness. Rather, these properties approximate orthogonal variables that other relevant properties tend to covary with in predictable ways, at least for the kinds MC chemotypes included in this analysis. Development of new chemistries leading to very different chemotypes could necessitate a revision of these design guidelines if this partly incidental covariance between certain properties is lost. Moreover, the correlation between number of property range violations and the distance from MC druglike space is imperfect, and it is unclear to what extent poor choices with regard to other property values outside the 13 could result in a non-druglike location even for compounds with few violations. This possibility may be guarded against, if desired, by checking compound designs by mapping them onto PC space using the manual calculations described here.

Importantly, our analysis does not identify which properties confer any particular desired behavior, but only the extent to which a property distinguishes one MC compound or set of compounds from another. Any inferences about which structural features are more or less desirable are predicated on the assumed desirability of resembling approved oral MC drugs and disclosed clinical candidates. However, we envision that a major value of these new descriptors is to allow the assessment of how MC-specific properties influence specific pharmaceutically-relevant behaviors, such as passive membrane permeability, in empirical quantitative structure–property relationship (QSPR) studies and hypothesis driven investigations. We have included a detailed description of each MolD and how to calculate it to facilitate such work. We have also included the information needed for MC chemotypes not included in this study to be mapped onto the same property space defined here, for direct comparison, by applying the same molecular descriptors and model coefficients.

In addition to providing approaches to making synthetic MCs better resemble known MC drugs, our results have other utility. By attempting to broadly describe MC chemical space, we provide a means to design compounds that occupy currently unexplored or under-explored regions, thereby potentially facilitating discovery of new MC chemotypes. Our approach also establishes a more nuanced way to assess the structural diversity of MC libraries. Finally, as future oral MCs are discovered, this approach can reveal when newly discovered compounds with good druglike compounds represent a substantially new solution to the problem of MC druglikeness.

## Methods

### Molecular descriptors

Definitions and detailed descriptions of all 90 molecular descriptors (MolDs) used in this study are provided in Supplemental Document A.† MolDs were calculated using JChem Base (ChemAxon), employing a combination of standard chemical terms, calculator plugins, and custom molecular analysis algorithms scripted using ChemAxon's Java API.

Certain compound structures returned exceptions during the property calculations due to limitations of our current molecular analysis algorithms. Specifically, we found that the algorithm as written was unable to process polycyclic compounds containing *peri*-fusions to the MC; that is, two or more rings that are fused to the MC ring and fused to each other (a situation we termed “multi-fusion” exceptions). In these cases, the algorithm could not determine whether such substructures comprised one or multiple substituents or how to count the number of ring fusions as defined. Compounds with *peri*-fusions to the MC were identified by the algorithm as exceptions and routed to a second output file containing all MCs which returned exceptions at any point in the analysis. In the case of multi-fusion exceptions occurring in the class of oral drugs and clinical candidates, to avoid losing compounds from this key set we hand-calculated the property values for the subset of descriptors not processed by the algorithm, using the detailed descriptions of each MolD provided in Supplemental Document A.† Briefly, in the case of *peri*-fusions to the MC ring, the multi-fusion substituent was considered as one fused substituent and not divided arbitrarily. For the large compound sets it was not practical to hand-calculate properties, and so compounds returning this multi-fusion exception were excluded from analysis. Such compounds were found in MC Set C (<1% of compounds in that set) and Set E (<8%).

### Compound collections

The oral MC drugs and clinical candidates (Table S1†) and non-oral MC drugs and clinical candidates (Table S2†) were curated from the literature,^1,15^ the U.S. Food & Drug Administration,^26^ and from clinicaltrials.gov,^66^ and includes compounds reported prior to the end of 2018. Compounds were classed as “oral” only if there was published evidence of systemic distribution after oral administration in humans. Consequently, drugs such as plecanatide, nystatin, *etc.* that are dosed orally in some instances (*e.g.* for gastrointestinal diseases), but are documented to not achieve systemic distribution on oral dosing, were classed as “non-oral” for the purposes of this analysis. Conversely, a small number of compounds that are administered parenterally were excluded from the analysis altogether because we could not confirm that they have no systemic distribution if dosed orally. These compounds were lonodelestat, balixafortide, romidepsin, and zotarolimus. Compounds that had failed in the clinic or whose development has been discontinued were not included, to avoid contaminating the drug sets with compounds that might have unknown liabilities. Porphyrins such as cyanobobalamin were also excluded, on the grounds that their metal binding properties render them pharmaceutically and functionally distinct from typical MC drugs.

We additionally acquired structures for six synthetic MC sets from different sources. Compound Set A comprises 106 synthetic MCs from the collection of the Boston University Center for Molecular Discovery (BU-CMD). Compound Sets B–F are large (>1000 compounds), available from various commercial suppliers who market libraries of macrocyclic compounds. Compound structures from the above sources were filtered to exclude those MCs with a largest ring size of <11 heavy atoms, calculated using the LargestRingSize calculator in JChem (JChem Base API, Version 18.12.0, ChemAxon, Budapest, Hungary).

### Preparation of data

To eliminate bias due to the widely differing sizes of the compound sets, we selected representative compounds from each collection using *k*-medoids clustering^52,53^ with respect to all 90 MolDs. We set *k* = 42 to match the number of oral MC drugs and clinical candidates, which constituted the smallest set. The data were *Z*-scored prior to clustering.^67^ All 90 MolDs were calculated for the 42 representative compounds of each of the seven compound sets plus the oral MC drugs and clinical candidates, totaling 336 compounds. The values for each MolD were then rescaled to avoid biasing the subsequent analyses towards the descriptors with the largest numerical value ranges. Discrete properties, such as atom counts, were scaled from zero to the highest value observed for any compound in the analysis (eqn (1)):1
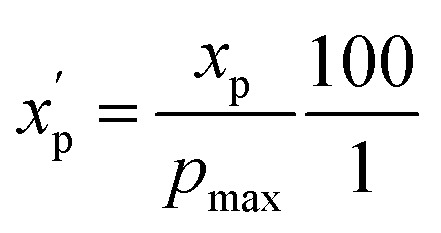
where *x*_p_ is the value of property p for a given compound, *p*_max_ is the maximum value of property *p* over all compounds, and 
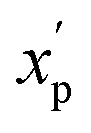
is the scaled value. The continuous properties molecular weight (MW), topological polar surface area (tPSA), and calculated log of the octanol–water partition coefficient (clog *P*), were scaled to the range of values spanning ±2 standard deviations (*σ*_p_) from the mean (*μ*_p_), calculated over all compounds (eqn (2)):2
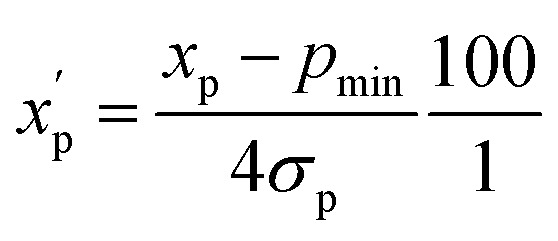
where *x*_p_ is the value of property p for a given compound, *p*_min_ = *μ*_p_ − 2*σ*_p_, and 
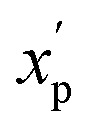
is the scaled value. Thus, values of these continuous properties that are outside ±2 standard deviations from the mean will have normalized values of >100 or <0. The *p*_max_ values for the discrete properties, and the *p*_min_ values and standard deviations for the continuous properties, are provided in Table S3.†

### Principal component analysis (PCA)

Principal Component Analysis (PCA) was performed in MATLAB (MATLAB v8.0 and Statistics Toolbox 8.1, The MathWorks, Inc., Natick, Massachusetts), using the singular value decomposition (SVD) algorithm with no *Z*-scoring of the data. No variable weighting was used as we had already rescaled the MolDs as described above. All other settings were at the default values.

### Hyperellipsoid volume calculations

The volume of property space encompassed by a given compound set, in 10-dimensional PC space, was approximated by calculating the volume of a 10-dimensional hyperellipsoid with a diameter along each PC axis corresponding to the range of the scores in that PC. We defined the range, *R*, for each PC as the range of compound scores in that PC that encompassed the middle 95% of the compounds. We chose this cut-off to avoid the possibility that the volume of chemical space sampled by a particular MC set might be unduly enlarged to encompass a single distant outlier compound. The volume, *V*, of the 10-dimensional hyperelliposid was calculated as:3
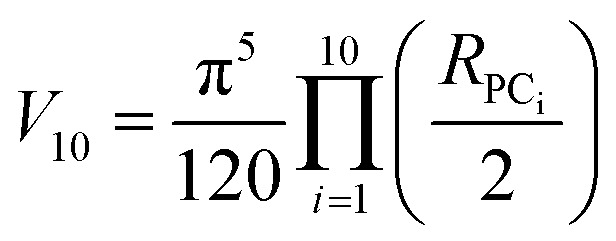
where *R*_PC_i__ is the range of values observed for PC_i_. This equation can be simplified by first calculating the volume of a hypercuboid, *B*, that would contain the hyperellipsoid in 10-dimensions, given simply by the product of the ranges, *R*, in each PC:
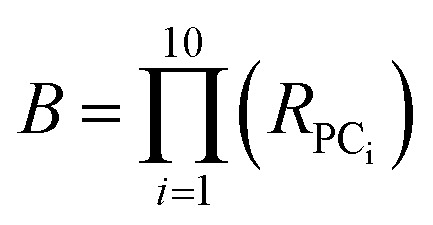


Substituting into eqn (3) gives:4
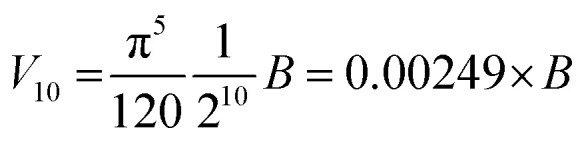


To plot the hyperellipsoids in PC space we defined the center of the ellipsoid as the center of mass of the compound set in question, which has coordinates corresponding to the mean score for the compounds in each PC dimension. The semi-axis of the ellipsoid in each PC axis was set equal to 0.5*R* for that PC, where *R* is the range of compound scores for that PC that encompasses 95% of the compounds.

### Distance of compounds and compound sets from the centroid of oral MC drug space

A variant of Hotelling's *t*^2^ statistic^68^ was devised to calculate the Euclidean distance, in 10-dimensional property space, between the center of mass of one compound set and that of another. To aid interpretation of the results, this distance was normalized by the semi-axis in PC1 of the hyperellipsoid that encompasses all compounds used in the current study.5
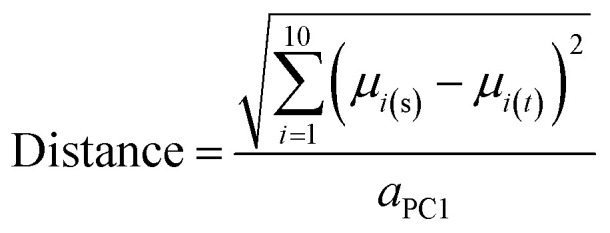
where *μ*_i(*s*)_ and *μ*_i(*t*)_ are the mean scores in PC_i_ for the compounds in the two sets, *s* and *t*, that are being compared, and *a*_PC1_ is the semi-axis of the “MC universe” with respect to PC1.

To express the distance of individual compound designs from the center of mass of the oral MC drug set, we modified eqn (5) as follows:6
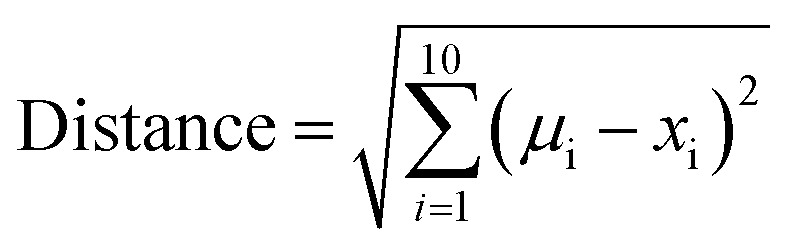
where *x*_i_ is the score for the particular compound in PC_i_, *μ* is the mean score for the oral MC drug set in PC_i_.

### Plotting the locations of compound designs in PC space

To plot new compound designs in the PC space defined by PCA of the original compound sets, values for the 90 MolDs were calculated for the new compound designs as described above. The MolD values were scaled in the same manner as the original MC set data, using eqn (1) and (2) and the values of *p*_min_, *p*_max_, *μ*_p_ and/or *σ*_p_ given in Table S3.† The results were projected onto the PCs using eqn (7).

7 Scores = data × coefficients^T^

The coefficients used for the transformation of compound MolD values to PC coordinates are provided in Table S3.† Using these, any compound design can be mapped onto the PC space described in Fig. 4, as illustrated for our own test designs in Fig. 6.

### Calculating importance of properties in PCA

The importance, *I*_p_, of each MolD property, *p*, for explaining the variance between the compounds was calculated by summing the squared coefficient of that property in each of the first 10 PCs, and multiplying by the total variance of that property, *V*_*p*_:8
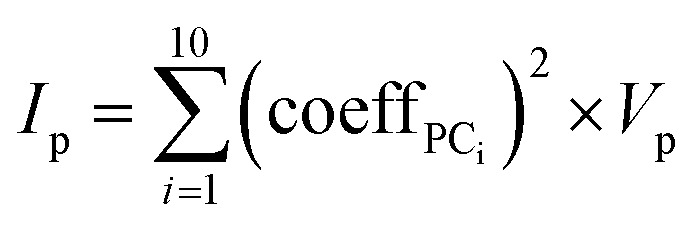
where the variance of each property, *V*_p_, is the sum over all compounds of the squared difference between the property value for compound i, *x*_i_, and the mean property value for all compounds, *μ*, all divided by the number of compounds, *n*.9
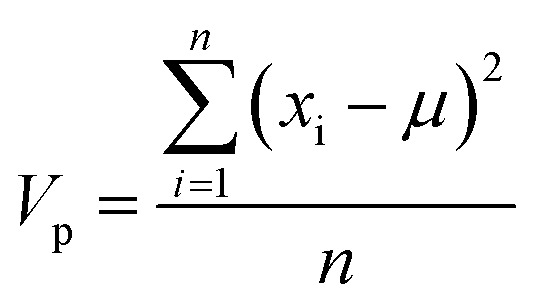


The property values used for this calculation are the MolD values scaled as described above. The results were ranked from highest to lowest, and the top 20 MolDs were identified.

To allow identification and elimination of properties that had a high correlation and therefore capture redundant information about the MC structures, we calculated the covariance between these top 20 properties. The properties were clustered with respect to the absolute values of the Pearson correlation coefficients of each property with respect to the others. When high covariance between properties was found, only the descriptor ranking highest in importance was retained. The 13 important and non-redundant MolDs that resulted are listed in Table 2.

Having identified these 13 key properties from PCA, we examined what range of values for each property is characteristic of the 42 oral MC drugs and clinical candidates (Fig. S7†). We found that the property distributions fall into one of three types: (A) normal distribution; (B) unimodal but asymmetric, further classified as either high-end or low-end biased; and (C) bimodal or multimodal. Property ranges were calculated for each property to encompass 80% of the compounds across all MC sets. For properties with distribution modalities A and C, the value range encompassing the middle 80% of the data was used; for properties with distribution modality B, the value range that included 80% of the data was defined starting from the upper or lower extreme of the distribution, depending on the direction of the bias.

## Author contributions

Conceptualization, A. W., L. V-B., and L. E. B.; methodology, L. V-B., A. W., and L. E. B.; software, L. E. B. and L. V-B.; formal analysis, L. V-B. and A. W.; validation, L. V-B, A. W., L. E. B. and A. A. R.; visualization, L. V-B. and A. W.; writing – original draft, L. V-B. and A. W.; writing – review & editing, L. V-B, A. W., L. E. B., and A. A. R.; funding acquisition, A. W. and L. E. B.; supervision, A. W.

## Conflicts of interest

AW and AR each serve as paid consultants to different companies on, among other subjects, the effective use of macrocycles in drug discovery. AW and LV-B have received funding and in-kind contributions of compounds from commercial sources to support academic research on this topic.

## Supplementary Material

SC-012-D0SC05788F-s001

SC-012-D0SC05788F-s002

SC-012-D0SC05788F-s003

SC-012-D0SC05788F-s004

SC-012-D0SC05788F-s005

SC-012-D0SC05788F-s006
