# Supplementary material for: Defining and navigating macrocycle chemical space
Source: Chem Sci. 2021 Mar 4;12(12):4309–28. doi: 10.1039/d0sc05788f (PMC8179434; doi:10.1039/d0sc05788f)
Supplement: SC-012-D0SC05788F-s001 [file SC-012-D0SC05788F-s001.pdf]

Electronic Supplementary Material (ESI) for Chemical Science.
This journal is © The Royal Society of Chemistry 2021

Defining and Navigating Macrocycle Chemical Space

Lauren A. Viarengo-Baker, Lauren E. Brown, Anna A. Rzepiela, and Adrian Whitty*

SUPPLEMENTAL DOCUMENT A: Detailed description of molecular descriptors used

General Notes and Definitions

a) Macrocycle analysis algorithms were developed using the JChem Base API (ChemAxon Version 18.12.0), with Java code written, compiled, and executed using the Eclipse IDE Version: 2019-09 R (4.13.0). Descriptors for each macrocycle were generated using a combination of (**1**) standard methods obtained from the JChem Base classes Molecule and MolAtom, and CalculatorPlugin subclasses such as ElementalAnalyserPlugin, TopologyAnalyserPlugin, logPPlugin, HBDAPlugin, and TPSAPlugin; and (**2**) custom-written Java algorithms for new macrocycle-specific descriptors, which rely on the atom type and connectivity information obtained from the methods of (**1**).

In cases where there was no published documentation to explicitly describe how ChemAxon makes a count or assigns a priority, where possible we inferred the rule by examining the results of the ChemAxon calculations for specific compounds. In case of any differences between how ChemAxon computes a given property for a particular molecular situation and the description provided here, the ChemAxon algorithm should be considered definitive.

The following notes are intended to provide a conceptual explanation of the descriptors, to aid the reader in understanding what feature of MC structure or properties each descriptor is intended to capture. These are also the definitions we used to hand-calculate the affected properties for the subset of structurally very complex MC drugs and clinical candidates for which ChemAxon returned a "multi-fusion exception" (see below).

b) Molecule Standardization: To ensure that all analyses and atom counts were limited to heavy atoms unless otherwise specified, all molecules were subjected to explicit hydrogen removal prior to analysis using the convertExplicitHtoImplicit() method from the class Hydrogenize. To ensure proper recognition of aromatic bonds, all molecules were aromatized prior to analysis using the aromatize() method from the class Molecule.

c) **Exception Handling:** During the course of the analysis, the following types of molecules, when identified, were diverted to a separate output without further analysis:

   (i) Multi-object molecules

   (ii) Molecules with largest ring size <10 atoms

   (iii) Molecules with *peri*-fusions to the macrocycle (see (k) "Multi-fusion exception", below)

d) The values ***HA* (heavy atom count) and *N* (macrocycle size)** used to normalize several molecular descriptors described below were obtained using the getAtomCount() method from class Molecule and getLargestRingSize() method from class TopologyAnalyserPlugin, respectively.

e) **Substituent:** A group containing ≥2 heavy atoms that is attached to the MC ring. A substituent can be attached to the MC ring at a single point, or at multiple points (e.g. fused or spiro substituents).

f) **Peripheral Group:** A group attached to the MC ring that contains a single heavy atom. A peripheral group can be attached to the MC ring at a single point, or at multiple points (e.g. epoxide, cyclopropane, or aziridine).

g) **Identifying the main MC ring.** Many of the calculations described below employ a main MC analysis algorithm which (1) identifies the atoms that comprise the largest (MC) ring in the molecule using the method getLargestRing() from class TopologyAnalyserPlugin; (2) bins all heavy atoms in the molecule as either MC ring or non-MC ring atoms; (3) analyzes the atom connectivity among non-MC ring atoms to (a) establish unique "branches" off of the MC ring, (b) identify every non-MC ring atom that makes up each branch, and (c) identify the MC ring atom(s) to which these branches are connected; (4) identifies and merges any overlapping branches caused by fusions or spiro rings; and (5) determines the size of each branch to further bin its atoms as either substituent (branch size >1) or peripheral (branch size = 1) atoms.

h) **Definition of Ring Size (*N*):** The size of the MC ring was determined by ChemAxon using a Smallest Set of Smallest Rings (SSSR) algorithm[1]. There are various approaches to enacting SSSR, but examination of values calculated by ChemAxon for specific structures indicates that *N* corresponds to the number of atoms that provide the <u>shortest</u> path around the ring that

returns to the atom at which the count was started. In compounds containing multiple rings, the main MC ring was defined as the largest ring, as determined using this shortest-path method. Thus, for example, the non-oral MC drug **oritavancin**, shown below, was defined as a system of three fused macrocycle rings, of sizes 16, 16 and 12, rather than as a single 34-membered macrocyclic ring with internal bridging atoms. Consequently, for the purposes of this study, oritavancin is considered to be a 16-membered macrocycle.

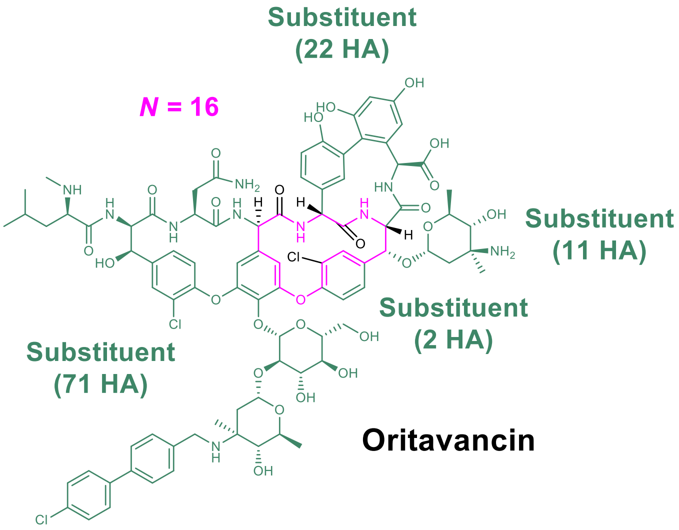

In cases where a compound contained two or more "largest rings" of equal size, examination of the assignment made by ChemAxon to specific structures suggests that in such cases the more central – i.e. the one with the greatest number of other large rings attached – was considered the main MC ring (see **oritavancin**, above). The other equally sized or smaller rings were considered to be substituents attached to the main MC ring. While this distinction doesn't affect the assigned value of the MC ring size, *N*, it is important because it potentially affects the values of other descriptors that capture the structure and composition of the ring and its substituents and peripheral groups.

A small number of compounds in the study contained four large rings that included two equally central rings of the same size (e.g. **dalbavancin**, shown below left, which by our definition comprises a 14-16-16-12 system). In these cases, examination of the assignment made by ChemAxon to specific multi-ringed structures suggests that in such cases the ring with the largest rings attached was considered to be the main MC ring. Thus, for dalbavancin, the 16-membered ring indicated with an asterisk, which is directly fused to a 16- and a 14-membered ring, is considered paramount over the other 16-membered ring which is fused to

a 16- and a 12-membered ring. In cases of this kind, the entire fused ring system containing the 16- and 12-membered rings is considered as a single large substituent off the main (asterisked) 16-membered MC ring.

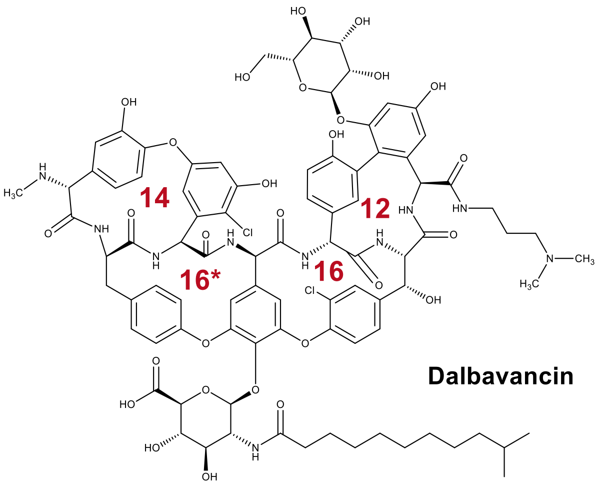

Dalbavancin

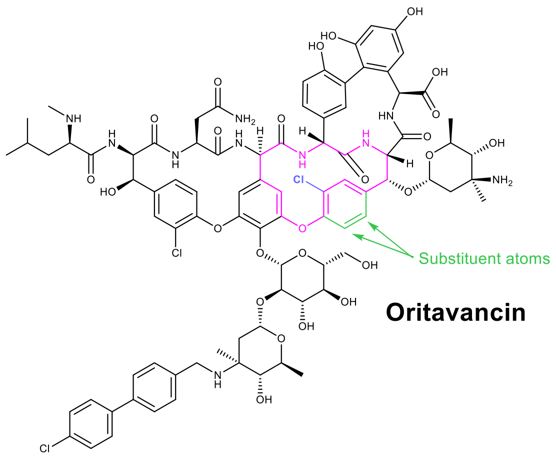

Oritavancin

i) **Treatment of MC rings containing in-line aromatic groups:** There are several examples among the non-oral and oral MC drugs where tracing the MC ring is complicated by "in-line" aromatics – that is, by phenyl or other 6-membered aromatic ring connected to the rest of the MC ring at its 1- and 4-positions. When such an in-line aromatic ring is unsubstituted, considering the MC ring to trace either of the two possible paths around the aromatic group results in the same values for all descriptors. However, if the aromatic group is substituted asymmetrically, so as to make the two paths around the group nonequivalent, then it becomes necessary to define which route defines the main MC ring. This point is illustrated by **oritavancin** (above right), which contains a chlorophenyl group in-line with the main MC ring. Depending on which route around the aromatic is considered to define the MC ring, the chlorophenyl structure can be seen either as a substituent with formula CH.CCl, or alternatively as a substituent with formula CH.CH with the chlorine being a peripheral group on the MC ring. In these instances, examination of the assignment made by ChemAxon to specific multi-ringed structures suggests that in such cases the MC ring is traced to include the greatest number of elements of decoration. Thus, in the case of oritavancin the MC ring follows the path around the edge of the in-line aromatic contained the chlorine substituent

(colored magenta in the figure shown above right), so as to define the MC ring as having a peripheral chlorine (blue) plus a two-carbon substituent (green).

j) **Multi-fusion exception**: The algorithm used to calculate certain descriptors gave a "multi-fusion exception" for polycyclic compounds containing *peri*-fusions to the MC; that is, compounds containing two or more smaller rings fused both to the main MC ring and to each other. In the case of such *peri*-fusions, the multi-fusion substituent was considered to be a single substituent rather than being divided arbitrarily. The oral clinical candidate **AMG-176** (shown below) exemplifies how substituents were defined when hand-calculating such cases, with the 7-6 ring system fused to the main MC ring, plus its attached atoms, being considered as a single substituent of composition $C_{15}H_{15}ClO$ (17 heavy atoms). Another example is provided by **Oritavancin** (below right), in which the 16-6 system fused to the left-hand side of the MC ring, plus its attached atoms, are considered to be a single large substituent of 71 heavy atoms.

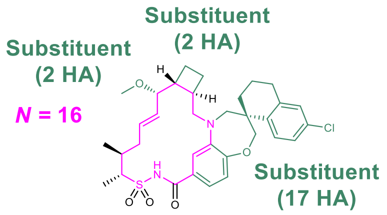

AMG-176

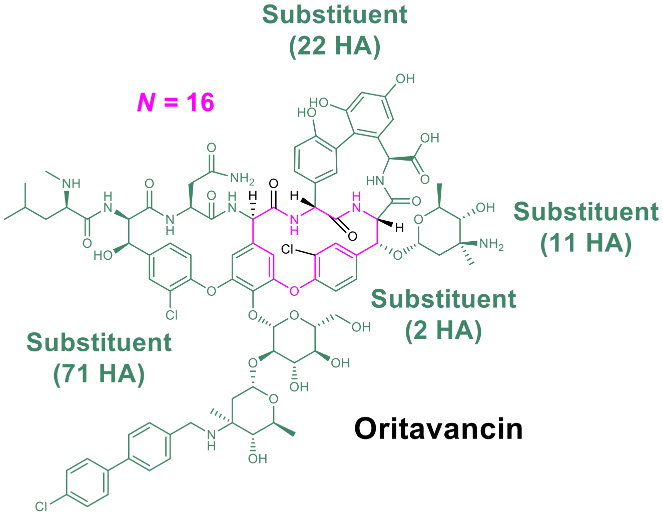

Oritavancin

Molecular Descriptors

Descriptor numbers cited in the main text refer to the numbers used in the following list.

Standard Deviations are population standard deviations calculated using an equation equivalent to that used to calculate StDev.P in Microsoft Excel.

Format of the MolD descriptions that follow:
Descriptor No. **Descriptor Name (abbreviated name):** Brief description, with origin where applicable.

Script or pseudocode used to calculate descriptor, with classes and methods from the JChem Base API specified where applicable.

***HA*:** Number of heavy atoms in the molecule. Used to normalize many of the descriptors employed below.

Directly obtained from method getAtomCount() from class Molecule

1. **Molecular Weight (MW)**: A classical druglikeness metric included in Lipinski's Rule of 5 (Ro5)[2]. Lipinski compliant compounds have MW ≤ 500 Da. MW is considered a rough surrogate for molecular volume.
   Directly obtained from method getExactMass() from class ElementalAnalyserPlugin

2. **Hydrogen Bond Donors (HBD):** Total count of NH plus OH groups. A classic druglikeness metric included in Lipinski's Rule of 5[2]. Ro5 compliant compounds have HBD ≤ 5.
   Directly obtained from method getDonorAtomCount() from class HBDAPlugin

3. **Hydrogen Bond Donors, Normalized (HBD/HA)**: Number of HBD, from MolD_2, normalized to the overall size of the molecule expressed in terms of the number of heavy (i.e. non-hydrogen) atoms.
   Calculated from values obtained above using the equation:
   [Hydrogen Bond Donors]/[HA]

4. **Hydrogen Bond Acceptors (HBA):** Total count of O, N and F atoms with an available lone pair. A classic druglikeness metric included in Lipinski's Rule of 5[2]. Ro5 compliant compounds have HBA ≤ 10.
   Directly obtained from method getAcceptorAtomCount() from class HBDAPlugin

5. **Hydrogen Bond Acceptors, Normalized (HBA/HA)**: Number of HBA, from MolD_4, normalized to the overall size of the molecule expressed in terms of the number of heavy (i.e. non-hydrogen) atoms.
   Calculated from values obtained above using the equation:
   [Hydrogen Bond Acceptors]/[HA]

6. **Calculated Logarithm of the Octanol-Water Partition Constant (cLogP):** A classic druglikeness metric included in Lipinski's Rule of 5[2]. Ro5 compliant compounds have cLogP ≤ 5.
   Directly obtained from method getLogPTrue() from class LogPPlugin

7. **Number of Rotatable Bonds (NRB):** A classic druglikeness metric included in Veber's Rules[3]. Veber compliant compounds have NRB ≤ 10, with NRB ≤ 7 being optimal. Considered a surrogate for molecular flexibility.
   Directly obtained from method getRotatableBondCount() from class TopologyAnalyserPlugin

8. **Topological Polar Surface Area (tPSA):** A classic druglikeness metric included in Veber's Rules[3]. Calculated by summing the surface area of all polar atoms and any attached hydrogens. This metric does not take into account the three-dimensional conformation of the molecule, and thus does not account for the fact that certain polar atoms may be shielded from solvent in particular conformations. Veber compliant compounds have tPSA ≤ 140 $Å^2$.
   Directly obtained from method getSurfaceArea() from class TPSAPlugin

9. **Largest Ring Size (*N*):** The number of atoms in the main MC ring. The size of the MC ring was defined as the largest ring in the molecule according to SSSR notation. See notes on pages 1-5 of this document for a description of how the main MC ring was defined in special situations such as compounds containing multiple large rings, or compounds for which the algorithm returns a "multi-fusion exception" error.
   Directly obtained from method getLargestRingSize() from class TopologyAnalyserPlugin

10. **Number of Large Substituents:** A count of substituents attached to the main MC ring that contain ≥ 5 heavy atoms, following the definition of Villar *et al.*[4]
    numLargeSubs is calculated with an algorithm that cycles through substituents and counts if size is greater than or equal to 5

11. **Number of Small Substituents:** A count of substituents attached to the main MC ring that contain 2-4 heavy atoms, following the definition of Villar *et al.* [4].
    numSmallSubs is calculated with algorithm that cycles through substituents and counts if size is less than 5

12. **Substituent Heavy Atoms, Normalized (subHA/HA):** The number of heavy atoms that are in substituents expressed as a fraction of total heavy atoms in the molecule.
    The number of substituent atoms (numSubstituentAtoms) is calculated using the general MC analysis algorithm as described above. This value is then normalized with the equation:
    subHeavyAtomsOverHeavyAtoms = (numSubstituentAtoms)/HA

13. **Number of Substituents, Normalized (SubCount/N):** Total number of substituents (large + small) normalized by MC ring size, *N*, to give substituents per MC ring atom.
    Calculated from values obtained above:
        subCountOverN = ([numSmallSubs]+[numLargeSubs])/N

14. **Number of Peripheral Groups, Normalized (PeriphCount/N):** Total number of peripheral groups normalized by MC ring size, *N*, to give peripheral groups per MC ring atom.
    The number of peripheral groups (numPeripheralGroups, which is by extension equivalent to the number of peripheral atoms, numPeripheralAtoms) is calculated using the general MC analysis algorithm as described above. This value is then normalized with the equation:
    periphCountOverN = (numPeripheralGroups)/N

15. **Ratio of Substituent Nitrogens to Substituent Oxygens (SubN+1)/(SubO+1):** Ratio of nitrogen to oxygen atoms summed over all substituents. Note: 1 is added to both numerator and denominator to avoid division by zero errors.
    The number of substituent nitrogens and oxygens are counted using an algorithm that cycles through binned substituent atoms and checks atom type using the method getAtomicNumber() from the class Molecule. These values are plugged into the equation:
    subNtoO_Mod = ((numSubstituentNitrogens+1))/(numSubstituentOxygens+1)

16. **Ratio of Peripheral Nitrogens to Peripheral Oxygens (PeriphN+1)/(PeriphO+1):** Ratio of nitrogen to oxygen atoms, considering only peripheral groups. Note: 1 is added to both numerator and denominator to avoid division by zero errors.
    The number of peripheral nitrogens (numPeripheralNitrogens) and oxygens (numPeripheralOxygens) are counted using an algorithm that cycles through binned peripheral atoms and checks atom type using the method getAtomicNumber() from the class Molecule. These values are plugged into the equation:
    periphNtoO_Mod = ((numPeripheralNitrogens+1))/(numPeripheralOxygens+1)

17. **Ratio of Nitrogens to Oxygens (TotalN+1)/(TotalO+1):** The ratio of nitrogen to oxygen atoms in the molecule. Villar *et al.*[4] suggested, based on analysis of a limited number of compounds, that an average ratio of 0.25:1 with an observed range of 0–0.4:1 was typical of large macrocycles. Note: 1 is added to both numerator and denominator to avoid division by zero errors.
    The total number of nitrogens and oxygens in the molecule are counted using the method getAtomCount() from class ElementalAnalyserPlugin, and plugged into the equation:
        totalNtoO_Mod = (numNitrogens+1)/(numOxygens+1)

18. **Chiral Centers, Normalized (ChiralCenters/HA):** The number of chiral centers in the molecule normalized to the size of the molecule as measured in heavy atoms. Villar *et al.*[4]

suggested, based on analysis of a limited number of compounds, that an average of 15 chiral centers with an observed range of 9-18 was typical of large macrocycles.

The number of chiral centers (numChiralCenters) in the molecule are directly obtained from the method getChiralCenterCount() from class TopologyAnalyserPlugin. The normalized value is calculated from the equation:

chiralCentersOverHeavyAtoms = (numChiralCenters)/HA

19. **Chiral Centers in the Macrocycle Ring, Normalized (RingChiralCenters/N):** Count of carbon atoms in the main MC ring that are chiral centers, normalized for overall ring size, *N*, to give chiral centers per MC ring atom.

    The number of chiral centers in the MC ring (numRingChiralCenters) are counted using an algorithm which checks each MC atom using the method isChiralCenter() from class TopologyAnalyserPlugin. The normalized value is calculated from the equation:

    ringChiralCentersOverN = (numRingChiralCenters)/N

20. **Ring Size, Normalized (N/HA):** The macrocycle ring size normalized for the size of the overall molecule expressed in terms of heavy atoms. Represents the fraction of overall heavy atoms that are MC ring atoms.

    Calculated from values obtained above:

    NoverHA = N/HA

21. **Fraction of Atoms in Substituents and Peripheral Groups ((SubHA+PeriphHA)/HA):** Fraction of all heavy atoms in the compound that are in the substituents and peripheral groups (i.e. that are <u>not</u> MC ring atoms). Equals 1 – MolD_20.

    Calculated from values obtained above:

    subAndPeriphOverHA = (numSubstituentAtoms+numPeripheralGroups)/HA

22. **Macrocycle Ring Heteroatoms, Normalized (RingHet/N):** The fraction of atoms in the main MC ring that are heteroatoms (defined here as not carbons).

    The number of MC carbons (numMCcarbons) are calculated using an algorithm which cycles through binned MC atoms and checks atom type using the method getAtomicNumber() from the class Molecule. The number of MC heteroatoms (numMCheteroatoms) are calculated from the equation:

    numMCheteroatoms = N – numMCcarbons

    This value is further normalized using the equation:

    ringHetOverN = numMCheteroatoms/N

23. **Total Heteroatoms, Normalized (TotalHet/HA):** Count of heteroatoms in the compound, normalized to the size of the compound in terms of heavy atom count.

    The total number of carbons (numCarbons) in the molecule are counted using the method getAtomCount() from class ElementalAnalyserPlugin. The total number of heteroatoms in the molecule (numHeteroatoms) are calculated from the equation:

    numHeteroatoms = HA – numCarbons

This value is further normalized using the equation:

totalHetOverHA = numHeteroatoms/HA

24. **Macrocycle Ring Oxygens, Normalized (RingOxygen/N):** The fraction of atoms in the main MC ring that are oxygen (i.e number of –O– linkages in the ring/*N*).

    The number of MC oxygens (numMCoxygens) are calculated using an algorithm which cycles through binned MC atoms and checks atom type using the method getAtomicNumber() from the class Molecule. The value is further normalized using the equation:

    ringOxygenOverN = numMCoxygens/N

25. **Total Oxygens, Normalized (TotalOxygen/HA):** Count of oxygen atoms in the compound, normalized to the size of the compound in terms of heavy atom count.

    The total number of oxygens (numOxygens) in the molecule are counted using the method getAtomCount() from class ElementalAnalyserPlugin. This value is further normalized using the equation:

    totalOxygenOverHA = (numOxygens)/HA

26. **Macrocycle Ring Nitrogens, Normalized (RingNitrogens/N):** The fraction of atoms in the main MC ring that are nitrogen (i.e number of –NH– or –NR– linkages in the ring/*N*).

    The number of MC nitrogens (numMCnitrogens) are calculated using an algorithm which cycles through binned MC atoms and checks atom type using the method getAtomicNumber() from the class Molecule. The value is further normalized using the equation:

    ringNitrogenOverN = (numMCnitrogens)/N

27. **Total Nitrogens, Normalized (TotalNitrogen/HA):** Count of nitrogen atoms in the compound, normalized to the size of the compound in terms of heavy atom count.

    The total number of nitrogens (numNitrogens) in the molecule are counted using the method getAtomCount() from class ElementalAnalyserPlugin. This value is further normalized using the equation:

    totalNitrogenOverHA = (numNitrogens)/HA

28. **Peripheral Fluorines, Normalized (PeriphFluorine/N):** Count of fluorines in peripheral groups, normalized for macrocycle ring size.

    The number of peripheral fluorines (numPeripheralFluorines) are calculated using an algorithm which cycles through binned peripheral atoms and checks atom type using the method getAtomicNumber() from the class Molecule. The value is further normalized using the equation:

    peripheralFluorinesOverN = (numPeripheralFluorines)/N

29. **Total Fluorines, Normalized (TotalFluorine/HA):** Count of fluorine atoms in the compound, normalized to the size of the compound in terms of heavy atom count.

    The total number of fluorines (numFluorines) in the molecule are counted using the method getAtomCount() from class ElementalAnalyserPlugin. This value is further normalized using the equation:

    totalFluorinesOverHA = (numFluorines)/HA

30. **Peripheral Chlorines, Normalized (PeriphChlorines/N):** Count of chlorine atoms in peripheral groups, normalized for macrocycle ring size.

    The number of peripheral chlorines (numPeripheralChlorines) are calculated using an algorithm which cycles through binned peripheral atoms and checks atom type using the method getAtomicNumber() from the class Molecule. The value is further normalized using the equation:

    peripheralChlorinesOverN = (numPeripheralChlorines)/N

31. **Total Chlorines, Normalized (TotalChlorine/HA):** Count of chlorine atoms in the compound, normalized to the size of the compound in terms of heavy atom count.

    The total number of chlorines (numChlorines) in the molecule are counted using the method getAtomCount() from class ElementalAnalyserPlugin. This value is further normalized using the equation:

    totalChlorinesOverHA = (numChlorines)/HA

32. **Macrocycle Ring Sulfurs, Normalized (RingSulfur/N):** Count of sulfur atoms in the macrocycle ring, normalized for macrocycle ring size.

    The number of MC sulfurs (numMCsulfurs) are calculated using an algorithm which cycles through binned MC atoms and checks atom type using the method getAtomicNumber() from the class Molecule. The value is further normalized using the equation:

    ringSulfursOverN = (numMCsulfurs)/N

33. **Total Sulfurs, Normalized (TotalSulfur/HA):** Count of sulfur atoms in the compound, normalized to the size of the compound in terms of heavy atom count.

    The total number of sulfurs (numSulfurs) in the molecule are counted using the method getAtomCount() from class ElementalAnalyserPlugin. This value is further normalized using the equation:

    totalSulfursOverHA = (numSulfurs)/HA

34. **Total Aromatics, Normalized (TotalAromatics/HA):** Count of aromatic group atoms in the compound, normalized to the size of the compound in terms of heavy atom count.

    The total number of aromatic atoms in the molecule (numAromaticAtoms) are counted using the method getAromaticAtomCount() from the class TopologyAnalyserPlugin. The value is further normalized using the equation:

    totalAromaticsOverHA = (numAromaticAtoms)/HA

35. **Spiro Rings:** Count of substituents that are connected to the main MC ring *via* a spiro fusion. This descriptor concerns only attachment points to the main MC ring; Spiro rings in other parts of the molecule are ignored (see Figure below MolD_37).

The number of spiro ring substituents (numSpiroRings) is counted using the MC analysis algorithm described above. In brief, unique substituents with two different connection points to the MC ring which both occur at the same MC atom are flagged and counted as spiro rings.

36. **Fused Rings, Normalized (FusedRings/N):** Count of rings that are fused to the main MC ring, normalized by ring size. For this purpose, a "fused ring" is defined as a ring that is equal in size or smaller than the main MC ring, and that is attached to the MC ring at two different MC atoms (see Figure below MolD_37). This descriptor concerns only attachment points to the main MC ring; fused ring systems in other parts of the molecule are ignored. Where two or more fused rings connect to each other at one or more additional points distal to the MC ring (*peri*-fusions), these structures cannot be handled by the current algorithm and return exceptions. In cases where molecules containing *peri*-fusions were processed by hand (oral and non-oral drug MCs), a *peri*-fusions is considered to be a single substituent (see comment (j) in General Definitions and Notes, on pages 1-3 of this document), but each ring within it that is directly fused to the main ring is counted separately for the purpose of this descriptor.

The number of fused rings (numNonSpiroFusedRings) is counted using the MC analysis algorithm described above. In brief, unique substituents with two different connection points to the MC ring occurring at different MC atoms are flagged and counted as fused rings. The involved atoms and bonds are also flagged as being part of fusions. The number of fused rings is further normalized with the equation:

fusedRingsOverN = (numNonSpiroFusedRings)/N

37. **Bridges:** Count of bridges in the compound. For the purpose of this descriptor, a "bridge" is defined as a feature of fused rings, wherein the two connection points to the MC are separated by one or more atoms (see figure). Atoms <u>in the main MC ring</u> that lie between the connection points of the bridge are designated "bridging atoms". For clarity, the example of a bridged structure shown below right has a single bridging atom.

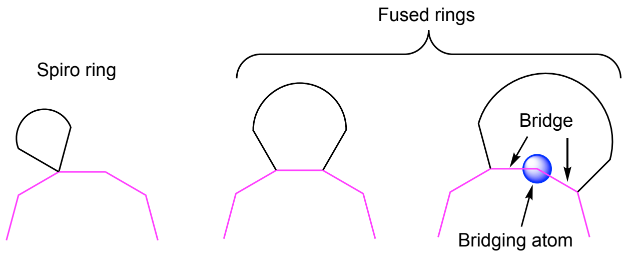

(Megenta line indicates the path of the "main" MC ring)

Pseudocode for identifying bridges:

- *Cycle through unique branches*

- *If the branch is involved in a non-spiro fusion, identify the two macrocycle atoms that it is connected to*
- *Check if these two MC atom connection points are directly bonded to one another.*
    - *If so, there is no bridge*
    - *If no, there must be bridging atom(s) between them.* ***numBridges*** *is the count of how many times this happens in the molecule.*
        - *For these two atoms, measure the two "distances apart" (the long way and the short way) – and choose the shorter of the two*
        - *Walk along the "shortest" route from connection point #1 to connection point #2. Any MC atoms lying between these two atoms are classified bridging atoms. <u>The connection points themselves are not bridging atoms.</u>*

38. **Substituent Nitrogens, Normalized (SubN/SubHA):** Count of nitrogen atoms in substituents, normalized to how many heavy atoms are in substituents.
    The number of substituent nitrogens (numSubstituentNitrogens) are calculated using an algorithm which cycles through binned substituent atoms and checks atom type using the method getAtomicNumber() from the class Molecule. The value is further normalized using the equation:
        subNoverSubHA = (numSubstituentNitrogens)/numSubstituentAtoms

39. **Substituent Oxygens, Normalized (SubO/SubHA):** Count of oxygen atoms in substituents, normalized to how many heavy atoms are in substituents.
    The number of substituent oxygens (numSubstituentOxygens) are calculated using an algorithm which cycles through binned substituent atoms and checks atom type using the method getAtomicNumber() from the class Molecule. The value is further normalized using the equation:
        subOoverSubHA = (numSubstituentOxygens)/numSubstituentAtoms

40. **Substituent Fluorines, Normalized (SubF/SubHA):** Count of fluorine atoms in substituents, normalized to how many heavy atoms are in substituents.
    The number of substituent fluorines (numSubstituentFluorines) are calculated using an algorithm which cycles through binned substituent atoms and checks atom type using the method getAtomicNumber() from the class Molecule. The value is further normalized using the equation:
        subFoverSubHA= (numSubstituentFluorines)/numSubstituentAtoms

41. **Substituent Chlorines, Normalized (SubCl/SubHA):** Count of chlorine atoms in substituents, normalized to how many heavy atoms are in substituents.
    The number of substituent chlorines (numSubstituentChlorines) are calculated using an algorithm which cycles through binned substituent atoms and checks atom type using the method getAtomicNumber() from the class Molecule. The value is further normalized using the equation:
        subClOverSubHA = (numSubstituentChlorines)/numSubstituentAtoms

42. **Substituent Sulfurs, Normalized (SubS/SubHA):** Count of sulfur atoms in substituents, normalized to how many heavy atoms are in substituents.

The number of substituent sulfurs (numSubstituentSulfurs) are calculated using an algorithm which cycles through binned substituent atoms and checks atom type using the method getAtomicNumber() from the class Molecule. The value is further normalized using the equation:

subSoverSubHA = (numSubstituentSulfurs)/numSubstituentAtoms

43. **Substituent Heteroatoms, Normalized (SubHeteroatoms/SubHA):** Count of heteroatoms (atoms that are not C or H) in substituents, normalized to how many heavy atoms are in substituents.

First, the number of substituent carbons (numSubstituentCarbons) are calculated using an algorithm which cycles through binned substituent heavy atoms and checks atom type using the method getAtomicNumber() from the class Molecule. The number of substituent heteroatoms (numSubstituentHeteroatoms) are calculated using the equation:

numSubstituentHeteroatoms = numSubstituentAtoms-numSubstituentCarbons

The value is further normalized using the equation:

subHetOverSubHA = (numSubstituentHeteroatoms)/numSubstituentAtoms

44. **Ratio of MC Ring Nitrogens to MC Ring Oxygens ((RingN+1)/(RingO+1)):** Ratio of nitrogen to oxygen atoms in the main macrocycle ring. Note: 1 is added to both numerator and denominator to avoid division by zero errors.

Calculated from values derived above using the equation:

ringNtoO_Mod = ((numMCnitrogens+1))/(numMCoxygens+1)

45. **Peripheral Nitrogens, Normalized (PeriphN/PeriphHA):** Count of nitrogen atoms in peripheral positions, normalized to the total number of peripheral heavy atoms.

Calculated from values derived above using the equation:

periphNoverPeriphHA = (numPeripheralNitrogens)/numPeripheralAtoms

46. **Peripheral Oxygens, Normalized (PeriphO/PeriphHA):** Count of oxygen atoms in peripheral positions, normalized to the total number of peripheral heavy atoms.

Calculated from values derived above using the equation:

periphOoverPeriphHA = (numPeripheralOxygens)/numPeripheralAtoms

47. **Peripheral Fluorines Normalized (PeriphF/PeriphHA):** Count of fluorine atoms in peripheral positions, normalized to the total number of peripheral heavy atoms.

Calculated from values derived above using the equation:

periphFoverPeriphHA = (numPeripheralFluorines)/numPeripheralAtoms

48. **Peripheral Chlorines Normalized (PeriphCl/PeriphHA):** Count of chlorine atoms in peripheral positions, normalized to the total number of peripheral heavy atoms.

Calculated from values derived above using the equation:

periphClOverPeriphHA = (numPeripheralChlorines)/numPeripheralAtoms

49. **Peripheral Sulfurs Normalized (PeriphS/PeriphHA):** Count of sulfure atoms in peripheral positions, normalized to the total number of peripheral heavy atoms.

    Calculated from values derived above using the equation:

    periphSoverPeriphHA = (numPeripheralSulfurs)/numPeripheralAtoms

50. **Peripheral Heteroatoms Normalized (PeriphHeteroatoms/PeriphHA):** Count of heteroatoms (atoms that are not C or H) in peripheral positions, normalized to the total number of peripheral heavy atoms.

    Calculated from values derived above using the equation:

    periphHetOverPeriphHA = (numPeripheralHeteroatoms)/numPeripheralAtoms

51. **Polar Surface Area, Normalized (Polar Surface Area/HA):** The topological polar surface area (tPSA) of the compound, from MolD_8) normalized to the size of the molecule expressed as total heavy atoms.

    Calculated from values derived above using the equation:

    psaOverHA = (polarSurfaceArea)/HA

52. **Carbonyls:** Count of carbonyls in the molecule.

    Uses an algorithm written to identify and count C=O double bonds in the molecule based on examining atom types and connectivity with standard Molecule, MolAtom, ElementalAnalyserPlugin and TopologyAnalyserPlugin class methods

53. **Carboxylates:** A count of carboxylates in the molecule.

    Uses an algorithm written to identify and count C=O(OH) functional groups in the molecule based on examining atom types and connectivity with standard Molecule, MolAtom, ElementalAnalyserPlugin and TopologyAnalyserPlugin class methods

54. **Alcohols:** A count of alcohols in the molecule.

    Uses an algorithm written to identify and count (tetrahedral)C-O-H groups in the molecule based on examining atom types and connectivity with standard Molecule, MolAtom, ElementalAnalyserPlugin and TopologyAnalyserPlugin class methods

55. **Phenols:** A count of phenols in the molecule.

    Uses an algorithm written to identify and count (aromatic)C-O-H groups in the molecule based on examining atom types and connectivity with standard Molecule, MolAtom, ElementalAnalyserPlugin and TopologyAnalyserPlugin class methods

56. **Primary Amines:** A count of primary amines in the molecule.

    Uses an algorithm written to identify and count nitrogens single bonded to one tetrahedral carbon and two hydrogens in the molecule based on examining atom types

and connectivity with standard Molecule, MolAtom, ElementalAnalyserPlugin and TopologyAnalyserPlugin class methods

57. **Secondary Amines:** A count of secondary amines in the molecule.
    Uses an algorithm written to identify and count nitrogens single bonded to two tetrahedral carbons and one hydrogen in the molecule based on examining atom types and connectivity with standard Molecule, MolAtom, ElementalAnalyserPlugin and TopologyAnalyserPlugin class methods

58. **Tertiary Amines:** A count of tertiary amines in the molecule.
    Uses an algorithm written to identify and count nitrogens single bonded to one tetrahedral carbons and two hydrogens in the molecule based on examining atom types and connectivity with standard Molecule, MolAtom, ElementalAnalyserPlugin and TopologyAnalyserPlugin class methods

59. **Primary Amides:** A count of primary amides in the molecule.
    Uses an algorithm written to identify and count nitrogens single bonded to one carbonyl carbon and two hydrogens in the molecule based on examining atom types and connectivity with standard Molecule, MolAtom, ElementalAnalyserPlugin and TopologyAnalyserPlugin class methods

60. **Secondary Amides:** A count of secondary amides in the molecule.
    Uses an algorithm written to identify and count nitrogens single bonded to one carbonyl carbon, one tetrahedral carbon, and one hydrogen in the molecule based on examining atom types and connectivity with standard Molecule, MolAtom, ElementalAnalyserPlugin and TopologyAnalyserPlugin class methods

61. **Tertiary Amides:** A count of tertiary amides in the molecule.
    Uses an algorithm written to identify and count nitrogens single bonded to one carbonyl carbon and two tetrahedral carbons in the molecule based on examining atom types and connectivity with standard Molecule, MolAtom, ElementalAnalyserPlugin and TopologyAnalyserPlugin class methods

62. **Molecule Total Degree of Unsaturation (DOU):** Count of the number of atomic valences on the molecule's heavy-atom framework that are not occupied by hydrogen or halogen. Primary, secondary, tertiary and quaternary carbon atoms are considered to have, respectively, 3, 2, 1 or 0 available positions for attachment to hydrogen/halogen; primary, secondary and tertiary nitrogen atoms are respectively considered to have 2, 1, and 0 positions; and primary and secondary oxygen atoms are considered to have 1 or zero positions. Sulfur atoms, which are rare among the chemotypes included in the analysis, are ignored. DOU is thus given by the following formula:

$$DOU = C - \frac{H}{2} - \frac{Hal}{2} + \frac{N}{2} + 1$$

Where C = carbons; H = hydrogens; Hal = halogens; N = Nitrogens.

For example, using this formula, an alkane will have DOU = 0, while a cycloalkane will have DOU = 1 because the ring closure represents a degree of unsaturation. A value of DOU = 1 is therefore the lowest value possible for a macrocycle, and would indicate that the compound has an exclusively sigma-bonded framework on which all remaining valences are saturated through boding to H or a halogen. The maximum possible value is DOU = C + N/2 + 1, corresponding to a situation in which all valences are engaged in sigma or pi bonds connecting the heavy atom framework of the compound, with no hydrogen of halogen atoms present.

> *Total halogens (numHalogens) in the molecule are calculated as the sum of total fluorines, chlorines, bromines and iodines as counted using methods described above. Molecule Total DOU is further calculated using the equation:*
>
> > *moleculeUnsaturation = (numCarbons) – [(numHydrogens)/2] – [(numHalogens)/2] + [(numNitrogens)/2] + 1*

(See the Figure below for examples.)

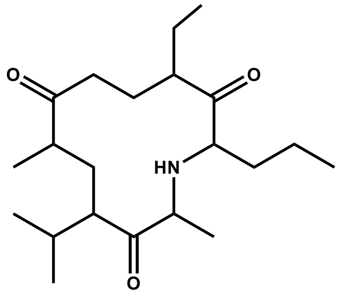

Chemical Formula: $C_{21}H_{37}NO_3$

C = 21
H = 37
Hal = 0
N = 1

DOU = (21) - (37/2) - (0/2) + (1/2) + 1
DOU = 4

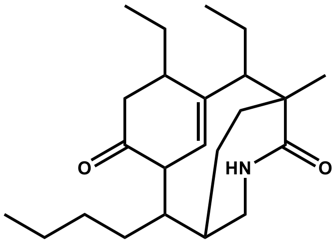

Chemical Formula: $C_{23}H_{37}NO_2$

C = 23
H = 37
Hal = 0
N = 1

DOU = (25) - (37/2) - (0/2) + (1/2) + 1
DOU = 6

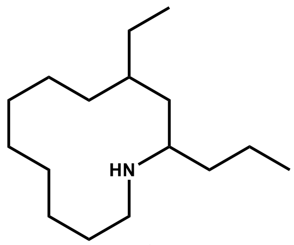

Chemical Formula: $C_{16}H_{33}N$

C = 16
H = 33
Hal = 0
N = 1

DOU = (16) - (33/2) - (0/2) + (1/2) + 1
DOU = 1

63. **Peripheral Carbonyls**: Count of carbonyls directly attached to the main macrocycle ring.
Counted using an algorithm which cycles through all peripheral groups; if the MC ring atom is a carbon AND the peripheral atom is an oxygen AND the two are double bonded to one another, it counts a carbonyl.

64. **Substituent Fsp3:** Fraction of substituent carbons that are sp^3 hybridized, defined according to Lovering *et al.* [5].
Substituent sp3 carbons are counted using an algorithm that cycles through each substituent atom and counts each tetrahedral carbon using the method getHybridizationState() from class MolAtom. Substituent Fsp3 is further calculated using the equation:
substituentFsp3 = (Substituent sp3 carbons)/(numSubstituentCarbons)

65. **Macrocycle Fsp3:** Fraction of carbons in the main macrocycle ring that are sp^3 hybridized, defined according to Lovering *et al.* [5].
MC sp3 carbons are counted using an algorithm that cycles through each MC atom and counts each tetrahedral carbon using the method getHybridizationState() from class MolAtom. Macrocycle Fsp3 is further calculated using the equation:
macrocycleFsp3 = (MC sp3 carbons)/N

66. **Molecule Fsp3:** Fraction of carbon atoms in the compound that are sp^3 hybridized, defined according to Lovering *et al.*[5].
Directly obtained from the method getFsp3() from the class TopologyAnalyserPlugin

67. **Maximum Substituent Size:** The number of heavy atoms in the largest substituent as measured by heavy atom count (see Figure below).
Identified using an algorithm that cycles through the substituents and identifies the largest one.

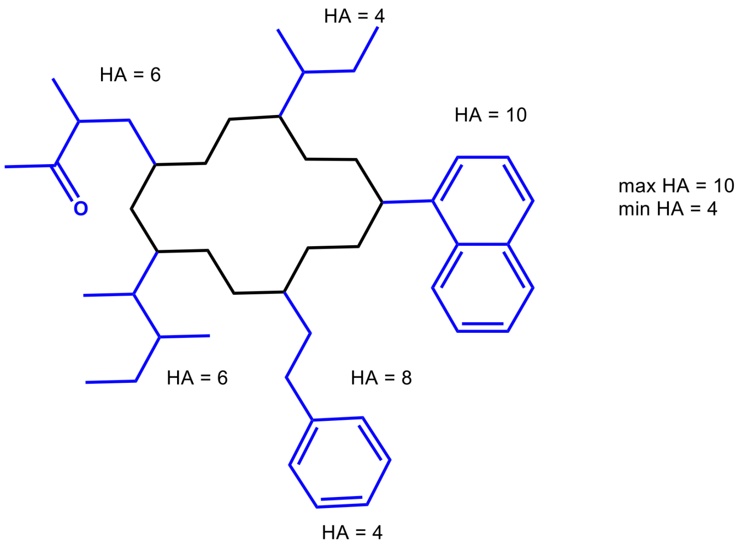

68. **Minimum Substituent Size:** The number of heavy atoms in the smallest substituent as measured by heavy atom count (see Figure below MolD_67).
    Identified using an algorithm that cycles through the substituents and identifies the smallest one.

69. **Mean Substituent Size:** Sum of all substituent heavy atoms divided by the number of substituents (see Figure below).
    All sizes checked from prior algorithm are summed and divided by the number of substituents to get the average.
    avgSubSize = (subSizeTotalSum)/numSubstituentGroups

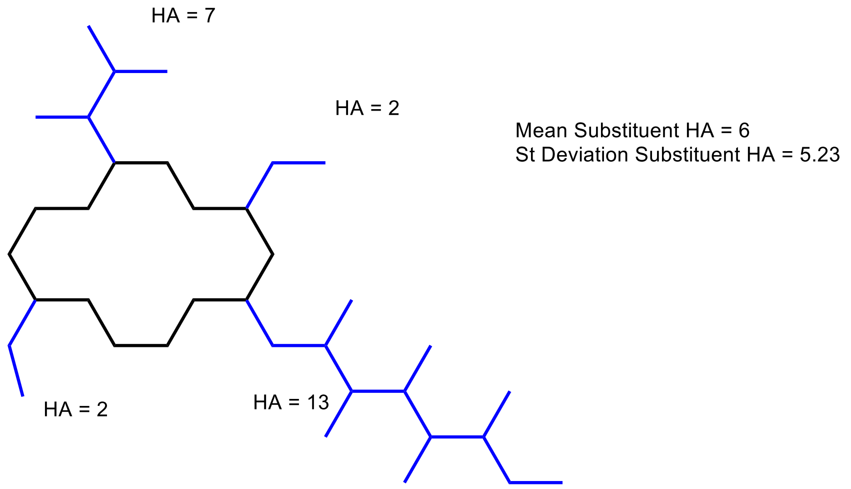

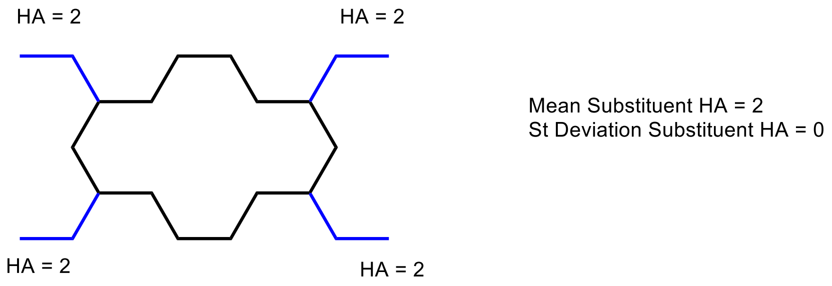

70. **Substituent Size Standard Deviation (Substituent Size st. dev):** The standard deviation of the substituent sizes as measured by heavy atom count (see Figure below MolD_69). A large standard deviation equates to a macrocycle containing substituents with a wide variety of sizes; a small standard deviation means the substituents are similar in size. A value of 0 means all substituents are of equal size.
    Population standard deviations for substituent sizes (stDevSubSize) is calculated using the substituent size values as gathered in the algorithm above, and their mean value.

71. **Substituent Size Distribution:** Distribution of substituent sizes relative to the mean substituent size (i.e. MolD_70 divided by MolD_69). A value of zero indicates all substituents are identical in size; a value greater than 1 indicates a wide diversity of substituent sizes (see Figure below for examples).

Calculated using values derived above with the equation:

subSizeDist = stDevSubSize / avgSubSize

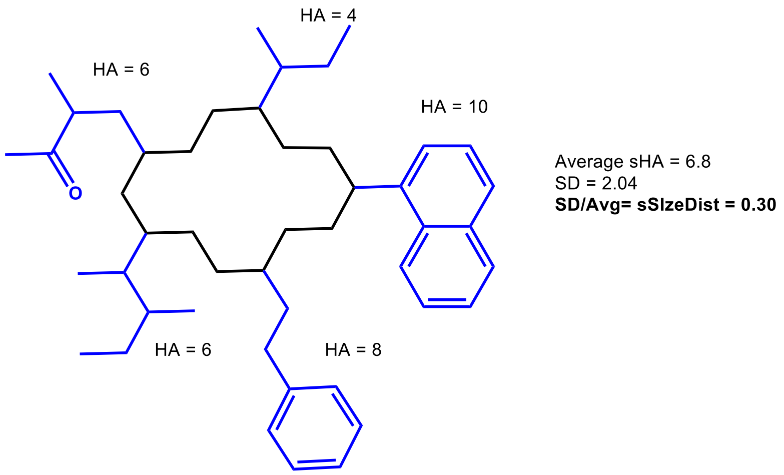

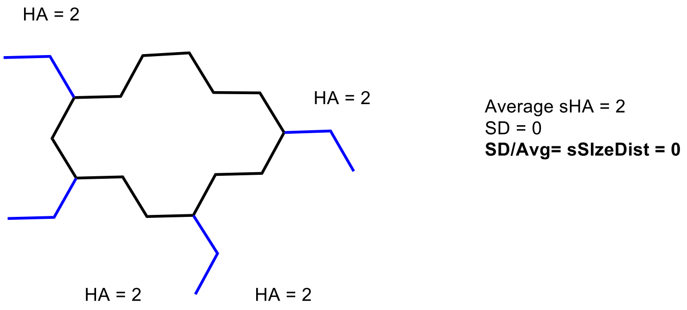

72. **Maximum Gap Size between Substituents (Max Gap Size):** The longest contiguous stretch of atoms in the main macrocycle ring between substituent connection points (see Figure below MolD_75 for examples). For clarity, peripheral groups are not considered when calculating gaps. Gap sizes are measured in terms of the number of MC ring atoms, not the number of MC ring bonds so, for example, the gap size between two substituents connected to adjacent atoms on the MC ring would be zero. If the maximum gap size equals the MC ring size, *N*, there are no substituents present. Bridging atoms are not counted as gaps (see MolD_37 and accompanying Figure for definition of bridging atoms).

Max Gap Size is identified using a gap analysis algorithm which (1) classifies each MC atom as a non-fused substituent connectivity point (S), a fused substituent connectivity point (F), a bridging atom (B) or a gap atom (G), which includes both peripheral group connectivity points and the remaining MC atoms with no connections to heavy atoms outside the MC ring; (2) identifies the start of a gap sequence (S-G) as an ideal start point; and (3) traverses the ring in sequence, counting the size of all gaps between

substituents, and also identifying gaps of size 0 (S-S, S-F, or F-S). Distances between two fused substituent connectivity points (F-F) or (F-B_x-F) are not counted.

73. **Minimum Gap Size between Substituents (Min Gap Size):** The shortest contiguous stretch of atoms in the main macrocycle ring between substituent connection points, measured in terms of the number of MC ring atoms (see MolD_72 for fuller description, and Figure below MolD_75 for examples). If the compound contains substituents connected to adjacent atoms on the MC ring, Min Gap Size will equal zero. If the minimum gap size equals the MC ring size, *N*, there are no substituents present.
    Min Gap Size is identified using the gap analysis algorithm described above (72).

74. **Average Gap Size between Substituents (Mean Gap Size):** The mean gap size, where gaps are defined as described in MolD_72 (see Figure below MolD_75 for examples).
    The Mean Gap Size is calculated during the gap analysis algorithm described above (72).

75. **Standard Deviation of Gap Sizes between Substituents (Gap Size st dev):** The population standard deviation of gap sizes, as described in MolD_72. A large standard deviation means the substituents' connection points are distributed irregularly around the macrocycle ring, whereas a zero standard deviation means the substituents are evenly spaced (see Figure below for examples).
    The standard deviation of all gap sizes is calculated during the gap analysis algorithm described above (72).

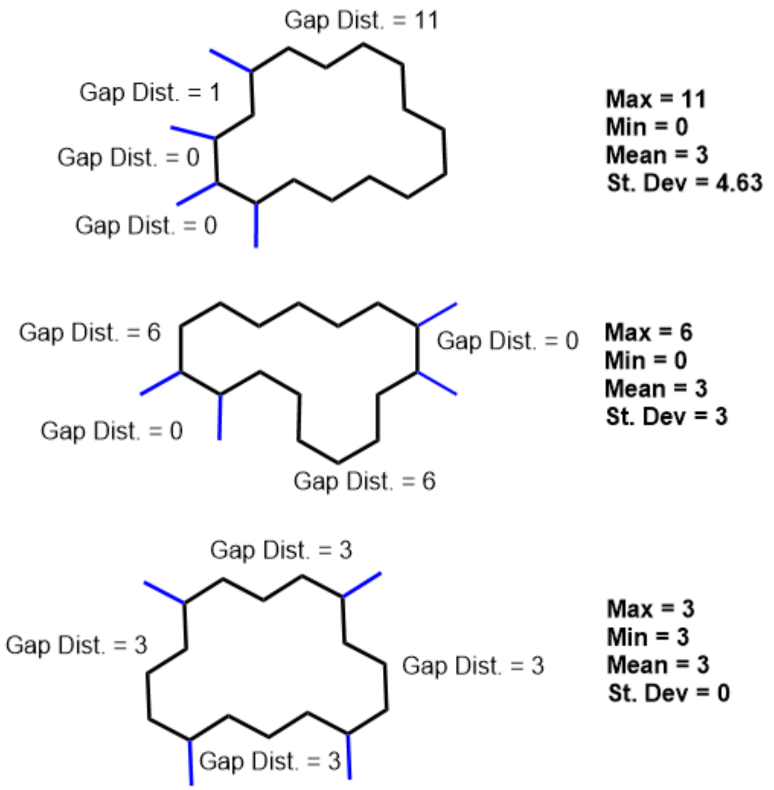

76. **Maximum Gap Size between Substituents, Normalized (Max Gap Size/N):** The maximum gap size, from MolD_72, normalized to the size of the macrocycle ring (see Figure below MolD_79 for examples). The resulting value represents the largest fraction of the main macrocycle ring circumference that is free of substituents.

    Calculated from values derived above using the equation:

    (Max Gap Size)/N

77. **Minimum Gap Size between Substituents, Normalized (Min Gap Size/N):** The minimum gap size, from MolD_73, normalized to the size of the macrocycle ring (see Figure below MolD_79 for examples). The resulting value represents the smallest fraction of the main macrocycle ring circumference that is free of substituents. If the compound contains substituents connected to adjacent atoms on the MC ring, both Min Gap Size and Min Gap Size/N will equal zero.

    Calculated from values derived above using the equation:

    (Min Gap Size)/N

78. **Average Gap Size between Substituents, Normalized (Mean Gap Size/N):** The minimum gap size, from MolD_74, normalized to the size of the macrocycle ring (see Figure below MolD_79 for examples). The resulting value is the average fraction of the main macrocycle ring circumference between substituents. A value of zero indicates a compound that is so densely decorated with substituents that every MC ring atom is a substituent connection point. A value of one indicates the complete absence of any substituents.

    Calculated from values derived above using the equation:

    (Mean Gap Size)/N

79. **Standard Deviation of Gap Sizes between Substituents, Normalized (Gap Size st dev/N):** The standard deviation of gap sizes, from MolD_75, normalized to the size of the macrocycle ring (see Figure below for examples). A value means the substituents are distributed unevenly around the main macrocycle ring; a value of 0 means the subsistent are evenly distribution distributed.

    Calculated from values derived above using the equation:

    (St. Dev of Gap Sizes)/N

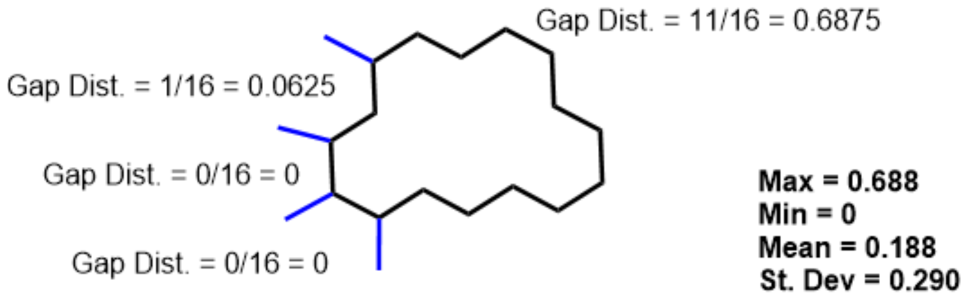

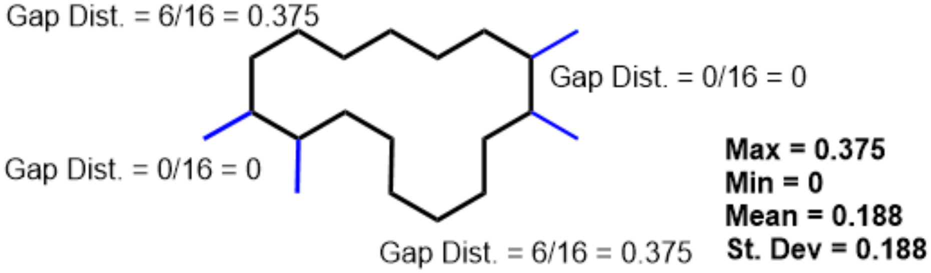

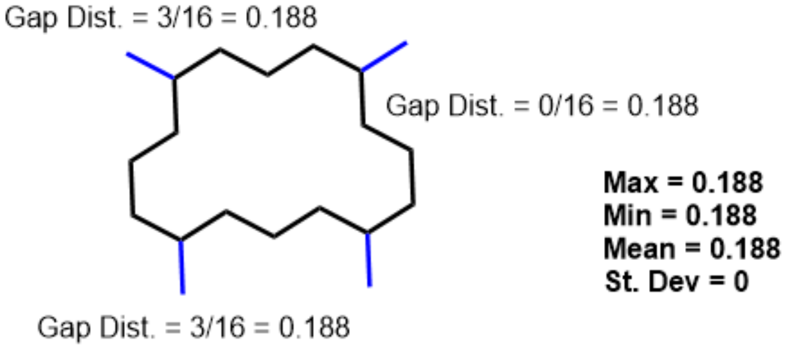

80. **Bridging atoms:** Count of how many atoms in the main macrocycle ring separate the attachment points of a connecting ring. The attachment points themselves do not count. Note, the shorter path between attachment points is considered the “bridge”. Thus, in the figure below the upper structure is considered as a 14-membered macrocycle (black) with two fused rings attached (blue) and a total of three bridging atoms (red). The lower structure is considered to be a 16-membered macrocycle, also with two fused rings attached, and with five bridging atoms.

The total number of bridging atoms (numBridgeAtoms) is alculated using an algorithm that cycles through each fused ring substituent (as identified above), calculating the longest and shortest path (in MC atoms) between the two MC atoms which serve as connection points for the fused substituent. If the shortest path between these connection points is zero atoms, there are no bridging atoms. If the shortest path between these connection points is one or more atom, each of these intervening atoms are flagged and counted as bridging atoms.

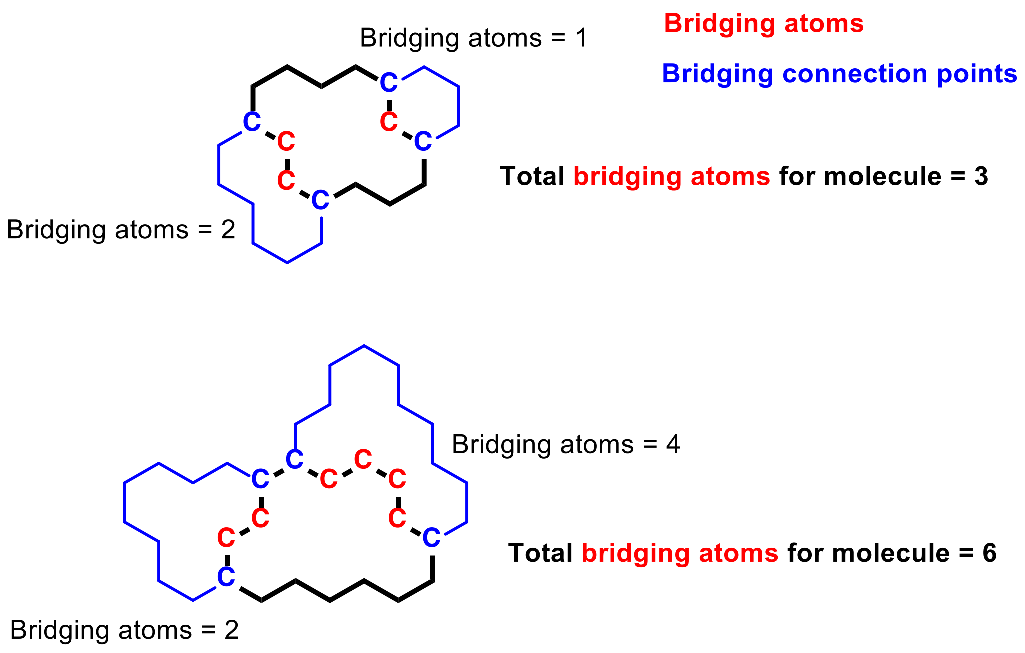

81. **Bridging atoms/N:** The number of bridging atoms, from MolD_80, normalized to the size of the main macrocycle ring (see Figure below).

Calculated from values derived above using the equation:

fractionBridgeAtoms = (numBridgeAtoms)/N

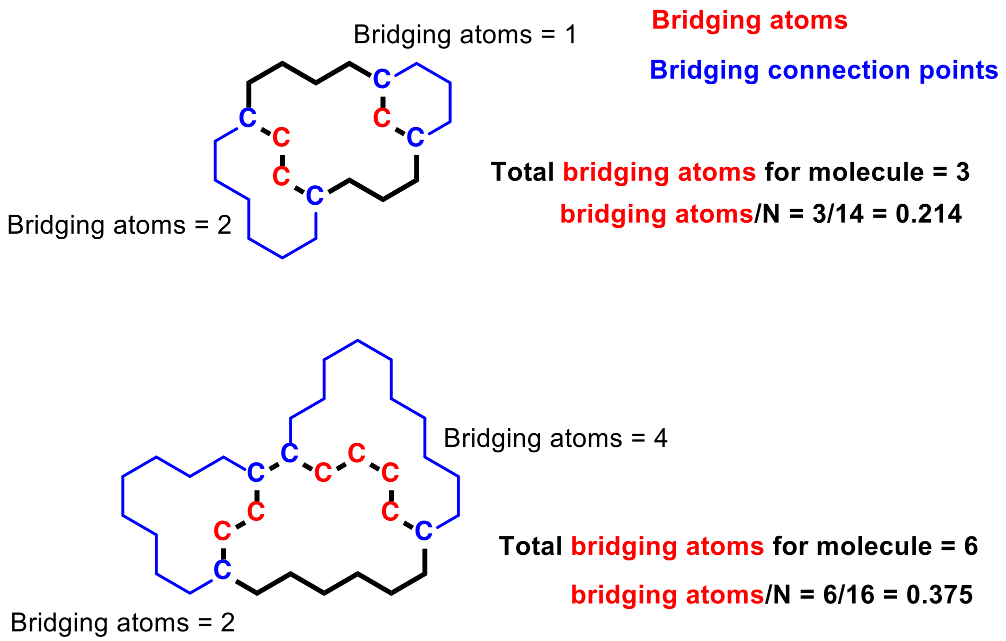

82. **Restricted Fraction (Restricted/N):** A count of the number of bonds in the main macrocycle ring that are in any way rigidified, as defined by a reduced freedom to oscillate about the dihedral angle relative to an unrestricted single bond within the ring. This count is

normalized to the size of the macrocycle ring. Restricted bonds include:

- Bonds that are part of a ring fusion
- Amide bonds
- Double, triple and aromatic bonds

Bonds espousing two or more of the above qualities are only counted once.

The restricted fraction (restrictedFraction) is identified using an algorithm which cycles through the MC bonds checking for bond order using the getBondType() method from the class TopologyAnalyserPlugin, amides flagged during the algorithm described below (84) and fusions flagged during the algorithms described above (36).

See the Figure below for examples (restricted bonds are colored red).

restrictedFraction = (restrictedCount)/N

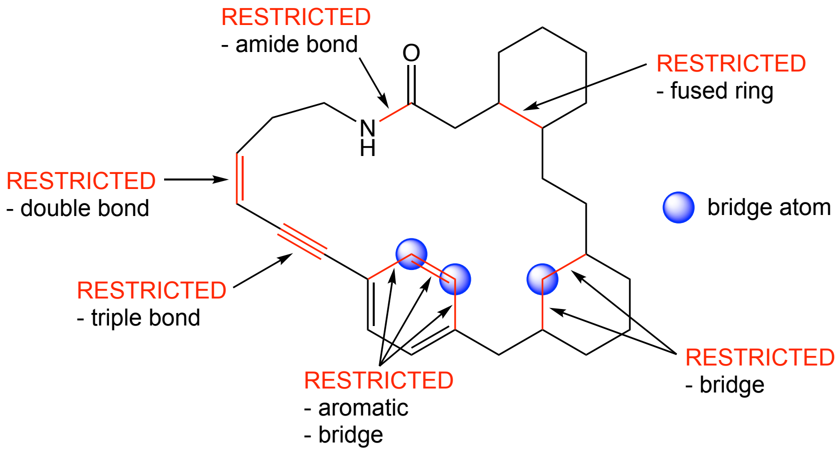

82. **Non-restricted Fraction (Non-restricted/N):** A count of the number of bonds in the main macrocycle ring that have less-restricted ability to oscillate about the dihedral angle, normalized to the size of the macrocycle ring. This descriptor is the inverse of MolD_82, such that, for any macrocycle, MolD_82 + MolD_83 = 1. Unrestricted bonds are any bonds in the main macrocycle ring that are single bonds, which are neither part of a ring fusion, nor amide bonds.

The non-restricted fraction (unrestrictedFraction) is identified using an algorithm which cycles through the MC bonds analyzing bond order using the getBondType() method from the class TopologyAnalyserPlugin, with a cross-check for whether any identified single bonds are amides or part of fusions.

- See the Figure above for examples (unrestricted bonds are the bonds in the main macrocycle ring that are <u>not</u> colored red).

unrestrictedFraction = (unrestrictedCount)/N

84. **MC Amide Bonds:** A count of amide bonds in the main macrocycle ring.

The number of MC amide bonds (ringAmideCount) is calculated using an algorithm that cycles through the MC bonds, looking for N-C bonds and C-N bonds. In either case, it checks if the "C" involved is a carbonyl, and if so it flags the bond and counts it as an

amide. The sum is reported here, and the "amide" flag is further used in restricted/unrestricted fraction algorithms described above.

85. **Ring Complexity without heteroatoms (rComplx):** Fraction of valences of carbon atoms in the main macrocycle ring that bonded to a substituent, a peripheral group, or involved in a π-bond with an adjacent MC ring atom, as a measure of how densely the main macrocycle ring is decorated. Each carbon atom in the MC ring can engage in two bonds in addition to the minimum of two sigma bonds that connect it to its neighboring MC ring atoms. The complexity of decoration can therefore be quantified as the number of MC ring carbon valences that are not bonded to hydrogen atoms, divided by the total number of ring carbon valences (= 2x the number of ring carbons), as per the following equation:

$$rComplx = \frac{Ring\ carbons\ valances\ not\ bonded\ to\ H}{Total\ ring\ carbon\ valances} = \frac{2rC - rCH}{2rC}$$

Where rC is the number of carbon atoms in the main macrocycle ring, and rCH is the number of hydrogen atoms directly bonded to ring carbons (identified using standard connectivity methods)

See Figure below for examples.

Calculated from values derived above with the equation:
*ringComplexityWithout = ((2 * numMCcarbons) - numMCcarbonHbonds) / (2 * numMCcarbons)*

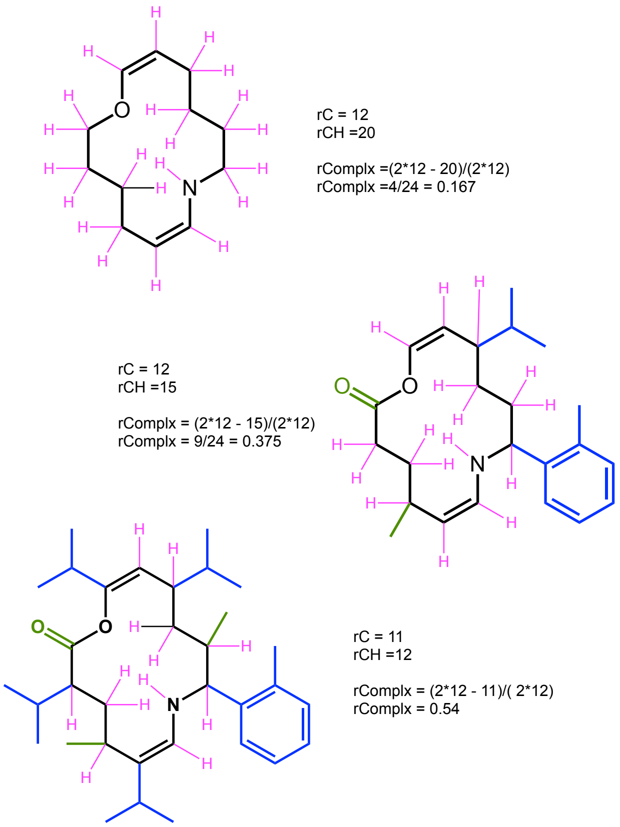

86. **Ring Complexity with heteroatoms (rComplxHet)**: Fraction of valences of all atoms in the main macrocycle ring that bonded to a substituent, a peripheral group, or involved in a π-bond with an adjacent ring atom, as a measure of how densely the main macrocycle ring is decorated. Differs from MolD_85 in including ring nitrogen and sulfur atoms as potential attachment points for substituents or peripheral groups, or as potential partners in π-bonds with other ring atoms. Each carbon atom in the ring can form two bonds in addition to the minimum of two sigma bonds that connect it to its neighboring ring atoms. Each nitrogen atom in the ring can form up to one such additional bond. We considered that each sulfur atoms in the ring might form up to two additional bonds (with oxygen, e.g. in a sulfoxide or a sulfone). The complexity of decoration, taking all of these possible attachment points on all atoms of the main macrocycle ring, can therefore be quantified as the number of ring carbon and nitrogen valences that are not bonded to hydrogen atoms, plus the number of S=O bonds involving ring sulfur atoms, all divided by the total number of ring atom valences (= 2x the number of ring carbons + 1x the number of ring nitrogens, plus 2x the number of ring sulfurs), as per the following equation:

$$rComplxHet = \frac{2(rC) - rCH + rN - rNH + r(S = O)}{2(rC + rS) + rN}$$

Where rC is the number of carbon atoms in the main macrocycle ring, ring carbons, rN is the number of ring nitrogens, rCH is the number of MC ring CH bonds, rNH is the mumber of MC ring NH bonds, and r(S=O) is the number of MC ring S=O bonds. C-H, N-H and S=O bonds to MC atoms are identified using standard connectivity methods.

Calculated from values derived above with the equation:

*ringComplexityWith = [((2 * (numMCcarbons)) - numMCcarbonHbonds) + (numMCnitrogens - numMCnitrogenHbonds) + (numMCsulfurObonds))] / [((2 * numMCcarbons) + numMCnitrogens + (2 * numMCsulfurs)]*

87. **Substituent Flexibility Fraction (sF):** Count of the number of rotatable bonds in the substituents normalized to the cumulative size of the substituents in terms of number of heavy atoms. A measure of overall substituent flexibility. Bonds in a ring are not considered rotatable, nor are bonds to a terminal heavy atom (which includes all peripheral groups). Therefore, all rotatable bonds in the molecule are in the substituents. Substituent Flexibility Fraction is therefore defined as NRB for the molecule, from MolD_7 (which equals the number of rotatable bonds in the substituents), divided by the total number of heavy atoms in all substituents combined.

Calculated from values derived above with the equation:

subFlexibility = ((numRotatableBonds)+1)/(HA-N-numPeripheralAtoms+1)

88. **Substituent Rotation (sRot):** Count of how many substituents are connected to the main macrocycle ring in a rigid or non-rotatable manner, *via* a double bond, a spiro ring, or a fused ring. (See Figure below for examples; rigid connections are shown in red and rotatable connections in green.)

The number of substituents connected to the MC with a double bond (subConnectionDoubleBond) are counted using an algorithm that cycles through non-fusion substituents, and checks the bond order of the substituent-to-MC bond using the getBondType() method from the class TopologyAnalyserPlugin. Fused and spiro substituents are calculated as described above, and plugged into the equation:

subRotation = subConnectionDoubleBond + numNonSpiroFusedRings + numSpiroRings

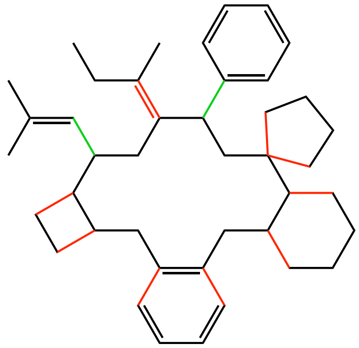

89. **Peptide Character Index:** The proportion of bonds in the main macrocycle ring that are amide bonds, as a fraction of the maximum possible number of amide bonds in a ring of the same size, to quantify how peptidic the macrocycle ring is. The value is calculated by counting the number of amide bonds in the main macrocycle ring, and dividing this number by one third of the total number of macrocycle ring bonds (to account for the fact that a pure cyclic peptide has one amide bond per every three ring bonds). If the macrocycle ring were completely peptidic, it would have a Peptide Character Index equal to 1, while a compound with no amide bonds in the main macrocycle ring will have Peptide Character Index of 0.

Calculated from values derived above using the equation:

*peptideCharacterIndex = (ringAmideCount)/(N*0.333)*

90. **Charges:** A count of (nonionizable) charged atoms in the molecule.

The total count of charges (numCharges) is calculated using an algorithm which cycles through all atoms in the molecule and analyzes charges using the method getCharge() from class MolAtom. A running sum is tallied of the absolute value of the charge on each atom. A -1 charge is worth 1, a +1 charge is worth 1, a -2 charge is worth 2, etc.

References:

1. Downs, G. M., Gillet, V. J., Holliday, J. D. & Lynch, M. F. Review of Ring Perception Algorithms for Chemical Graphs. *J. Chem. Inf. Comput. Sci.* **29**, 172–187 (1989).
2. Lipinski, C. A., Lombardo, F., Dominy, B. W. & Feeney, P. J. Experimental and computational approaches to estimate solubility and permeability in drug discovery and development settings. *Adv. Drug Deliv. Rev.* **64**, 4–17 (1997).
3. Veber, D. F. *et al.* Molecular properties that influence the oral bioavailability of drug candidates. *J. Med. Chem.* **47**, 2615–2623 (2002).
4. Villar, E. A. *et al.* How proteins bind macrocycles. *Nat. Chem. Biol.* **10**, 723–731 (2014).
5. Lovering, F., Bikker, J. & Humblet, C. Escape from flatland: Increasing saturation as an approach to improving clinical success. *J. Med. Chem.* **52**, 6752–6756 (2009).
