# Supplementary material for: Defining and navigating macrocycle chemical space
Source: Chem Sci. 2021 Mar 4;12(12):4309–28. doi: 10.1039/d0sc05788f (PMC8179434; doi:10.1039/d0sc05788f)
Supplement: SC-012-D0SC05788F-s003 [file SC-012-D0SC05788F-s003.pdf]

Electronic Supplementary Material (ESI) for Chemical Science.
This journal is © The Royal Society of Chemistry 2021

Supplemental Information

Defining and Navigating Macrocycle Chemical Space

Lauren A. Viarengo-Baker[a], Lauren E. Brown[a,b], Anna A. Rzepiela[c], and Adrian Whitty[a,b]*

[a]Department of Chemistry, Boston University, 590 Commonwealth Ave, Boston, Massachusetts 02215, USA.[b]Center for Molecular Discovery, Boston University, 24 Cummington Mall, Boston, Massachusetts 02215, USA. [c]Pyxis Discovery, Delftechpark 26, 2628XH, Delft, The Netherlands

Address correspondence to: Adrian Whitty, whitty@bu.edu.

Supplemental Figures

Figure S1. PCA analysis of all MC sets using only the Lipinski and Veber properties versus using all 90 MDs.

Figure S2. MC Chemical Space as defined by PCA of all compound sets using just the Lipinski and Veber descriptors versus using all 90 MDs.

Figure S3. Oral and non-oral MC drugs and clinical candidates plotted with respect to PCs 1-3, with zone designations.

Figure S4. All MC sets mapped onto MC chemical Space with respect to PC1 and PC2, with the oral MC drug zones overlaid.

Figure S5. Locations of hypothetical compound designs plotted with respect to PCs 1-10.

Figure S6. Flow scheme illustrating how the themes underlying PCs 1 and 2 were used to guide the redesign of a starting MC structure to identify a new chemotype that resides in a target region of MC structure-property space.

Figure S7. Histogram showing the distributions of values, for the oral drugs and clinical candidates, of the 13 most important properties used to define MC oral drug space.

Figure S8. Distribution of the number of property violations for each MC set.

Figure S9. Correlation between the number of key property violations and the distance of individual compounds from the center of Zone 1 calculated using 10 PCs versus 7.

Figure S10. Historical examples of MC optimization efforts mapped onto MC Chemical Space.

Supplemental Tables

Table S1. Oral MC Drugs and Clinical Candidates with PCA scores, and zone designations.

Table S2. Non-Oral MC Drugs and Clinical Candidates with PCA scores.

Table S3. Property ranges and minimum and standard deviation values used to normalize the descriptor values, and the PCA coefficients calculated for each descriptor upon analysis of the collection of MC compound sets.

Supplemental Tables, cont'd

Table S4. Molecular descriptors with the largest (absolute) coefficient values for PC1 and PC2.

Table S5. Distance of each compound design from Figure 6 from the center of mass of the Oral MC Drugs set.

Additional supplemental information supplied as separate files:

Supplemental Documents

Supplemental Document A: Detailed description of molecular descriptors used, with pseudocode.
Supplemental Document B: Excel file containing SMILES and descriptor values for the 42 representative examples used in the PCA analysis from each of (i) the Oral MC Drug Set, (ii) the Non-Oral MC Drug Set, and (iii) Set A (synthetic MCs from the BU-CMD collection).

Supplemental Videos

Video A: **A 3D view of macrocycle chemical space.** MC compound sets, color-coded as in the main figures, are plotted with respect to their scores in Principal Components 1-3. The correspondingly colored ellipsoids show the volume of chemical space (in the first three PCs) encompassed by each MC set, calculated as described in the main text. The wireframe ellipsoid shows the volume of chemical space defined by the combined MC sets.

Video B: **A 3D view of the oral macrocycle drugs and clinical candidates colored with respect to the zones they occupy in MC structure-property space.** The 42 oral MC drugs and clinical candidates plotted with respect to their scores in Principal Components 1-3. The compounds are colored as follows: blue, compounds in Zone 1; red, compounds in Zone 2; yellow, compounds in Zone 3; black, other compounds.

Video C: **A 3D view of the non-oral macrocycle drugs and clinical candidates colored with respect to the zones they occupy in MC structure-property space.** The 52 non-oral MC drugs and clinical candidates plotted with respect to their scores in Principal Components 1-3. The compounds are colored as follows: blue, compounds in Zone 1; yellow, compounds in Zone 3; orange, compounds in Zone 3A; black, other compounds. No non-oral drugs or clinical candidates reside in Zone 2.

Figure S1

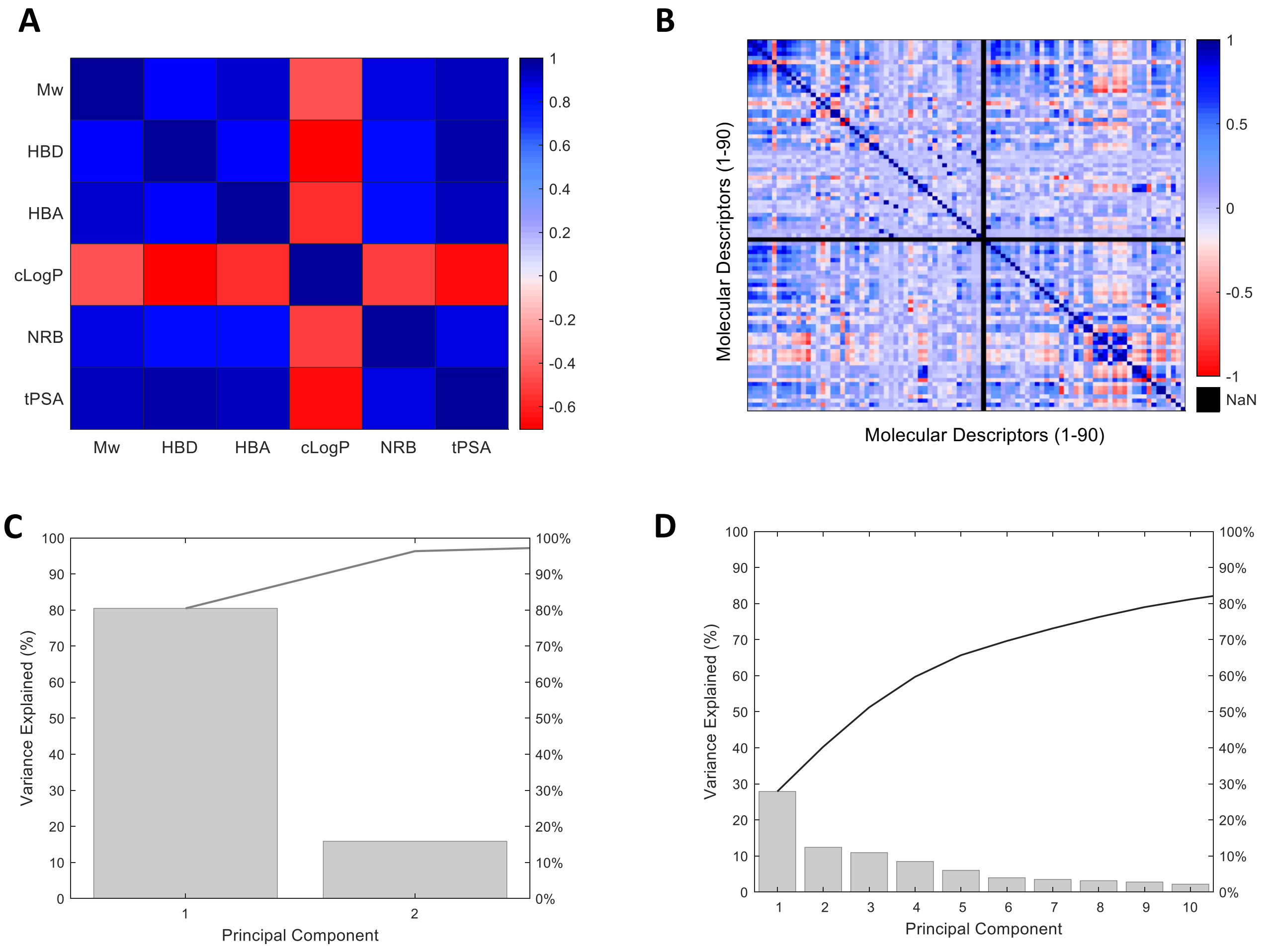

Figure S1. PCA analysis of all MC sets using only the Lipinski and Veber properties (left panels) or all 90 MolDs (right panels). (A,B) Covariance matrix for all properties in each analysis showing that, across all MC sets, the Lipinski and Veber properties are strongly covariant, while much less covariance is seem among the full panel of 90 MolDs. **(C,D)** Percent variance explained by each PC, showing that when only the Lipinski and Veber properties are used for PCA **(C)** the first PC alone explains ~80% of the variance in the data. In contrast, when all 90 MolDs are used **(D)**, 10 PCs are required to explain ~80% of the variance, indicating that including the additional descriptors adds nonredundant information to the analysis.

Figure S2

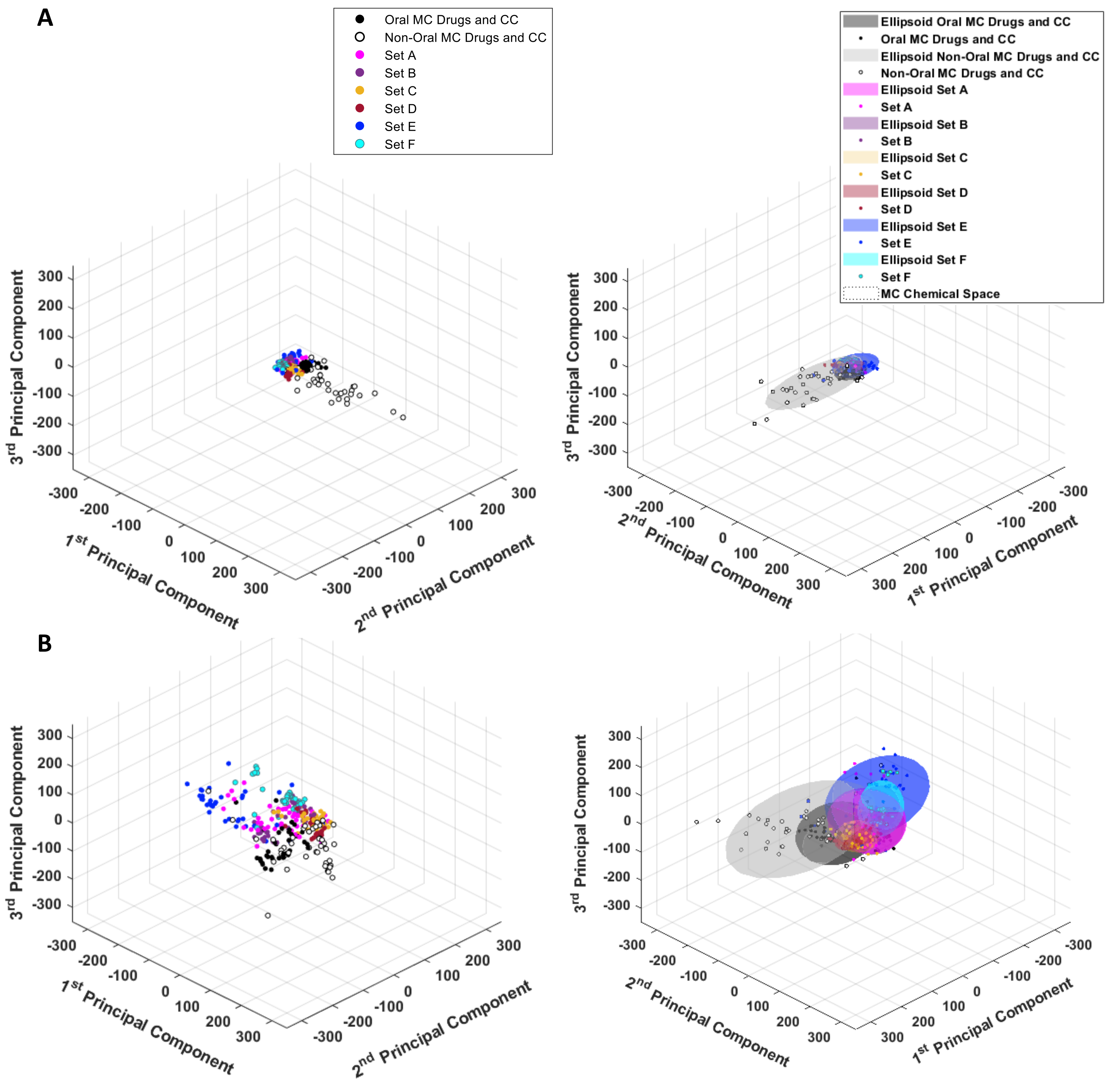

Figure S2. MC Chemical Space as defined by PCA of all compound sets using just the Lipinski and Veber descriptors (A) or all 90 MolDs (B). Left hand plots: The coordinates (scores) of the representative compounds from each MC set plotted with respect to PCs 1-3. Right hand plots: The region of structure-property space occupied by each MC set, represented as ellipsoids that encompass the middle 95% of compounds with respect to each PC axis.

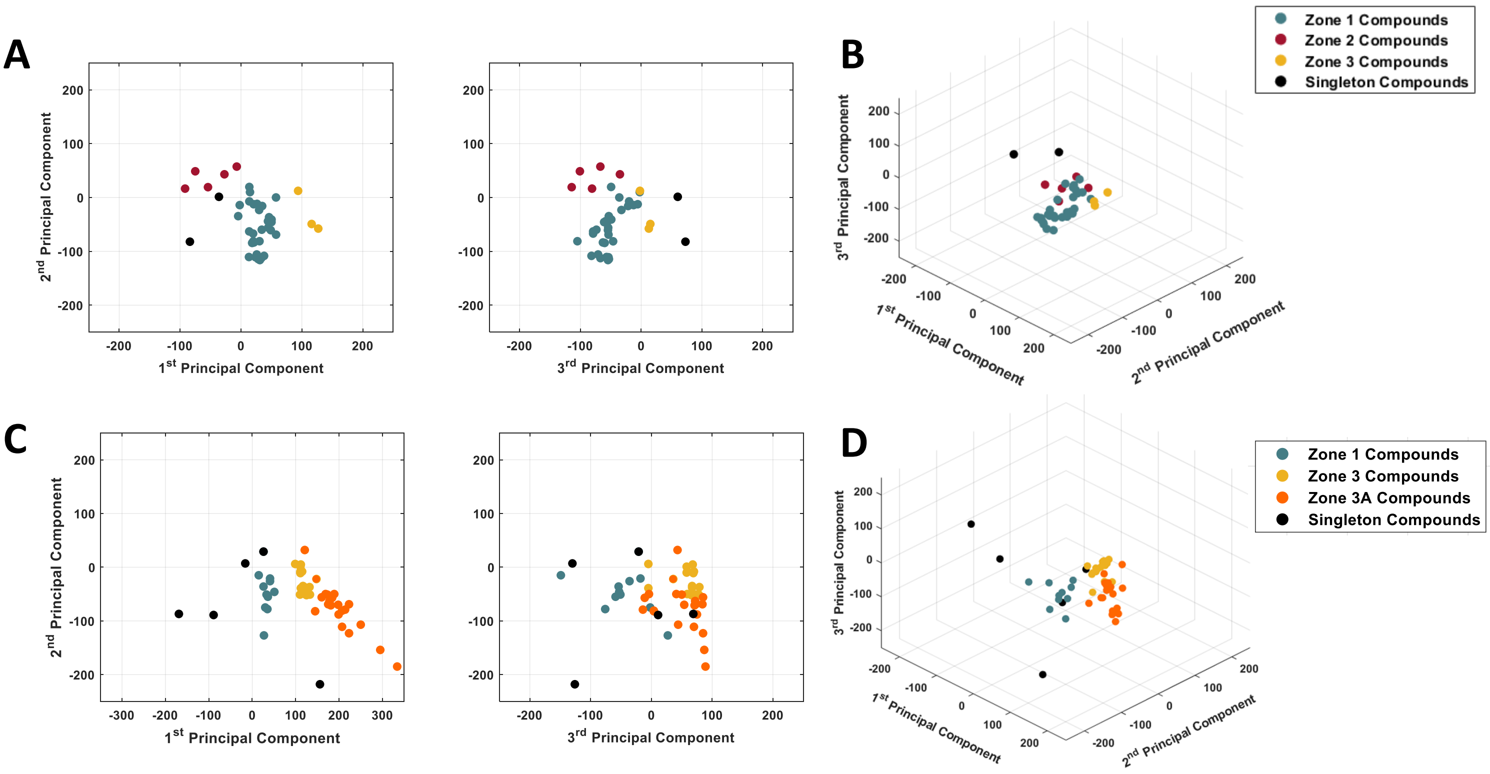

Figure S3. Oral and non-oral MC drugs and clinical candidates plotted with respect to PCs 1-3 with zone designations. PC1 versus PC2, and PC3 versus PC2 2-D PCA plots of (A) Oral MC drugs and clinical candidates and (C) non-oral MC drugs and clinical candidates. Data are colored with respect to zone classification (as shown in **Figure 3, Tables S1**). 3-D plot of (B) oral and (D) non-oral MC drugs and clinical candidates with respect to zone classifications (as shown in **Figure 3, Tables S1**). Movies of 3-D rotation of panels B and D are provided as supplementary material (**Supplementary Videos B, C**).

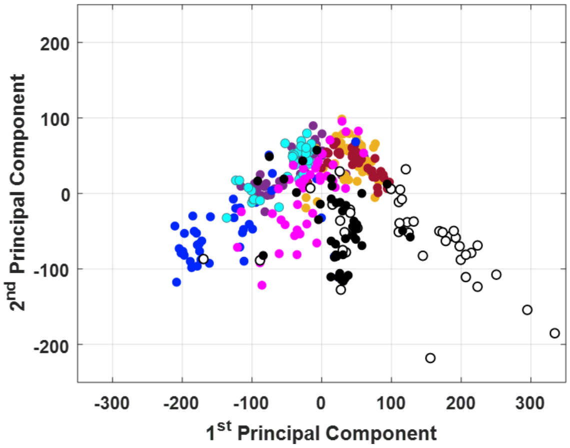

Figure S4. View of MC Chemical Space in PC1 and PC2 with the oral MC drug zones overlaid. (A) Zones from **Figure 3** (Zone 1, turquoise and dark blue; Zone 2, red; Zone 3, yellow) are overlaid on the view of MC chemical space from the perspective of PC1 versus PC2, as shown in **Figure 2D**, showing how the compounds (colored as designated in **Figure 2**) are distributed in MC chemical space with respect to the oral MC drug zones. Very few synthetic compounds lie in Zone 1.

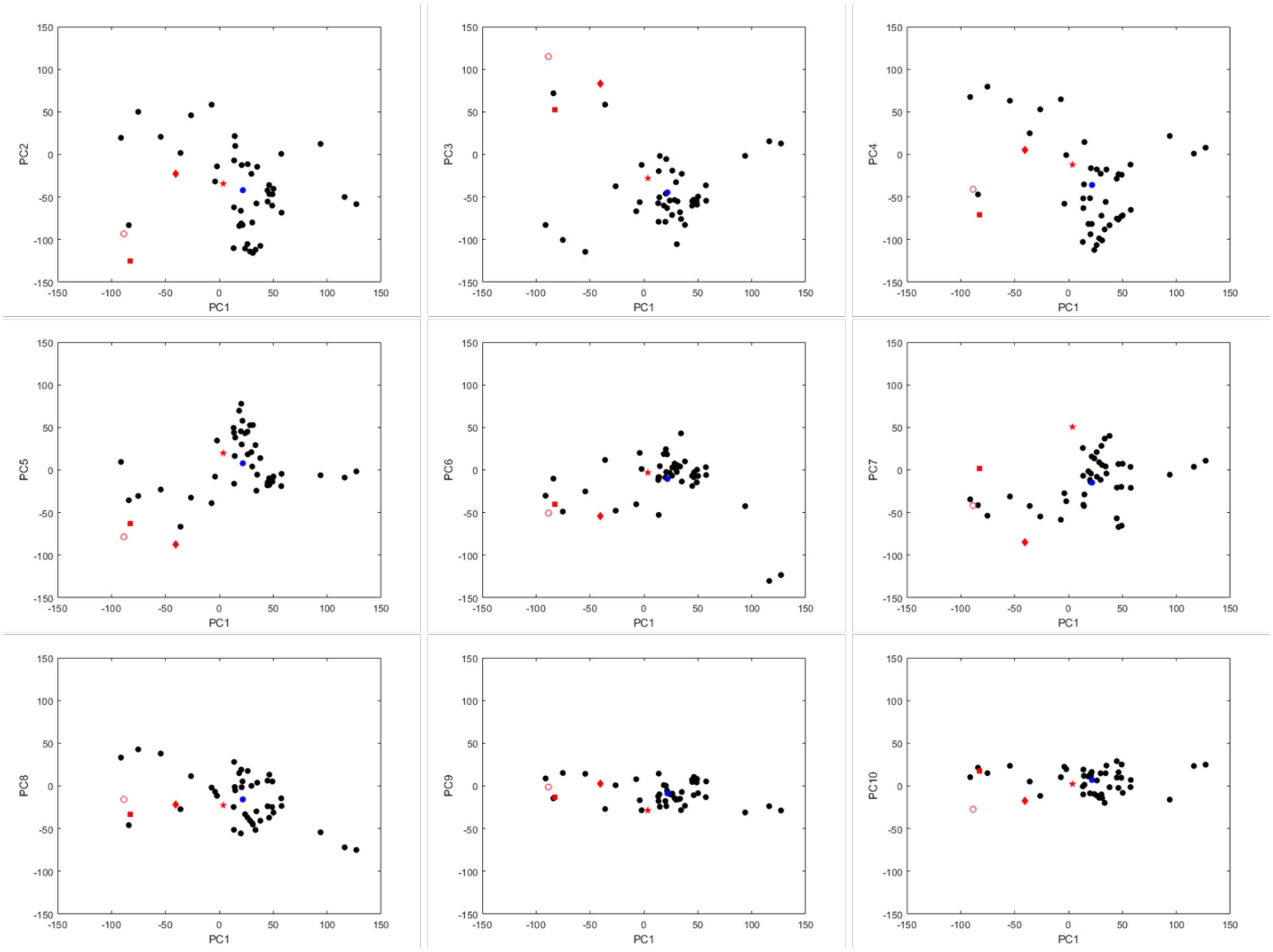

Figure S5. Locations of the compound designs from Figure 6 (main text), plotted with respect to PCs 1-10. Starting compound **1** is shown as a red open circle, the Oral MC drugs and clinical candidates are shown as black filled circles. The locations of the compound designs **2** (red lozenge) and **3** (red star), derived by redesigining **1** according to the structural themes deduced from PCA, are shown. Compound **4** (red square) was designed without reference to the PC themes. The results show that the design **2** lies slightly closer to the center of mass of the oral MC drugs and clinical candidates (blue asterisk), considered in all 10-dimensions, while compound **3** is close to this target location.

Figure S6

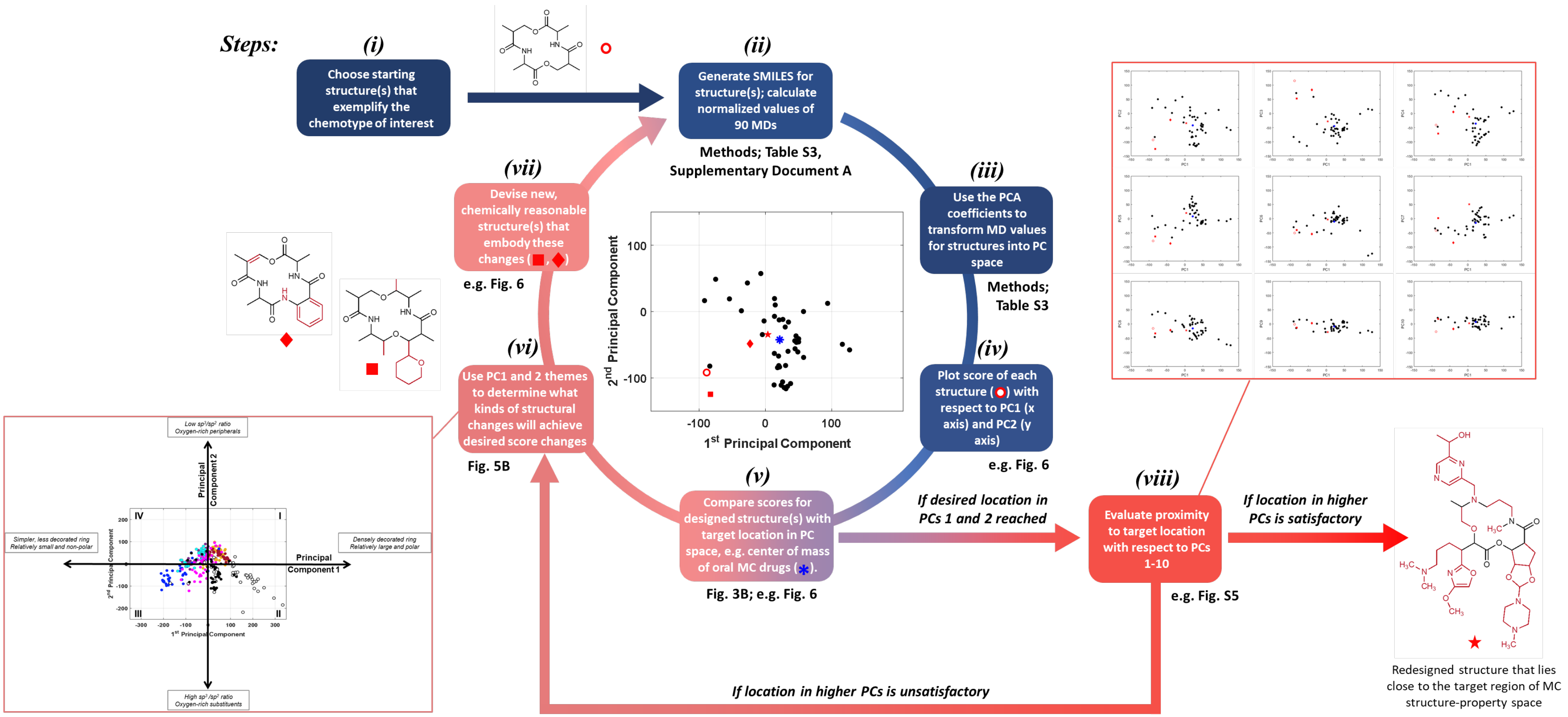

Figure S6. Flow scheme illustrating how the themes underlying PCs 1 and 2 (Figure 5) were used to guide the redesign of a starting MC structure to identify a new chemotype that resides in a target region of MC structure-property space (Figure 6). In the example shown, the tyarget region os the center of mass of the oral MC drugs and clinical candidates (blue asterisk). The same procedure could be used to target any other desired region of MC property space, such as Zone 2 for a kinase inhibitor or an unexplored region. Reference information, tables, and figures needed for the procedure are annotated below each step.

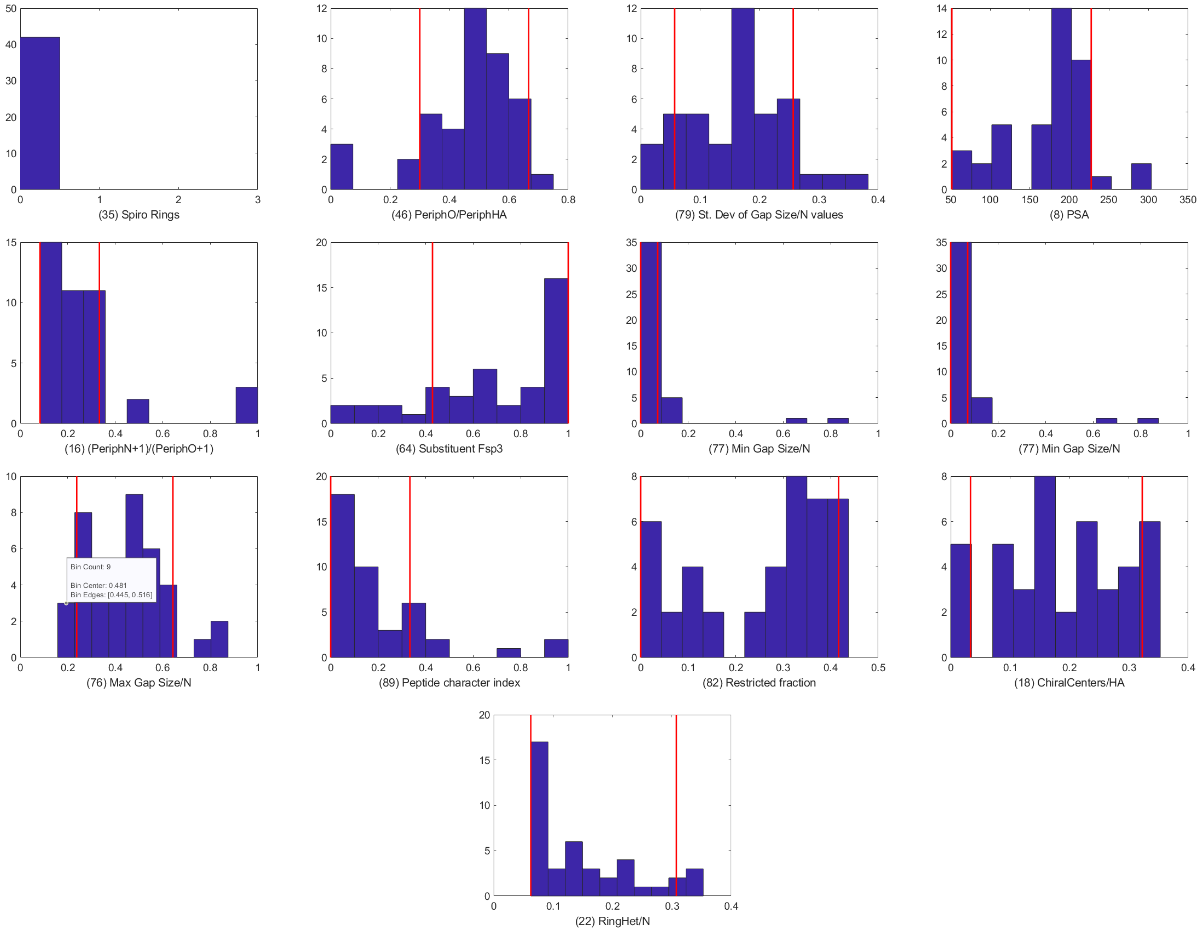

Figure S7. Histogram of 13 most important properties (from Table 2, main text) used to define MC oral drug space. Vertical red lines indicate range thresholds that include 80% of the compounds (as detailed in materials and methods); No range thresholds are given for MolD 35, Spiro Rings, which had no distribution.

Figure S8

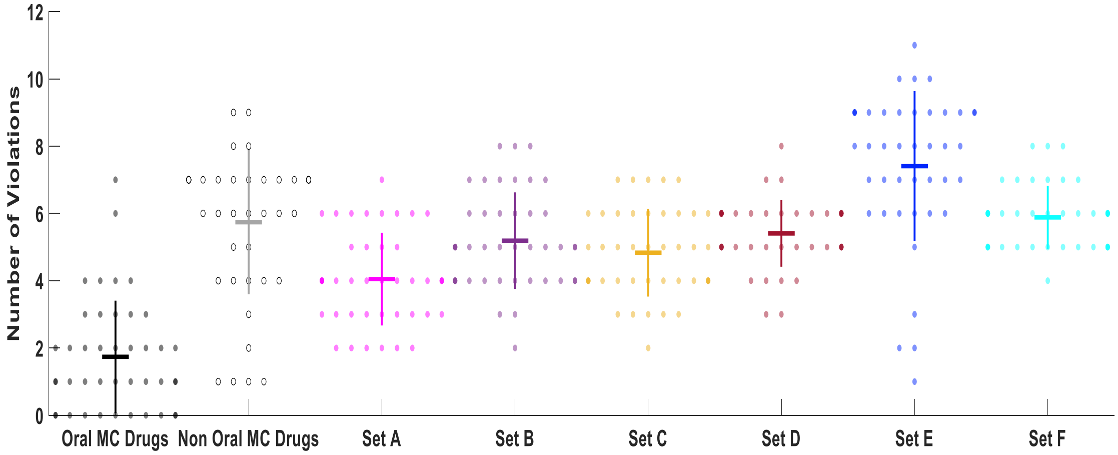

Figure S8. Distribution of the number of violations of the key property value ranges for each compound set. Beeswarm plot showing the distributions, means (horizontal lines), and interquartile ranges (vertical lines), of the number of violations for each MC set with respect to the property ranges listed in Table 2 of the main text. Compound sets are colored as in **Figures 2, 4** and **5** of the main text.

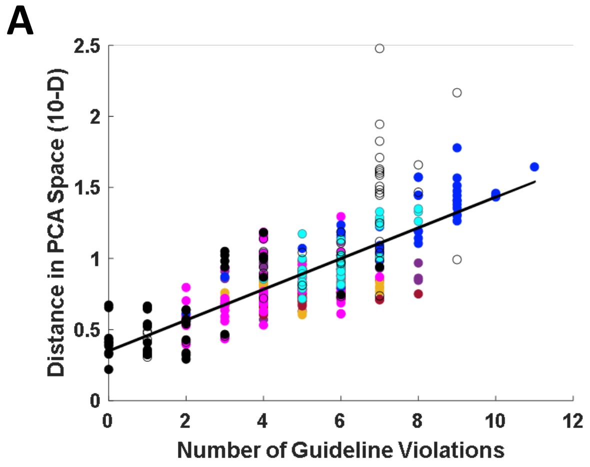

Figure S9. Correlation between the number of violations and the distance of individual compounds from the center of Zone 1 calculated using 10 PCs versus 7. The distance of each compound, in (A) 10-dimensional PC space (R^2 = 0.55), or (B) 7-dimensional PC space (R^2 = 0.53), from the center of the Oral MC Drug set, plotted against the number of property violations, for the 42 representative compounds from each set. Compound sets are colored as in **Figures 2, 4** and **5** of the main text. Panel A is identical to **Figure 7D** in the main text. The distance is expressed in the units of PC space, normalized by dividing by the range of values in PC1 for all compounds.

Figure S10

A

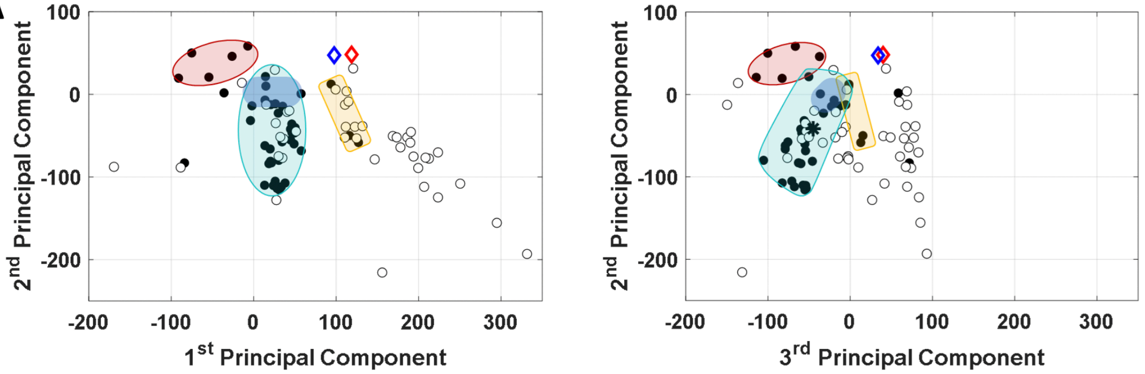

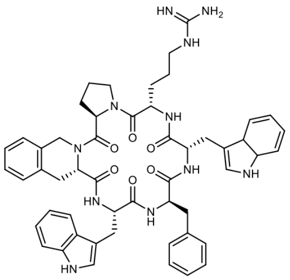

3 ◊

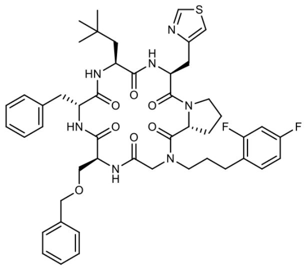

25 *(%F = 18)* ◊

B

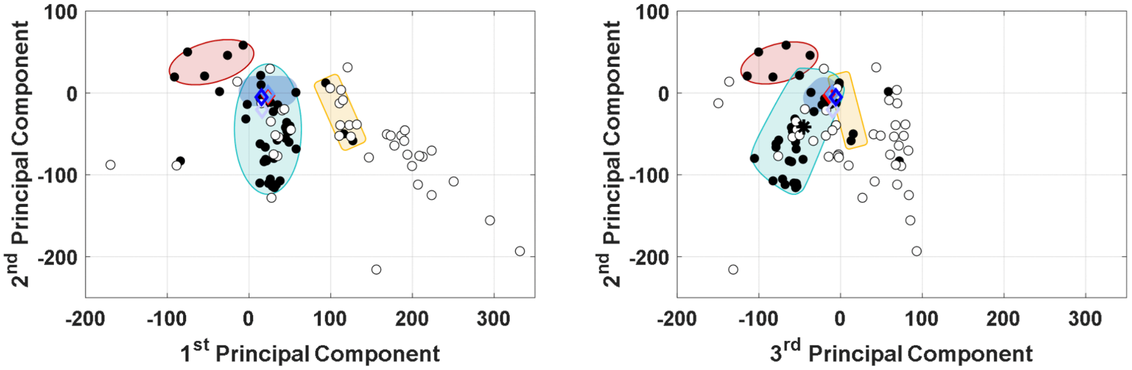

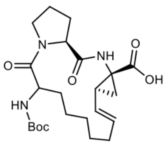

4a ◊

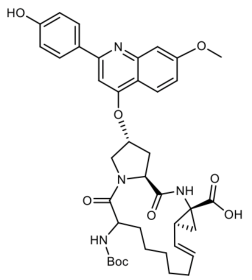

4c (%F = 2) ◊

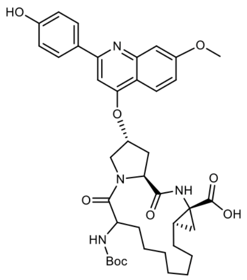

4d (%F = 22) ◊

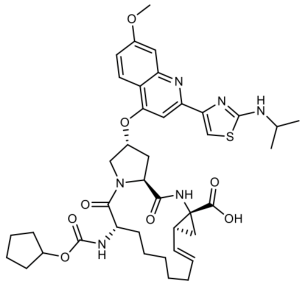

BILN 2061 (%F = 13) ◊

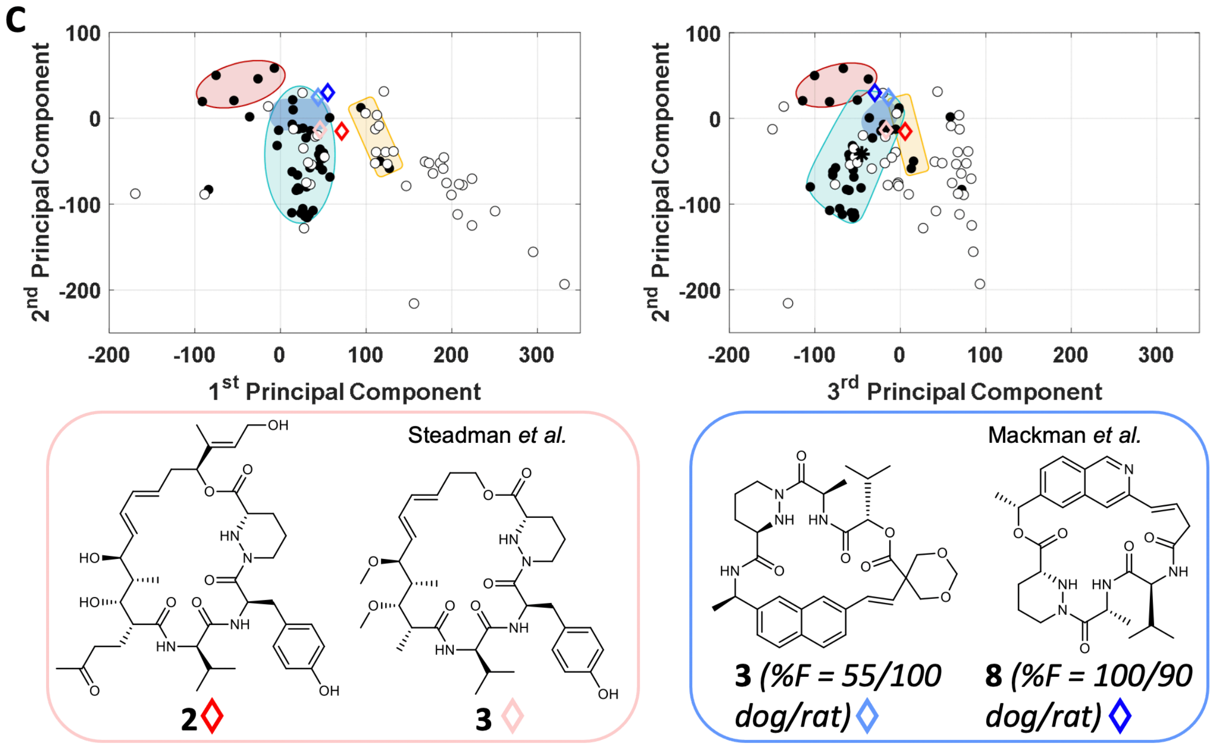

Figure S10. Historical examples of MC optimization efforts mapped onto MC Chemical Space. Selected compounds from optimization of **(A)** CXCR7 modulators from the work of Boehm *et al.* (*J. Med. Chem.* 2017, **60**, 9653-9663), **(B)** NS3 protease inhibitors from the work of Lamarre *et al.* (*Nature* 2003, **426**, 186) and Tsantrizos *et al.* (*Angew. Chem. Int. Ed. Eng.* 2003, **42**, 12), and **(C)** Sanglifehrin A-derived cyclophilin inhibitors from the work of Steadman *et al.* (*J. Med. Chem.* 2017, **60**, 1000) and Mackman *et al.* (*J. Med. Chem.* 2018, **61**, 9473) are plotted with respect to PC1 versus PC2 (left) and PC2 versus PC3 (right), and are overlaid with Zones 1-3 from Figure 3 (main text). In all cases, the compound numbering was retained from the original publications. These results may be compared with Figure 8 in the main text, which shows a similar analysis of the optimization of AMG176.

Table S1. Oral MC Drugs and Clinical Candidates with PCA scores, and zone designations.

	scores			
**Oral MC Drugs and CC**	**PC1 (x)**	**PC2 (y)**	**PC3 (z)**	**Zone**
AMG-176	14	21	-50	1
Azithromycin	13	-110	-57	1
Clarithromycin	26	-105	-71	1
Cyclosporine	116	-50	15	3
Danoprevir	21	-13	-5	1
Dirithromycin	38	-107	-83	1
Erythromycin	24	-110	-54	1
Erythromycin ethyl succinate	28	-114	-53	1
Everolimus	49	-60	-55	1
Flopristin	14	-7	-20	1
Flurithromycin ethyl succinate	31	-116	-55	1
Glecaprevir	57	1	-36	1
Grazoprevir	26	-12	-19	1
Ivermectin	34	-58	-76	1
Ixabepilone	-84	-83	72	Singleton
JNJ 26483327	-91	20	-83	2
Josamycin	18	-84	-60	1
Linopristin	94	12	-2	3
Lorlatinib	-7	58	-67	2
Mitemcinal	30	-80	-105	1
Moxidectin	-4	-32	-56	1
Pacritinib	-54	21	-114	2
Paritaprevir	15	10	-2	1
Pimecrolimus	46	-36	-53	1
Repotrectinib	-26	46	-37	2
Rifabutin	45	-42	-60	1
Rifampicin	46	-47	-56	1
Rifapentine	49	-47	-59	1
Rokitamycin	20	-81	-46	1
Roxithromycin	33	-112	-68	1
SB1317/TG02	-75	50	-100	2
SCY635	127	-58	13	3
Simeprevir	-2	-14	-12	1
Sirolimus	45	-55	-55	1
Solithromycin	20	-66	-79	1
Spiramycin	21	-83	-63	1
Tacrolimus	50	-40	-49	1
Telithromycin	14	-62	-79	1
Temsirolimus	58	-68	-54	1
TMC647055	-36	2	58	Singeton
Vaniprevir	35	-15	-23	1
Voxilaprevir	30	-23	-33	1

CC = Clinical Candidates; PC = Principal Component;

Table S2. Non-Oral MC Drugs and Clinical Candidates with PCA scores.

	scores		
Non-Oral MC Drugs and CC	PC1 (x)	PC2 (y)	PC3 (z)
Anidulafungin	148	-22	35
Argipressin	133	-53	74
Atosiban	123	-39	67
Bacitracin	175	-70	73
Bremelanotide	111	-13	69
Bryostatin 1	51	-45	-54
Capreomycin	110	-52	77
Carbetocin	117	-36	68
Caspofungin	169	-50	40
Colistimethate	224	-125	84
Colistin Sulfate	181	-72	71
Dactinomycin	112	-39	-6
Dalbavancin	186	-52	-18
Dalfopristin	41	-21	-18
Daptomycin	207	-112	69
Desmopressin	132	-39	79
Difimicin	35	-77	-76
Eptifibatide	111	-9	70
Eribulin	43	-20	-44
Lanreotide	115	-9	59
Latrunculin B	-89	-89	10
Linaclotide	194	-75	61
Lotilibcin	200	-89	74
Micafungin	173	-52	49
Murepavadin	224	-70	83
Myolimus	36	-54	-59
Natamycin	30	-75	-3
Nystatin	27	-128	27
Octreotide	112	-11	58
Oritavancin	190	-59	-33
OTX008	15	-13	-150
Oxytocin	125	-53	65
Pasireotide	121	31	44
Plecanatide	250	-108	42
Plerixafor	-170	-88	68
Polymyxin B	178	-64	71
Quinupristin	99	6	-4
Ramoplanin	295	-156	85
Rifamycin SV	32	-52	-50
Rifaximin	27	-35	-55
Somatoprim	113	4	69
Sugammadex	156	-216	-131
Teicoplanin	213	-78	-15
Telavancin	208	-77	-3
Terlipressin	160	-57	85
Thiostrepton	147	-79	-2
Trabectedin	26	30	-20
Tubocurarine	-14	14	-136
Urofollitropin	121	-51	59
Vancomycin	191	-46	-10
Vapreotide	111	0	58
Ziconotide	332	-193	93

CC = Clinical Candidates; PC = Principal Component;

Table S3. Property ranges and (for continuous variables) minimum and standard deviation values used to normalize the descriptor values, and the PCA coefficients calculated for each descriptor upon analysis of the collection of MC compound sets.

	Normalization Parameters			Coefficients from PCA Analysis									
Descriptor	Max of Property Values (P_{max})	Min of Property Values (P_{min})	Standard Deviation of Property Values (σ)	PC1	PC2	PC3	PC4	PC5	PC6	PC7	PC8	PC9	PC10
001_MW	-	212.15	328.73	0.22	-0.22	-0.01	0.16	0.10	0.11	-0.05	-0.09	0.00	0.00
002_HBD	42.00	-	-	0.10	-0.10	0.05	0.09	0.04	0.04	-0.02	0.04	0.06	0.03
003_HBD/HA	0.28	-	-	0.13	-0.11	0.12	0.05	0.04	-0.01	-0.05	0.09	0.11	0.10
004_HBA	48.00	-	-	0.10	-0.12	-0.01	0.07	0.03	0.06	0.01	0.04	0.03	-0.03
005_HBA/HA	0.57	-	-	0.04	-0.07	-0.02	0.00	-0.04	0.01	0.05	0.11	0.04	-0.11
006_CLogP	-	-16.57	3.50	-0.17	0.08	-0.20	-0.12	0.02	0.10	-0.23	-0.31	-0.10	0.00
007_Nrb	44.00	-	-	0.11	-0.13	0.02	0.10	0.10	0.04	0.04	-0.06	-0.03	0.01
008_PSA	-	0.00	144.22	0.28	-0.29	0.09	0.23	0.10	0.08	-0.03	0.07	0.10	-0.04
009_Largest Ring Size (N)	50.00	-	-	0.09	-0.12	0.01	0.03	-0.06	-0.11	-0.08	0.06	0.10	-0.01
010_Large Subs	8.00	-	-	0.08	-0.02	0.00	0.09	0.10	0.01	0.06	-0.08	-0.07	-0.12
011_Small Subs	11.00	-	-	0.08	-0.04	-0.06	0.02	-0.06	-0.13	0.03	-0.01	0.07	0.04
012_SubHA/HA	0.86	-	-	0.05	0.05	-0.07	0.13	0.20	0.22	0.14	-0.12	-0.14	0.03
013_SubCount/N	1.32	-	-	0.04	0.02	-0.05	0.01	0.03	-0.01	0.08	-0.04	-0.02	-0.03
014_PeriphCount/N	0.86	-	-	0.10	-0.14	0.00	-0.17	0.03	-0.13	-0.17	-0.23	-0.05	-0.07
015_(SubN+1)/(SubO+1)	6.00	-	-	-0.02	0.07	0.02	0.10	-0.05	-0.10	-0.08	0.01	0.09	0.25
016_(PeriphN+1)/(PeriphO+1)	1.00	-	-	-0.24	0.00	-0.18	0.30	0.05	0.05	0.25	0.18	0.02	0.09
017_(TotalN+1)/(TotalO+1)	9.00	-	-	-0.02	0.04	0.02	0.08	0.03	-0.06	0.02	0.00	0.00	0.17
018_ChiralCenters/HA	0.37	-	-	0.13	-0.19	-0.10	-0.27	-0.02	0.04	0.05	-0.03	-0.11	-0.02
019_RingChiralCenters/N	0.79	-	-	0.13	-0.14	-0.08	-0.22	0.02	-0.01	0.03	-0.12	-0.14	-0.05
020_N/HA	1.00	-	-	-0.07	0.00	0.05	-0.05	-0.17	-0.14	-0.05	0.17	0.13	-0.01
021_(SubHA+PeriphHA)/HA	0.88	-	-	0.07	0.01	-0.05	0.05	0.17	0.14	0.05	-0.17	-0.13	0.01
022_RingHet/N	0.50	-	-	0.08	0.07	0.08	0.14	-0.08	-0.17	0.21	0.09	0.00	-0.28
023_TotalHet/HA	0.71	-	-	0.05	-0.06	0.05	0.05	-0.05	-0.03	0.06	0.10	0.02	-0.12
024_RingOxygen/N	0.40	-	-	-0.03	-0.04	-0.08	-0.03	-0.21	0.06	0.12	0.10	0.10	-0.34
025_TotalOxygen/HA	0.38	-	-	0.06	-0.17	-0.02	-0.12	-0.09	0.08	0.03	0.10	0.05	-0.31
026_RingNitrogen/N	0.50	-	-	0.09	0.10	0.13	0.15	0.07	-0.23	0.10	0.01	-0.11	0.01
027_TotalNitrogen/HA	0.57	-	-	0.03	0.05	0.05	0.11	0.01	-0.10	0.03	0.05	0.00	0.09
028_PeriphFluorine/N	0.11	-	-	0.00	-0.01	-0.02	-0.02	0.01	0.00	-0.01	-0.02	-0.02	0.00
029_TotalFluorine/HA	0.22	-	-	-0.01	0.02	0.01	0.01	0.00	0.00	0.03	-0.02	-0.03	-0.01
030_PeriphChlorine/N	0.06	-	-	0.03	-0.02	0.00	0.05	-0.02	0.16	-0.06	0.03	0.00	0.04
031_TotalChlorine/HA	0.12	-	-	-0.01	0.00	0.01	0.02	-0.02	0.06	-0.05	-0.01	-0.02	-0.04
032_RingSulfur/N	0.20	-	-	0.02	0.00	0.03	0.02	0.02	0.01	0.03	-0.02	0.07	-0.05
033_TotalSulfur/HA	0.25	-	-	-0.01	-0.03	0.01	0.03	0.00	0.01	0.03	0.00	0.00	0.01
034_TotalAromatics/HA	0.71	-	-	-0.06	0.26	-0.04	0.25	0.07	0.08	-0.20	-0.03	0.01	-0.17
035_Spiro Rings	1.00	-	-	-0.01	0.11	0.00	-0.01	0.06	0.12	0.14	-0.35	0.81	0.06
036_FusedRings/N	1.00	-	-	0.04	0.04	-0.07	0.05	-0.09	0.13	-0.02	0.06	-0.02	0.06
037_Bridges	8.00	-	-	0.07	0.02	-0.09	0.02	-0.18	0.12	0.03	0.14	-0.01	0.11
038_SubN/SubHA	0.67	-	-	0.00	0.02	0.01	0.10	-0.01	-0.04	-0.02	0.06	0.07	0.13
039_SubO/SubHA	0.50	-	-	0.03	-0.19	-0.07	-0.12	0.02	0.14	0.04	0.15	0.01	-0.19
040_SubF/SubHA	0.40	-	-	-0.01	0.02	0.01	0.02	-0.01	0.00	0.03	-0.01	-0.03	-0.01
041_SubCl/SubHA	0.27	-	-	-0.01	0.00	0.01	0.01	-0.01	0.04	-0.03	0.00	-0.01	-0.04
042_SubS/SubHA	0.63	-	-	-0.02	-0.03	0.01	0.01	-0.01	0.00	0.02	0.00	-0.01	0.04
043_SubHeteroatoms/SubHA	1.00	-	-	0.00	-0.09	-0.01	0.03	-0.01	0.05	0.01	0.10	0.03	0.00
044_(RingN+1)/(RingO+1)	16.00	-	-	0.07	-0.01	0.10	0.12	0.09	-0.17	0.02	-0.03	-0.04	0.08
045_PeriphN/PeriphHA	0.17	-	-	0.02	-0.02	0.03	0.02	0.02	-0.04	0.01	0.02	0.02	0.02
046_PeriphO/PeriphHA	1.00	-	-	0.24	0.33	0.37	-0.25	0.02	0.23	0.07	0.06	0.00	-0.04
047_PeriphF/PeriphHA	0.50	-	-	0.00	0.00	-0.01	-0.01	0.00	0.00	-0.01	-0.01	-0.01	0.01
048_PeriphCl/PeriphH	0.33	-	-	0.03	-0.02	0.00	0.05	-0.02	0.14	-0.05	0.03	0.00	0.04
049_PeriphS/PeriphHA	0.00	-	-	0.00	0.00	0.00	0.00	0.00	0.00	0.00	0.00	0.00	0.00
050_PeriphHeteroatom/PeriphHA	1.00	-	-	0.25	0.32	0.36	-0.24	0.02	0.23	0.06	0.06	0.00	-0.03
051_Polar Surface Area/HA	9.09	-	-	0.08	-0.09	0.07	0.03	-0.01	-0.03	-0.01	0.11	0.06	-0.09
052_Carbonyls	26.00	-	-	0.10	-0.08	0.07	0.08	0.04	-0.05	-0.01	-0.03	0.00	0.02
053_Carboxylates	8.00	-	-	0.02	-0.04	0.00	0.03	0.00	0.04	0.03	0.06	0.03	-0.02
054_Alcohols	16.00	-	-	0.07	-0.11	-0.03	-0.03	0.03	0.10	0.00	0.05	0.05	-0.02
055_Phenols	5.00	-	-	0.06	-0.05	-0.01	0.06	0.02	0.17	-0.13	0.01	0.01	-0.04
056_Primary Amines	6.00	-	-	0.05	-0.05	0.05	0.06	0.03	-0.05	0.01	0.02	0.06	0.04
057_Secondary Amines	6.00	-	-	0.01	-0.03	0.02	0.03	0.00	0.05	0.02	0.01	0.00	0.07
058_Tertiary Amines	4.00	-	-	0.01	0.03	-0.09	-0.03	0.03	0.01	0.13	-0.02	-0.09	0.19
059_Primary Amides	3.00	-	-	0.06	-0.06	0.07	0.09	0.06	-0.02	0.00	0.01	0.03	-0.01
060_Secondary Amides	22.00	-	-	0.09	-0.05	0.09	0.11	0.04	-0.03	-0.03	-0.01	0.02	0.05
061_Tertiary Amides	7.00	-	-	0.03	0.05	0.00	0.02	-0.01	-0.13	0.12	-0.09	-0.11	0.03
062_Molecule Total Degree of Unsaturation	53.00	-	-	0.09	0.00	0.00	0.14	0.03	0.09	-0.12	-0.05	-0.02	-0.04
063_Peripheral carbonyls	16.00	-	-	0.11	-0.06	0.09	0.03	-0.01	-0.16	-0.07	-0.09	0.00	0.00
064_Substituent Fsp3	1.00	-	-	0.14	-0.17	-0.13	-0.29	-0.07	-0.08	0.23	-0.05	0.05	0.36
065_Macrocycle Fsp3	1.00	-	-	-0.10	-0.05	0.09	-0.06	0.18	0.03	0.37	0.03	-0.09	0.01
066_Molecule Fsp3	1.00	-	-	0.01	-0.15	0.00	-0.18	-0.02	-0.04	0.24	0.00	0.00	0.22
067_Max Substituent Size	91.00	-	-	0.06	-0.07	0.01	0.08	0.07	0.20	-0.03	-0.03	-0.03	0.08
068_Min Subsitutent Size	29.00	-	-	-0.11	-0.05	0.13	0.07	0.00	0.13	-0.02	-0.08	-0.13	0.04
069_Mean Substituent Size	37.67	-	-	0.00	-0.06	0.04	0.09	0.08	0.27	-0.04	-0.08	-0.11	0.08
070_Substituent Size st. dev	47.06	-	-	0.07	-0.04	-0.03	0.04	0.05	0.15	-0.03	0.00	0.01	0.06
071_Substituent Size Distribution	1.76	-	-	0.14	-0.02	-0.10	0.00	0.09	0.06	0.00	-0.02	0.08	0.10
072_Max Gap Size	34.00	-	-	-0.06	-0.09	0.07	-0.07	0.04	-0.03	-0.16	0.12	0.07	0.05
073_Min Gap Size	18.00	-	-	-0.15	-0.15	0.22	0.04	-0.21	0.05	-0.02	-0.08	-0.01	0.00
074_Mean Gap Size	18.00	-	-	-0.14	-0.15	0.19	-0.04	-0.09	0.02	-0.14	0.07	0.06	0.04
075_Gap Size st dev	16.00	-	-	0.02	-0.01	-0.04	-0.09	0.16	-0.05	-0.15	0.18	0.07	0.05
076_Max Gap Size/N	1.00	-	-	-0.21	-0.11	0.20	-0.10	0.17	0.04	-0.20	0.16	0.02	0.14
077_Min Gap Size/N	1.00	-	-	-0.22	-0.18	0.30	0.06	-0.24	0.12	0.00	-0.14	-0.05	0.08
078_Mean Gap Size/N	1.00	-	-	-0.22	-0.13	0.27	-0.01	-0.06	0.09	-0.08	0.00	-0.01	0.10
079_St dev of Gap Size/N values	0.42	-	-	0.00	0.08	-0.10	-0.17	0.46	-0.09	-0.24	0.38	0.06	0.08
080_Bridging atoms	22.00	-	-	0.05	-0.02	-0.02	0.05	-0.07	0.07	0.02	0.10	0.03	0.07
081_Bridging atoms/N	0.45	-	-	0.11	0.01	-0.09	0.08	-0.20	0.21	0.03	0.18	0.00	0.17
082_Restricted fraction	0.66	-	-	0.18	0.12	-0.09	0.04	-0.29	0.05	-0.12	0.11	-0.09	0.18
083_Non-restricted fraction	1.00	-	-	-0.18	-0.12	0.09	-0.04	0.29	-0.05	0.12	-0.11	0.09	-0.18
084_MC amide bonds (total)	15.00	-	-	0.11	-0.03	0.11	0.09	0.00	-0.16	-0.02	-0.07	0.00	0.03
085_Ring Complexity without heteroatoms	1.00	-	-	0.16	-0.01	-0.11	-0.01	-0.13	-0.06	-0.25	-0.15	0.07	-0.04
086_Ring Complexity with heteroatoms	1.00	-	-	0.13	0.01	-0.14	-0.02	-0.11	-0.05	-0.20	-0.13	0.04	-0.05
087_Substituent Flexibility Fraction	1.00	-	-	0.00	-0.11	0.08	-0.04	0.00	-0.09	0.01	-0.01	-0.02	-0.01
088_Substituent Rotation	9.00	-	-	0.04	0.09	-0.10	0.06	-0.12	0.01	0.13	0.10	0.06	0.02
089_Peptide character index	1.00	-	-	0.19	0.07	0.23	0.10	-0.02	-0.25	-0.02	-0.12	-0.11	0.06
090_Charges	4.00	-	-	-0.01	-0.01	-0.02	0.02	-0.02	0.00	-0.01	0.03	0.01	-0.06

Table S4

Table S4A. Properties with absolute magnitude of coefficients (loadings) > 0.16 in PC1

Descriptors	Loadings
(8) PSA	0.28
(50) PeriphHeteroatom/PeriphHA	0.25
(16) (PeriphN+1)/(PeriphO+1)	-0.24
(46) PeriphO/PeriphHA	0.24
(78) Mean Gap Size/N	-0.22
(1) MW	0.22
(77) Min Gap Size/N	-0.22
(76) Max Gap Size/N	-0.21
(89) Peptide character index	0.19
(82) Restricted fraction	0.18
(83) Non-restricted fraction	-0.18
(6) CLogP	-0.17
(85) Ring Complexity without heteroatoms	0.16

Table S4B. Properties with absolute magnitude of coefficients (loadings) > 0.16 in PC2

Descriptors	Loadings
(46) PeriphO/PeriphHA	0.32
(50) PeriphHeteroatom/PeriphHA	0.32
(8) PSA	-0.30
(34) TotalAromatics/HA	0.26
(1) MW	-0.22
(39) SubO/SubHA	-0.19
(18) ChiralCenters/HA	-0.19
(77) Min Gap Size/N	-0.18
(25) TotalOxygen/HA	-0.17
(64) Substituent Fsp3	-0.17

Table S5. Distance of each compound design from Figure 6 from the center of mass of the Oral MC Drugs set.

Design	Distance from Center of Oral MC Drugs
**1**	227
**2**	197
**3**	78
**4**	187
